# Global burden and trends of appendicitis among adolescents and young adults: A systematic analysis for the Global Burden of Disease study 2021 and predictions to 2040.

**DOI:** 10.1097/MD.0000000000049625

**Published:** 2026-07-03

**Authors:** Qian Sun, Ming Yan, Han Wang, Tao Hu, Xinghao Chen, Shun Xu

**Affiliations:** a Department of Emergency, The Affiliated Jiangning Hospital of Nanjing Medical University, Nanjing, Jiangsu, China; b Department of Surgery, The Affiliated Jiangning Hospital of Nanjing Medical University, Nanjing, Jiangsu, China.

**Keywords:** adolescents and young adults, appendicitis, disease burden, prediction

## Abstract

Appendicitis is a prevalent abdominal disease with significant global distribution. This study aimed to assess the prevalence and burden of appendicitis by examining trends in incidence, mortality, and disability-adjusted life years (DALYs) from 1990 to 2021 among adolescents and young adults (AYAs). Data on appendicitis from the Global Burden of Disease database (1990–2021) were utilized. Point estimates with a 95% uncertainty interval (UI) were calculated to evaluate incidence rates, death rates, and DALY rates for individuals aged 15 to 39 years. Estimated annual percentage changes (EAPCs) were used to evaluate trends in age-standardized rates with a 95% confidence interval (CI). Future projections were generated using the Bayesian age–period–cohort model. In 2021, the global incidence of appendicitis among AYAs exceeded 10 million cases, with the highest incidence rate observed in the 20 to 24 age group. Incidence rates were higher among females. Between 1990 and 2021, the global incidence rate decreased from 391.46 (95% UI: 290.72–520.72) to 368.43 (95% UI: 279.00–488.48) per 100,000 population, with an EAPC of −0.27 (95% CI: −0.34–−0.20). Death rates and DALY rates also showed declines, with EAPCs of −3.01 (95% CI: −3.23–−2.78) and −2.59 (95% CI: −2.78–−2.40), respectively. At the national level, India reported the highest number of cases in 2021 (2972,773.19; 95% UI: 2334,072.00–3778,073.13). The burden of appendicitis among AYAs decreased in most countries and territories, with the Republic of Armenia showing the most notable reduction in death rates from 1990 to 2021. Regional analyses indicated that high-middle socio-demographic Index (SDI) regions had the highest incidence rates in 2021, while most SDI regions experienced declining trends in death and DALY rates over the study period. Projections using the Bayesian age–period–cohort prediction model suggest that the incidence and DALY rates of appendicitis among AYAs will continue to decrease from 2021 to 2040. Appendicitis remains a substantial public health burden among AYAs due to its high incidence. Despite significant global declines in deaths and DALYs, the absolute numbers of deaths and DALYs remain substantial, particularly in low-middle and low SDI regions. Effective and targeted medical and health policies are essential to reduce the burden of appendicitis in AYAs.

## 1. Introduction

Appendicitis is a common abdominal surgical disease associated with significant social and economic burdens, with a lifetime incidence risk of acute appendicitis estimated at 7 to 8% in the general population.^[1]^ A systematic review indicated that while the incidence of appendicitis remains stable in most Western countries, it is increasing in newly industrialized nations.^[2]^ Symptoms of appendicitis vary among patients^[3]^; its typical clinical manifestations include right lower abdominal pain and fever. Acute appendicitis can lead to severe complications such as perforation, peritonitis, and septic shock, posing life-threatening risks to patients. Chronic appendicitis, on the other hand, significantly affects patients’ quality of life and that of their families. Persistent abdominal pain can result in sleep disruption, reduced work or study efficiency, and negative impacts on social life, placing substantial stress on healthcare systems.^[4]^ Imaging techniques such as computed tomography (CT) scans have shown high diagnostic accuracy for acute appendicitis.^[5]^ Despite advancements in surgical techniques and antibiotic therapies that have significantly reduced global appendicitis mortality rates over the past 3 decades, certain regions still experience high death rates.^[6]^

Previous studies have identified age as an independent factor influencing the risk of complicated acute appendicitis.^[7,8]^ Adolescents and young adults (AYAs), defined as individuals aged 15 to 39 years, represent a unique subgroup within the Global Burden of Disease (GBD) 2021 study population. This group is in a critical life stage characterized by significant educational and professional milestones. Compared with older populations, appendicitis in AYAs has a greater impact on work and study productivity.^[9]^ As such, addressing appendicitis among AYAs is an essential component of efforts to reduce the burden of noncommunicable diseases in this demographic.

Clinical treatment of appendicitis imposes a significant economic burden globally.^[10]^ A study conducted in China identified acute appendicitis as the fifth most economically burdensome disease in 2013.^[11]^ With advancements in healthcare infrastructure and computer-based information systems, data on the burden of appendicitis have become more detailed, enabling more accurate predictions of temporal trends across different age groups.

Most existing research on appendicitis has been based on single-center studies^[12,13]^ or lacks specific analyses by age group.^[14]^ Given this context, a comprehensive assessment of the disease burden of appendicitis among AYAs is essential. The GBD 2021 database offers a robust platform for integrating diverse data sets while improving methodological consistency and standardization. This study utilized data from GBD 2021 to systematically analyze trends in appendicitis incidence, mortality, and disability-adjusted life years (DALYs) among AYAs aged 15 to 39 years. The analysis was conducted across different age groups, genders, regions, and nations to provide a scientific basis for formulating more effective public health strategies targeting appendicitis.

## 2. Methods

### 2.1. Data source and overview

This study adheres to the GATHER checklist (https://www.who.int/publications/m/item/gather-checklist) and is registered in the GBD 2021 Paper Proposal Form (https://uwhealthmetrics.co1.qualtrics.com/). Data were obtained from the Global Health Data Exchange platform (https://vizhub.healthdata.org/gbd-results/). The GBD 2021 comprehensively evaluated the burden of 371 diseases and injuries across 204 countries and territories from 1990 to 2021.^[15]^ We extracted data on the burden of appendicitis among AYAs from the GBD 2021 database. The study recorded the number of cases, deaths, and DALYs associated with appendicitis, as well as the corresponding rates per 100,000 population.

AYAs were defined as individuals aged 15 to 39 years.^[16]^ Appendicitis was characterized as inflammation of the appendix, presenting symptoms such as nausea, vomiting, and sharp pain in the right lower abdomen. Clinical symptoms may vary between patients.^[17]^ According to the International Classification of Diseases versions 9 (ICD-9) and 10 (ICD-10), appendicitis includes acute appendicitis (ICD-9: 540; ICD-10: K35), unspecified appendicitis (ICD-9: 541; ICD-10: K37), and other appendicitis (ICD-9: 542; ICD-10: K36).^[18]^ DALYs were employed to measure the burden of appendicitis, combining years of life lost due to premature mortality and years lived with disability. For all metrics, 95% uncertainty intervals (UIs) were calculated using the 25th and 75th ordered 1000 draw values from the posterior distribution.^[16]^ Parameters for analysis included specific age groups (15–39 years), gender (both sexes), and the study period encompassing all years from 1990 to 2021. The defined age range was further divided into 5-year intervals: 15 to 19, 20 to 24, 25 to 29, 30 to 34, and 35 to 39 years. Data were also categorized by region and by individual countries or territories to ensure comprehensive analysis.

### 2.2. Socio-demographic index (SDI)

The 2021 GBD Study employed the SDI to assess the impact of social and economic factors on health outcomes across countries.^[6]^ The regional classification of SDI can be accessed from the Institute for Health Metrics and Evaluation (https://ghdx.healthdata.org/search/site/SDI). SDI is calculated as the geometric mean of 3 indicators: total fertility rate, per-capita income, and average years of schooling among individuals aged 15 years and older. SDI values range from 0 to 1, with higher values indicating greater socioeconomic development. SDI has been reported to influence disease prognosis.^[19]^ Countries and territories are categorized into 5 SDI levels based on these values: low (< 0.46), low-middle (0.46–0.60), middle (0.61–0.69), high-middle (0.70–0.81), and high (> 0.81).^[7]^ In this study, countries and regions were classified into high, high-middle, middle, middle-low, and low SDI groups. The relationship between SDI levels and the burden of appendicitis in AYAs (15–39 years) was analyzed.

### 2.3. Statistical analysis

Percentage changes in the rates of incidence, deaths, and DALYs from 1990 to 2021 were calculated.^[20]^ Linear regression analysis was used to compute the estimated annual percentage changes (EAPCs) and their 95% confidence interval (CI)s. The EAPC was calculated as 100 × (exp(β) − ). EAPCs were utilized to assess trends in incidence, deaths, and DALYs over time. When the EAPC and its 95% CI include 0, the change is considered statistically nonsignificant. If the EAPC and the lower bound of its 95% CI are > 0, the rate shows a statistically significant increasing trend. If the upper limit of the EAPC and its 95% CI is < 0, the rate shows a statistically significant decreasing trend. Associations between EAPC and SDI levels were evaluated using scatter plots and Pearson correlation analysis. The Bayesian age–period–cohort (BAPC) model was applied to predict the incidence and DALY rates from 2021 to 2040, using the R packages BAPC and INLA. The BAPC model offers high accuracy in forecasting disease burden. It incorporates an integrated nested Laplacian approximation for projecting prevalence rates over time. All statistical analyses were conducted using R software (version 4.4.1, R Foundation for Statistical Computing, https://cran.r-project.org). A *P* < .05 was considered statistically significant.

### 2.4. Patient and public involvement

The data analyzed in this study were publicly available from the published GBD study covering 1990 to 2021, with no identifiable personal information. Consequently, informed consent was waived. This study adhered to the Strengthening the Reporting of Observational Studies in Epidemiology reporting guidelines. Neither patients nor the public were involved in the study design, data interpretation, manuscript drafting, or dissemination plans.

## 3. Result

### 3.1. The global burden and time trends in appendicitis for AYAs from 1990 to 2021

The burden of appendicitis in AYAs, along with its trends at the global, national, and regional levels, is detailed in Table S1, Supplemental Digital Content 1 and Table S2, Supplemental Digital Content 2, (Supplemental Digital Content). Globally, incident cases of appendicitis among AYAs increased from 8,580,076.32 (95% UI: 372,151.04–11,413,200.96) in 1990 to 10,960,139.89 (95% UI: 8,299,778.96–14,531,553.31) in 2021. However, the corresponding incidence rate declined from 391.46 (95% UI: 290.73–520.72) per 100,000 in 1990 to 368.43 (95% UI: 279.00–488.48) per 100,000 in 2021, with an EAPC of −0.27 (95% UI: −0.34–−0.20). Over the past 30 years, the global incidence rates of appendicitis decreased by −5.88% (95% UI: −9.17%–−2.14%). Between 1990 and 2021, deaths due to appendicitis among AYAs decreased from 11,509.59 (95% UI: 8989.49–15,268.25) in 1990 to 6736.71 (95% UI: 5623.45–8112.33) in 2021. Similarly, the death rate of appendicitis dropped by −56.88% (95% UI: −66.65%–−42.77%), from 0.53 (95% UI: 0.41–0.70) per 100,000 in 1990 to 0.23 (95% UI: 0.19–0.27) per 100,000 in 2021, with an EAPC of −3.01 (95% CI: −3.23–−2.78). The DALY rate for appendicitis also declined from 38.28 (95% UI: 30.80–48.99) per 100,000 in 1990 to 18.74 (95% UI: 15.59–22.91) per 100,000 in 2021, with an EAPC of −2.59 (95% CI: −2.78–−2.40).

**Figure 1.**
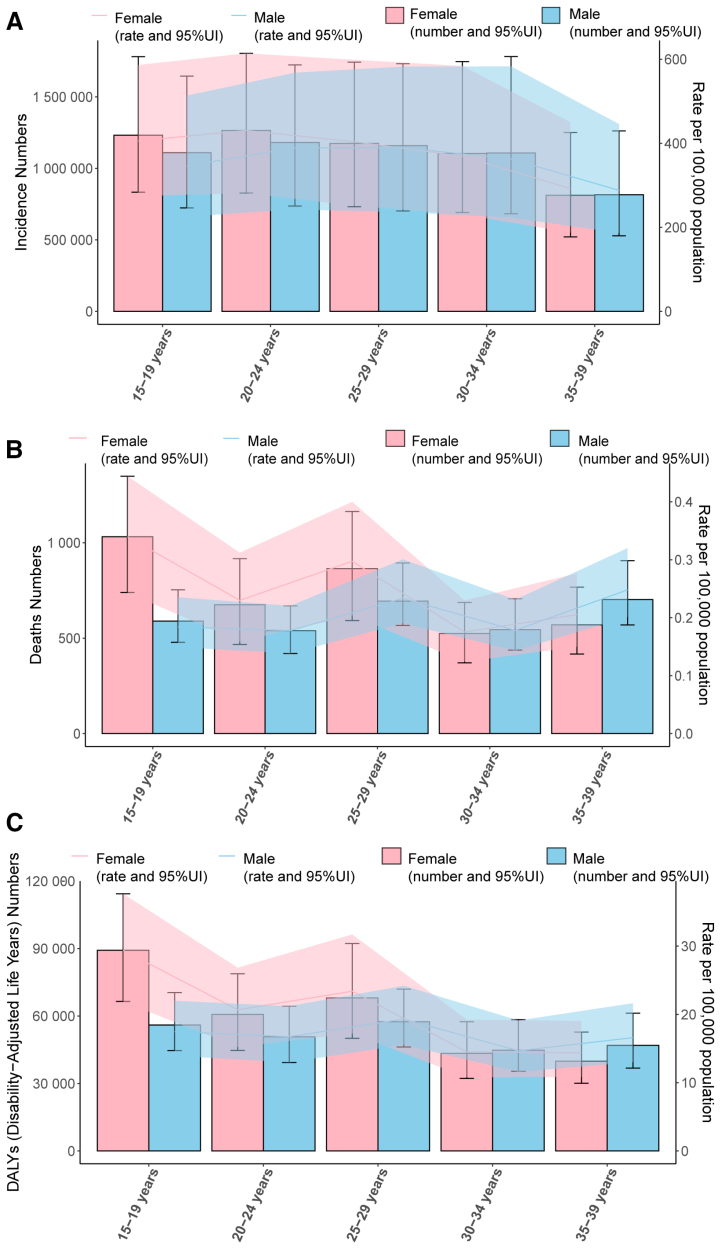
The Prevalence number and rates of appendicitis among AYAs based on different gender and age groups. In an age group, the left shows female and the right shows male. (A) Incidence number and rates, (B) Death number and rates, (C) DALYs number and rates. AYAs = adolescents and young adults, DALY = disability-adjusted life years, UI = uncertainty interval.

We also analyzed incidence rates, death rates, and DALY rates by gender across 5 age groups (15–39 years) in 2021 (Fig. 1). The highest incidence rate occurred in the 20 to 24 age group, with higher rates observed in females across all age groups. Death rates and DALY rates were highest for females aged 15 to 19 years, while males exhibited the highest death rates and DALY rates in the 25 to 29 age group. Women aged 15 to 29 years had higher death and DALY rates compared to their male counterparts (Fig S1, Supplemental Digital Content 3, Supplemental Digital Content).

### 3.2. Burden of appendicitis in AYAs at the regional level

**Figure 2.**
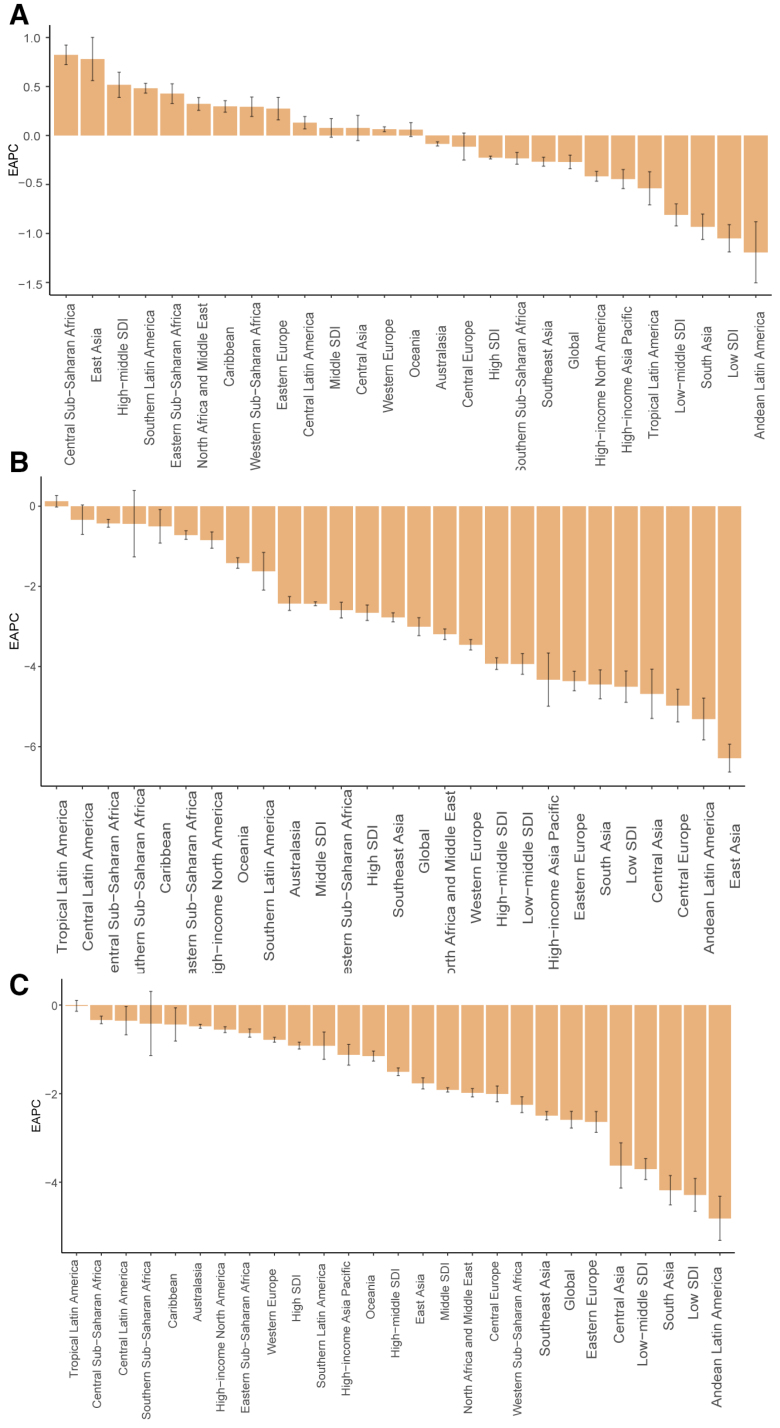
The EAPC of (A) incidence, (B) death and (C) DALYs rates of appendicitis among AYAs at the global, SDI and regional levels. AYAs = adolescents and young adults, DALY = disability-adjusted life years, EAPC = estimated annual percentage changes, SDI = socio-demographic index.

In 2021, among 21 geographic regions, South Asia recorded the largest number of appendicitis cases (3,736,019.78; 95% UI: 2,921,352.27–4,756,735.85), while Andean Latin America had the highest incidence rate (630.12; 95% UI: 462.21–835.33 per 100,000) (Table 1). Between 1990 and 2021, incidence rates increased in 12 GBD regions and decreased in 9 regions. The greatest increase was observed in Central Sub-Saharan Africa (EAPC = 0.82; 95% CI: 0.72–0.92), while the largest decrease occurred in Andean Latin America (EAPC = −1.19; 95% CI: −1.50–−0.88) (Fig. 2).

**Table 1:** Incidence of appendicitis in AYAs between 1990 to 2021 at the Global, Sex and Regional level, and PC from 1990 to 2021.

Location/Sex	1990			2021		1990–2021	
	Incidence cases (95% UI)	Incidence Rate (95% UI)	Incidence cases (95% UI)		Incidence Rate (95% UI)	PC (95% UI)	EAPC (95% CI)
Sex							
Male	3,994,031.93 (2,912,039.86–5,359,320.73)	360.32 (262.71–483.49)	5,372,893.37 (4,001,081.14–7,184,852.76)		355.89 (265.04–475.91)	−1.23 (−4.54–2.52)	−0.06 (−0.14–0.01)
Female	4,586,044.39 (3,434,786.92–6,045,247.22)	423.33 (317.06–558.02)	5,587,246.53 (4,260,306.39–7,367,344.16)		381.35 (290.78–502.85)	−9.92 (−13.23–−6.15)	−0.47 (−0.25–−0.39)
Global	8,580,076.32 (6,372,151.04–11413200.96)	391.46 (290.73–520.72)	10,960,139.89 (8,299,778.96–14,531,553.31)		368.43 (279.00–488.48)	−5.88 (−9.17–−2.14)	−0.27 (−0.34–−0.20)
SDI							
High SDI	1,475,555.48 (1,041,396.82–2,030,391.46)	425.28 (300.15–585.19)	1,403,559.13 (1,073,046.63–1,817,363.39)		397.34 (303.77–514.48)	−6.57 (−13.17–2.75)	−0.23 (−0.24–−0.21)
High-middle SDI	1,704,066.35 (1,179,063.00–2,357,089.23)	376.558 (260.55–520.86)	1,839,323.93 (1,335,406.68–2,544,320.76)		417.78 (303.32–577.90)	10.95 (4.33–18.96)	0.52 (0.39–0.65)
Middle SDI	2,842,221.74 (2,078,382.65–3,803,917.05)	377.64 (276.15–505.42)	3,590,731.91 (2,642,021.80–4,887,344.85)		387.15 (284.86–526.94)	2.52 (−2.53–7.80)	0.08 (−0.02–0.17)
Low-middle SDI	1,986,059.88 (1,578,514.21–2,503,622.27)	438.04 (348.15–552.19)	3,009,570.32 (2,330,710.65–3,855,450.07)		375.03 (290.43–480.43)	−14.38 (−18.14–−9.92)	−0.81 (−0.92–−0.70)
Low SDI	566,487.05 (446,486.77–715,823.50)	307.36 (242.25–388.38)	1,110,295.66 (845,217.15–1,446,592.73)		247.25 (188.22–322.14)	−19.56 (−24.02–−15.74)	−1.50 (−1.19–−0.91)
Regions							
High-income Asia Pacific	461,123.97 (319,952.58–639,397.47)	683.20 (474.04–947.32)	306,482.20 (223,573.29–412,422.23)		606.42 (442.37–816.04)	−11.24 (−16.68–−4.85)	−0.45 (−0.54–−0.35)
Central Asia	77,362.77 (55,922.44–104,901.66)	271.89 (196.54–368.68)	104,337.15 (73,235.94–145,644.09)		279.08 (195.89–389.56)	2.64 (−3.26–9.45)	0.08 (−0.05–0.21)
East Asia	2,420,936.42 (1,677,521.32–3,352,728.66)	427.95 (296.54–592.66)	2,348,421.25 (1,693,560.28–3,269,400.56)		490.23 (353.53–682.48)	14.552 (6.04–24.50)	0.78 (0.56–1.00)
South Asia	2,420,369.99 (1,936,143.79–3,031,777.67)	255.13 (185.95–343.27)	3,736,019.78 (2,921,352.27–4,756,735.85)		243.17 (176.37–327.26)	−4.69 (−7.78–−1.35)	−0.27 (−0.31–−0.22)
Southeast Asia	502,625.28 (366,322.51–676,260.00)	255.13 (185.95–343.27)	674,381.43 (489,107.50–907,567.25)		243.17 (176.37–327.26)	−4.69 (−7.78–−1.35)	−0.27 (−0.31–−0.22)
Australasia	40,480.69 (27,639.46–56,152.06)	496.46 (338.97–688.66)	49,839.49(34,812.10–67,864.05)		475.98 (332.47–648.12)	−4.13 (−9.340–1.13)	−0.09 (−0.11–−0.06)
Caribbean	42,335.15 (31,033.03–57,160.61)	284.80 (208.77–384.54)	59,768.17 (44,103.12–79,096.36)		328.35 (242.29–434.53)	15.29 (9.84–20.79)	0.30 (0.24–0.36)
Central Europe	156,683.76 (110,782.03–218,740.00)	334.45 (236.47–466.91)	120,260.37 (93,759.93–153,720.83)		343.41 (267.73–438.96)	2.68 (−8.47–16.67)	−0.11 (−0.25–0.03)
Eastern Europe	248,551.51 (174,687.87–350,235.34)	289.79 (203.67–408.35)	205,816.85 (146,160.25–296,109.92)		311.03 (220.88–447.48)	7.33 (1.90–13.92)	0.27 (0.16–0.39)
Western Europe	520,826.78 (358,895.00–717,479.80)	361.38 (249.03–497.84)	484,238.77 (351,598.77–652,558.31)		373.14 (270.93–502.85)	3.25 (−1.65–9.51)	0.06 (0.04–0.09)
Andean Latin America	112,389.85 (95,357.60–132,477.15)	726.80 (616.66–856.70)	170,632.71 (125,165.29–226,202.51)		630.12 (462.21–835.33)	−13.30 (−27.84–2.39)	−1.19 (−1.50–−0.88)
Central Latin America	214,907.71 (161,618.50–283,204.43)	314.80 (236.74–414.85)	350,484.31 (259,817.80–468,541.73)		346.46 (256.83–463.16)	10.06 (4.88–15.50)	0.13 (0.07–0.19)
Southern Latin America	59,654.81 (41,725.76–83,326.63)	312.670 (218.70–436.74)	98,131.40 (69,668.94–138,443.29)		380.42 (270.08–536.70)	21.67 (14.49–30.58)	0.48 (0.43–0.53)
Tropical Latin America	226,401.08 (172,902.30–287,907.70)	352.03 (268.85–447.67)	266,905.52 (203,974.31–342,913.75)		302.24 (230.98–388.31)	−14.14 (−17.71–−10.19)	−0.54 (−0.71–−0.37)
North Africa and Middle East	400,486.64 (274,515.34–560,197.36)	299.251 (205.12–418.59)	863,773.61 (605,619.57–1,209,560.53)		339.71 (238.18–475.71)	13.52 (8.05–20.72)	0.32 (0.26–0.39)
High-income North America	413,694.97 (302,948.96–559,712.29)	365.08 (267.35–493.94)	400,541.54 (333,798.97–481,859.33)		325.16 (270.98–391.17)	−10.94 (−24.92–7.14)	−0.42 (−0.47–−0.37)
Oceania	4559.51 (3145.30–6243.28)	171.64 (118.41–235.03)	10,278.00 (7091.40–14,175.60)		182.42 (125.86–251.59)	6.28 (1.10–11.37)	0.06 (−0.01–0.13)
Central Sub-Saharan Africa	26,115.35 (18,734.25–35,848.48)	125.78 (90.23–172.66)	88,934.12 (62,151.51–121,858.47)		164.40 (114.89–225.26)	30.70 (24.46–37.97)	0.82 (0.72–0.92)
Eastern Sub-Saharan Africa	92,566.44 (66,003.81–127,684.80)	130.58 (93.11–180.12)	272,059.87 (190,367.64–372,038.41)		155.30 (108.67–212.37)	18.93 (15.34–23.81)	0.43 (0.33–0.53)
Southern Sub-Saharan Africa	45,116.50 (31,973.70–61,217.07)	208.73 (147.92–283.21)	68,709.43 (49,300.22–93,000.14)		201.87 (144.85–273.24)	−3.28 (−7.75–1.37)	−0.23 (−0.29–−0.17)
Western Sub-Saharan Africa	92,887.18 (65,312.36–128,741.17)	129.78 (91.25–179.87)	280,123.94 (192,348.64–391,243.62)		146.50 (100.60–204.62)	12.89 (9.69–15.74)	0.29 (0.19–0.39)

AYAs = adolescents and young adults, EAPC = estimated annual percentage changes, PC = percentage change, SDI = socio-demographic index, UI = uncertainty interval.

Among AYAs, South Asia also had the highest number of deaths (3992.57; 95% UI: 3117.73–5176.13) and the highest death rate (0.51; 95% UI: 0.39–0.65 per 100,000) in 2021. (Table S1, Supplemental Digital Content 1, Supplemental Digital Content). Additionally, South Asia reported the highest number of DALYs (299,146.58; 95% UI: 239,230.25–379,842.72) and the highest DALY rate (37.82; 95% UI: 30.25–48.03 per 100,000) in 2021 (Table S2, Supplemental Digital Content 2, Supplemental Digital Content). Most GBD regions showed a declining trend in death rates and DALY rates, with the largest reduction in death rates observed in East Asia (EAPC = −6.29; 95% CI: −6.63–−5.94) and the greatest decrease in DALY rates found in Andean Latin America (EAPC = −4.82; 95% CI: −5.31–−4.32).

At the regional level in 2021, the highest incidence rate among females was in Andean Latin America (676.11; 95% UI: 491.69–896.18 per 100,000), while the highest incidence rate for males was observed in High-income Asia Pacific (590.35; 95% UI: 424.62–805.18 per 100,000). Detailed information is provided in Fig S2, Supplemental Digital Content 4 (Supplemental Digital Content). Conversely, the lowest incidence rate among females was reported in Eastern Sub-Saharan Africa (144.31; 95% UI: 99.79–200.14 per 100,000), and the lowest incidence rate for males was seen in Western Sub-Saharan Africa (136.37; 95% UI: 92.68–191.97 per 100,000). In 2021, South Asia recorded the highest death rate (0.65; 95% UI: 0.45–0.92 per 100,000) and DALY rate (48.28; 95% UI: 34.64–67.81 per 100,000) among females. The highest death rate for males was found in Central Latin America (0.42; 95% UI: 0.37–0.46 per 100,000), while the highest DALY rate among males was recorded in Andean Latin America (30.44; 95% UI: 24.00–40.90 per 100,000) (Fig. 2).

### 3.3. Burden of appendicitis at the national level

**Figure 3.**
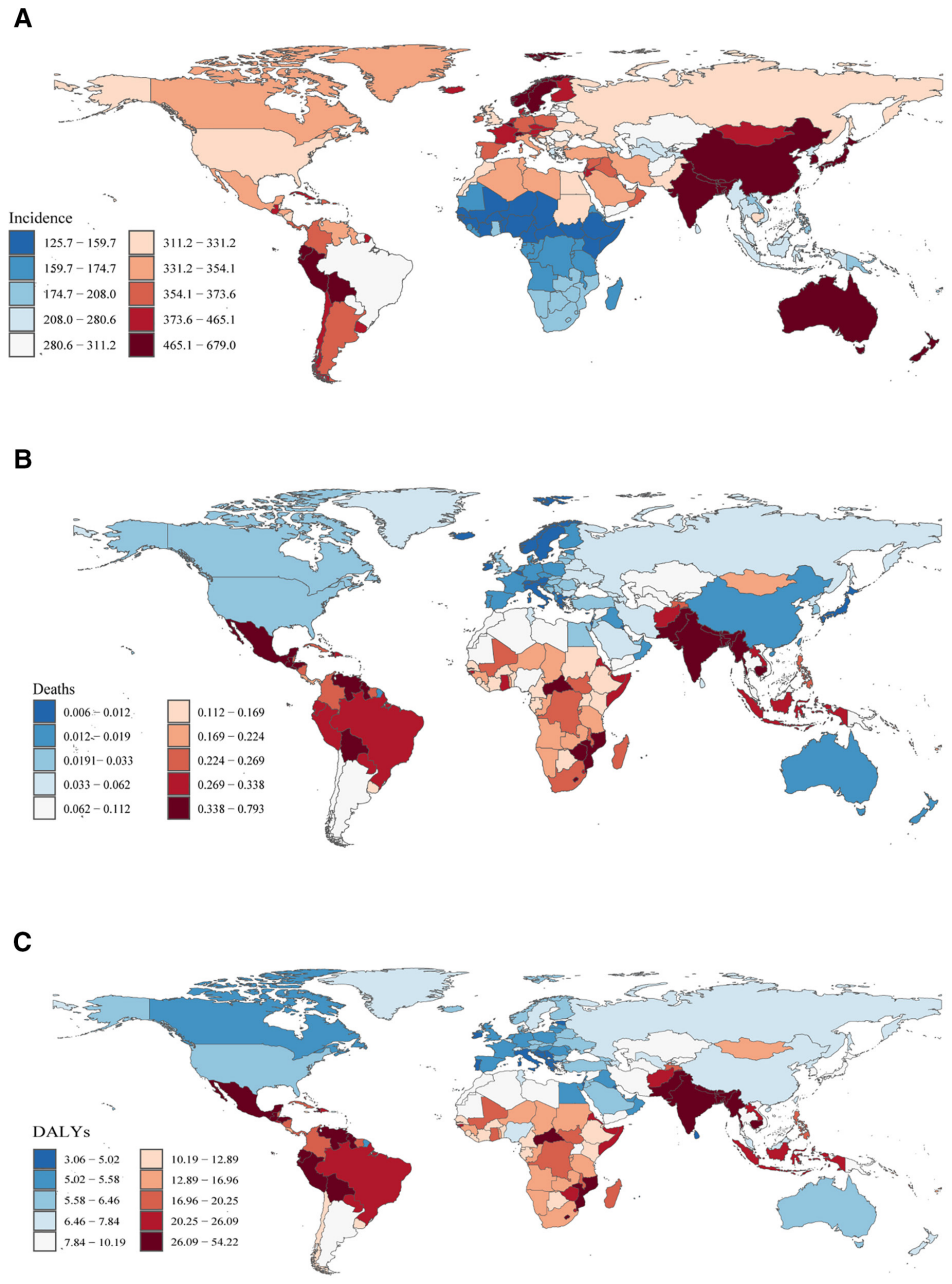
Global disease burden of appendicitis among AYAs in 204 countries and territories. (A) Incidence rate of appendicitis in 2021. (B) Death rate of appendicitis in 2021. (C) DALY rate of appendicitis in 2021. AYAs = adolescents and young adults, DALY = disability-adjusted life years.

The burden of appendicitis and its trends across 204 countries and territories are presented in Tables S3–S5, Supplemental Digital Content 5, (Supplemental Digital Content). In 2021, India reported the highest number of cases (2,972,773.19; 95% UI: 2,334,072.00–3,778,073.13), while the Republic of Peru recorded the highest incidence rate (678.99; 95% UI: 485.84–909.99 per 100,000). Conversely, the Federal Democratic Republic of Ethiopia had the lowest incidence rate globally (125.69; 95% UI: 88.81–174.12). As shown in Figure 3, between 1990 and 2021, Taiwan (Province of China) experienced the fastest increase in incidence rates (EAPC = 1.32; 95% CI: 1.27–1.32), whereas the Republic of Guatemala showed the largest decrease (EAPC = −2.46; 95% CI: −2.82–−2.09). In 2021, the Republic of Cyprus had the lowest death rate (0.01; 95% UI: 0.00–0.01 per 100,000), while the Republic of Guatemala recorded the highest death rate (0.79; 95% UI: 0.66–0.95 per 100,000). The highest DALY rate was observed in the Republic of Guyana (47.00; 95% UI: 35.77–60.75 per 100,000). From 1990 to 2021, most countries and territories experienced reductions in death and DALY rates, with the Republic of Armenia (EAPC = −6.50; 95% CI: −7.54–−5.46) and the Republic of Azerbaijan (EAPC = −6.46; 95% CI: −7.23–−5.70) showing the most significant decreases.

### 3.4. Burden of appendicitis by SDI regions

**Figure 4.**
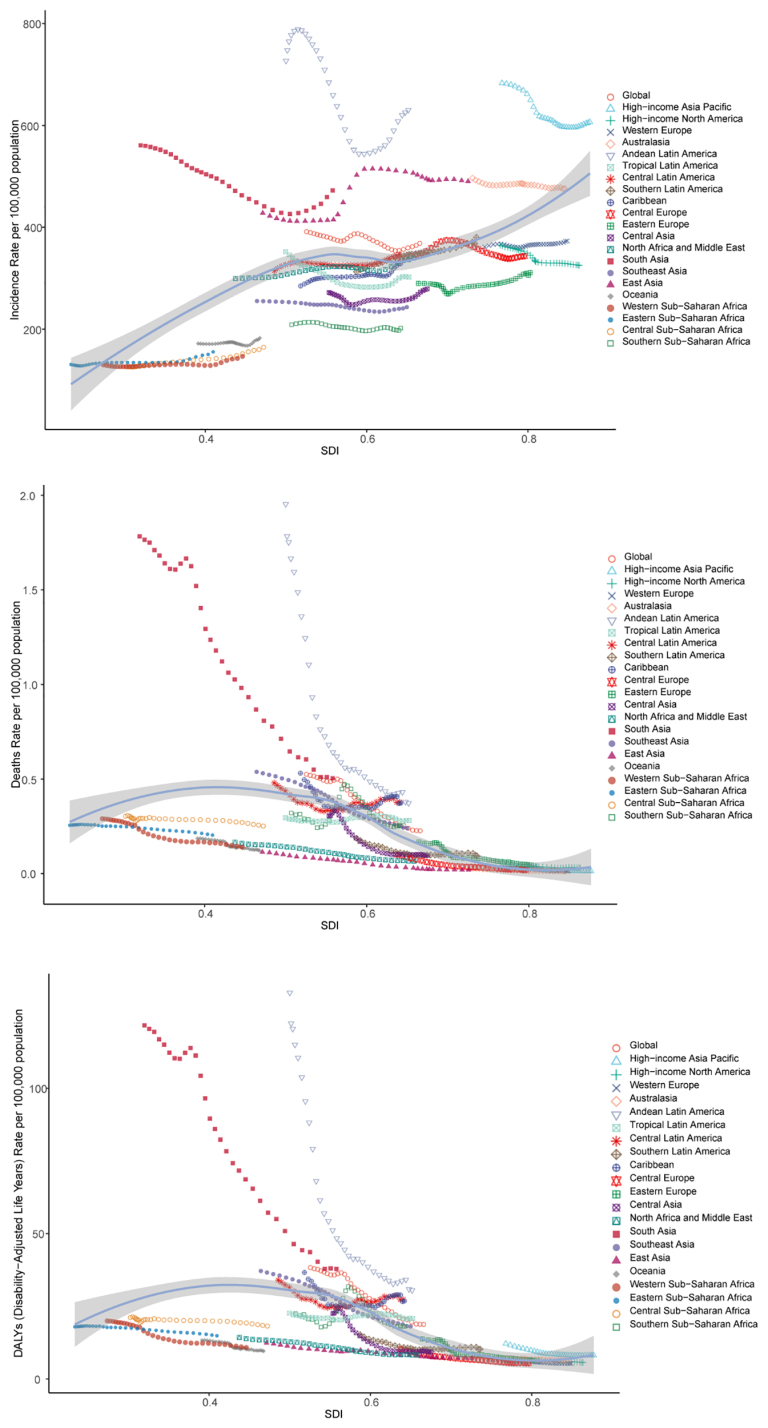
(A) Incidence, (B) death and (C) DALYs rates for appendicitis among AYAs based on SDI from 1990 to 2021. The solid line represents the average relationship between incidence, Death and DALYs rate based on values from all countries from 1990 to 2021. AYAs = adolescents and young adults, DALY = disability-adjusted life years, SDI = socio-demographic index.

The relationship between the burden of appendicitis and SDI levels across 21 geographic regions is illustrated in Figure 4. In 2021, high-middle SDI regions reported the highest incidence rate of appendicitis (417.78; 95% UI: 303.32–577.90 per 100,000) and showed the greatest increasing trend from 1990 to 2021 (EAPC = 0.52; 95% UI: 0.39–0.65). Low SDI regions had the lowest incidence rate in 2021 (247.25; 95% UI: 188.22–322.14 per 100,000) and experienced the largest decrease over the past 3 decades (EAPC = −1.05; 95% UI: −1.19–−0.91). Overall, appendicitis incidence rates remained relatively stable across most regions stratified by SDI levels. Regarding death and DALY rates, most SDI regions showed decreasing trends, with notable reductions in South Asia and Andean Latin America. Higher SDI levels were generally associated with lower death and DALY rates, as shown in Fig S3, Supplemental Digital Content 6, (Supplemental Digital Content).

### 3.5. EAPC correlation factors analysis in 2021

**Figure 5.**
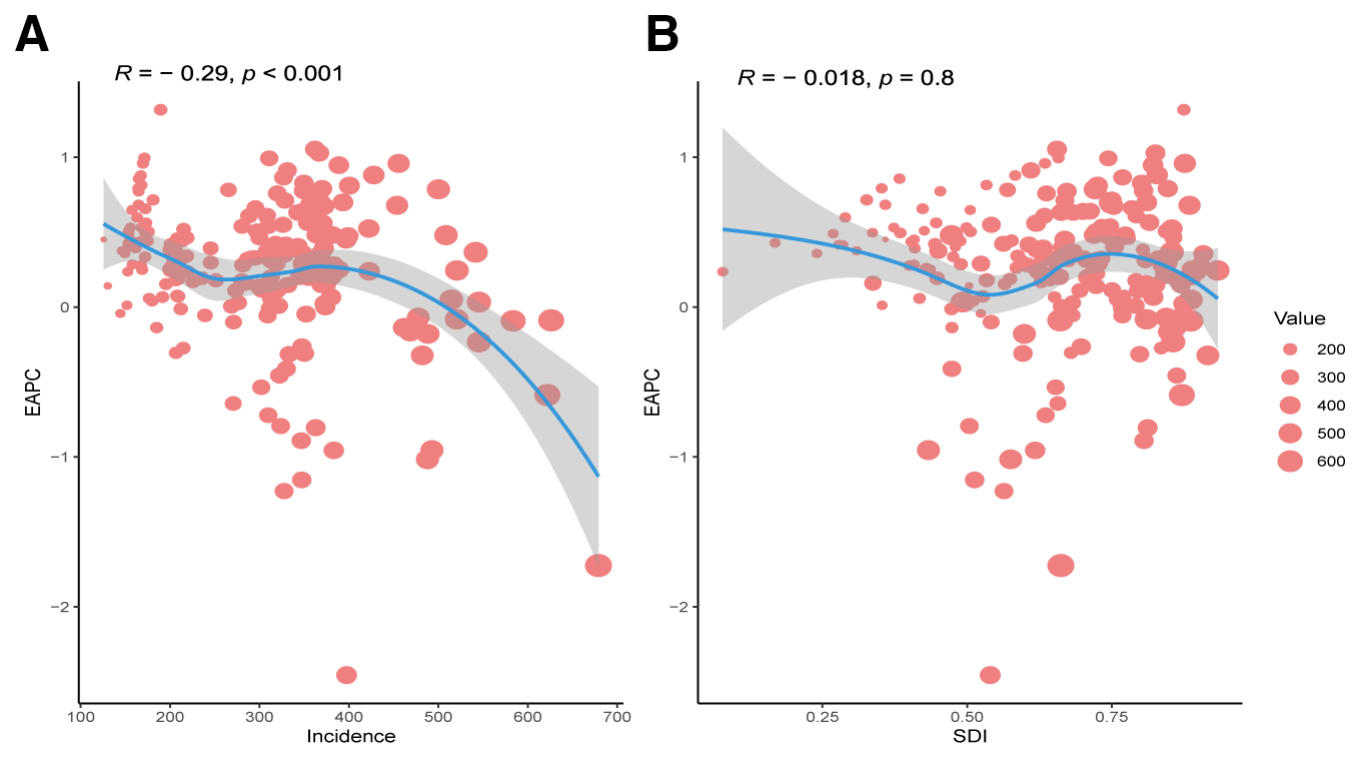
Correlation between the (A) EAPC and appendicitis incidence rate and (B) SDI index in 2021. EAPC = estimated annual percentage changes, SDI = socio-demographic index.

As shown in Figure 5 and Fig S4, Supplemental Digital Content 7, (Supplemental Digital Content), the *R* indices and *P* values were derived from Pearson correlation analysis. A positive correlation was observed between EAPCs and death rates (*R* = 0.41, *P* < .001), while a negative correlation was found between EAPCs in death rates and SDI (*R* = 0.35, *P* < .001). A weak negative correlation was noted between EAPCs and incidence rates (*R* = −0.29, *P* < .001). However, no significant correlation was observed between EAPCs and DALY rates (*R* = 0.09, *P* = .19). Additionally, the EAPCs in incidence and DALY rates showed no significant correlation with SDI.

### 3.6. Prediction of appendicitis incidence and DALYs from 2021 to 2040

**Figure 6.**
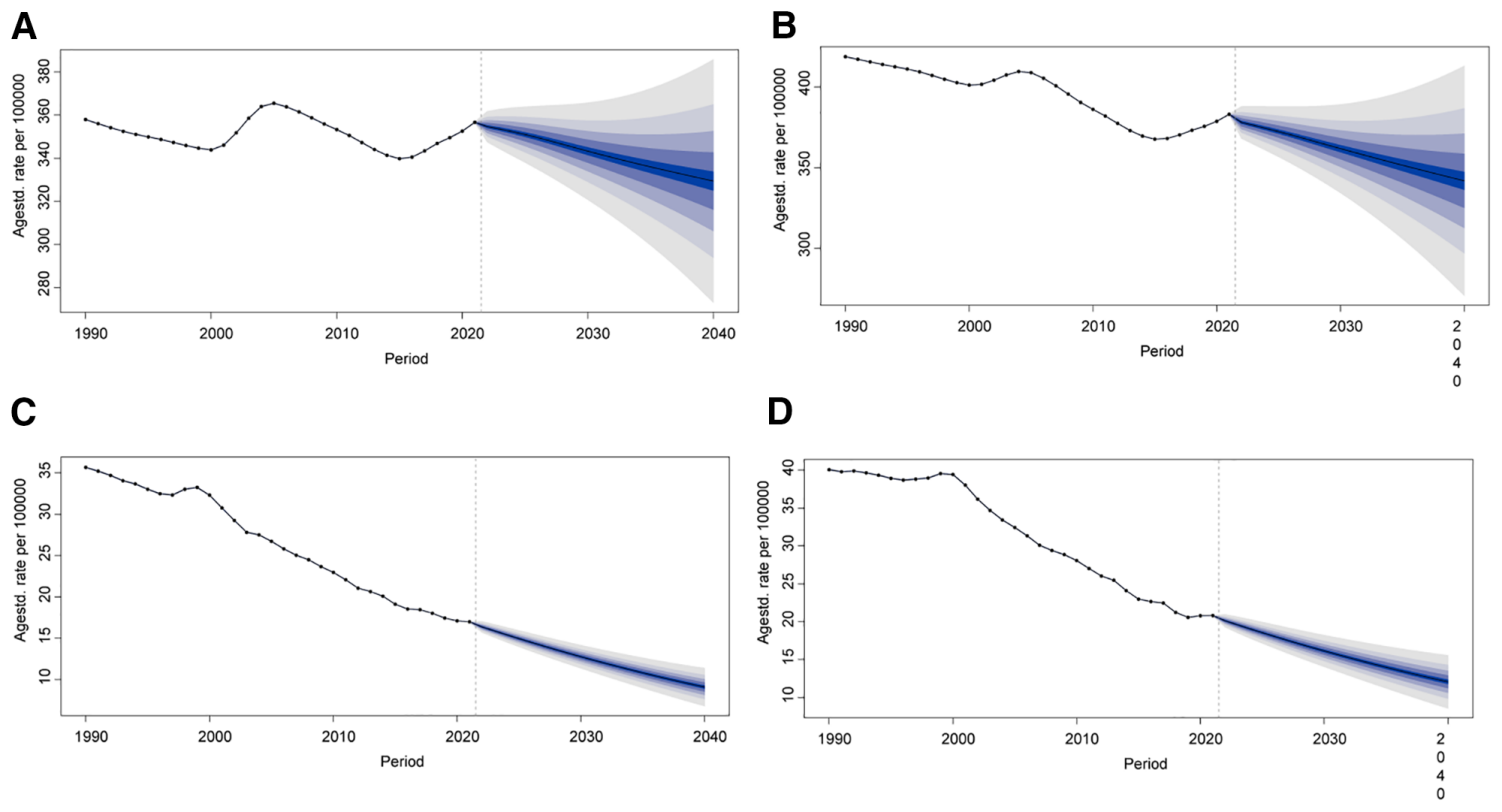
The predictions of global incidence and DALY rate for males and females from 2021 to 2040. (A) Incidence rate for males. (B) Incidence rate for females. (C) DALY rate for males. (D) DALY rate for females. DALY = disability-adjusted life years.

The study projected the incidence and DALY rates of appendicitis among AYAs from 2021 to 2040 using the BAPC model. In these projections, the black line represents the predicted mean prevalence rate, while the gradient area indicates the 95% CI. Both incidence and DALY rates are expected to decline over this period. The global incidence rate for appendicitis in AYAs is predicted to decrease from 369.58 per 100,000 population in 2021 to 334.63 per 100,000 population in 2040. Similarly, the corresponding DALY rates are expected to decline from 18.86 per 100,000 population in 2021 to 10.54 per 100,000 population in 2040. Both incidence and DALY rates for males and females are anticipated to decrease over this time frame (Fig. 6).

## 4. Discussion

This study, based on data from GBD 2021, aimed to assess the burden and trends of appendicitis at global, regional, and national levels among AYAs from 1990 to 2021. Our findings indicated that the incidence rate remained generally stable over the study period, and similar conclusions have been reported in a recent study that analyzed the global burden of appendicitis among children and adolescents aged 0 to 19 years.^[21]^

Analysis of disease burden by gender revealed that females aged 15 to 39 years appeared more susceptible to appendicitis in 2021. This contrasts with other studies that reported higher incidence rates in males within the general population.^[2]^ Further analysis showed that the gender difference in incidence was most pronounced in older adolescents (15–19 years) and young adults (20–24 years), while the difference diminished in the 25 to 39 age group. This shift could be attributed to pregnancy and the use of oral contraceptives among adolescent and young adult females. Hormonal levels and immune system responses likely play a role in this variation, as previous research indicated that appendicitis occurs less frequently during pregnancy.^[22]^ Pregnancy involves changes in hormonal and inflammatory factor levels,^[23]^ and other studies have demonstrated fluctuations in appendicitis incidence during the menstrual cycle.^[24]^ Research has also shown that pregnancy is associated with changes in intestinal microflora,^[25,26]^ which may have a potential connection to the incidence of appendicitis.^[27]^ These findings suggest that global health policies should account for gender differences in appendicitis among AYAs, and targeted strategies to address these differences are warranted.

This study also found that the global incidence rate of appendicitis among AYAs remained generally stable from 1990 to 2021 and was not significantly associated with SDI levels. AYAs consistently exhibited the highest incidence rates, with the peak incidence occurring in the 20 to 24 age group, aligning with previous findings from 2019. The higher incidence in young adults may be related to the number and size of lymphoid follicles, which are believed to play a role in the pathogenesis of appendicitis.^[28]^ Our study also observed that the mean age of patients with appendicitis appears to be increasing. Consistent with other research, the proportion of older individuals with acute appendicitis has risen significantly over the past decade.^[29]^

Despite the substantial burden of appendicitis among AYAs, there is optimism that this burden can be reduced through various measures in the future. Timely diagnosis and treatment are critical in promoting recovery and reducing complications.^[30]^ In addition to traditional diagnostic approaches based on clinical characteristics and laboratory markers, medical imaging plays a significant role in improving diagnostic accuracy. CT, magnetic resonance imaging, and ultrasonography are the primary imaging methods utilized for appendicitis detection.^[31,32]^

The precise cause of appendicitis remains unclear,^[33]^ although obstruction of the appendix is often considered a direct trigger.^[34]^ Research has suggested that genetic polymorphism patterns may significantly influence the pathogenesis of appendicitis.^[35]^ While the exact etiology of appendicitis in AYAs is not fully understood, factors such as dietary habits, environmental influences, and lifestyle choices are likely significant risk factors. In this study, the overall incidence rate of appendicitis among AYAs was observed to increase with rising SDI levels. Dietary habits are associated with the incidence of various diseases, including appendicitis.^[36]^ Countries with higher SDI levels, which often report higher appendicitis incidence rates among AYAs, tend to have diets characterized by frequent consumption of meat and fast food and low intake of fruits and vegetables. Such dietary patterns may elevate the risk of acute appendicitis.^[37]^ Additionally, typical Western dietary habits, marked by low fiber and high sugar intake, have been proposed as contributing factors in the development of appendicitis.^[38]^ Smoking has also been identified as a risk factor for appendectomy.^[39]^ Environmental factors appear to play a role in the incidence of appendicitis as well. Long-term research has shown that higher temperatures from May through July correlate with increased rates of acute appendicitis,^[40]^ and a high rate of complicated acute appendicitis has been observed during both summer and winter months.^[41]^ Changes in the human intestinal microbiome have also been linked to appendicitis,^[42]^ suggesting that the disease’s complexity extends far beyond earlier understandings.

Reducing mortality and disability rates is a key strategy for decreasing the disease burden of appendicitis among AYAs. Perforation is one of the most serious complications of appendicitis, potentially leading to sepsis, peritonitis, and life-threatening conditions. Prompt and effective treatment, combined with proactive measures to prevent complications, can significantly reduce mortality and disability rates.^[3,43]^

Our findings also highlight that lower SDI regions bear a disproportionately higher burden of appendicitis among AYAs. Previous studies have demonstrated a strong link between disease burden and socioeconomic development.^[6]^ The burden of appendicitis varies significantly across regions and countries with differing SDI levels. Patients in economically underdeveloped regions often face limited access to diagnostic tools such as ultrasound and CT scans, which compromises diagnostic accuracy.^[44,45]^ Furthermore, low SDI regions often lack comprehensive healthcare systems, and patients in these areas encounter challenges such as long travel distances to hospitals, lack of transportation, and inadequate surgical facilities.^[46]^ Despite these challenges, low and low-middle SDI regions have made notable progress in reducing the disease burden during the study period. In our analysis, DALY rates decreased substantially in Andean Latin America and South Asia, a trend consistent with findings in the general population.^[6]^

It is important to note that the decline in DALY rates has slowed, decreasing from 18.869 (95% UI: 15.901–22.892) in 2019 to 18.735 (95% UI: 15.593–22.910) in 2021. This slower decline may reflect advancements in medical care, including the establishment of standardized guidelines for diagnosing and treating appendicitis.^[47]^ Over recent years, laparoscopic appendectomy and targeted antibiotic therapies have become recommended treatments for acute appendicitis.^[48]^ The coronavirus disease 2019 pandemic also impacted the management and clinical severity of appendicitis. During the early stages of the pandemic, hospital admissions and surgical emergencies for non-respiratory conditions decreased significantly.^[49]^ Delayed diagnosis and intervention for acute appendicitis during this period often resulted in perforation, leading to worse clinical outcomes and an increased disease burden. These trends are supported by findings from other studies.^[50,51]^

In this study, we forecasted the incidence and DALY rates of appendicitis from 2021 to 2040. The results suggest a significant overall decline in DALY rates, while the global incidence rate of appendicitis among AYAs is expected to decrease slightly. Despite this decline, appendicitis will remain a prevalent abdominal condition among AYAs for an extended period, highlighting the importance of effective treatment measures.

This research represents the first global epidemiological study on appendicitis among AYAs and provides a projection of the disease burden over the next 20 years. However, the study has several limitations. First, the data were not sourced directly from primary records; the GBD database relies on model fitting, and the accuracy of its estimates depends on the availability and quality of the underlying raw data. Second, disease registries from 30 years ago were incomplete, particularly in low-income countries, and age-specific data in certain regions remain scarce. Third, EAPC was calculated using linear regression, which may be misleading when rates exhibit nonlinear trends. Finally, the lack of data on subtypes and pathological classifications of appendicitis in the GBD database limited the scope of our analysis.

In conclusion, our study revealed a significant reduction in the global burden of appendicitis among AYAs from 1990 to 2021. However, the absolute number of deaths remains high, and appendicitis continues to pose a major public health challenge worldwide. Our findings may contribute to the development of more targeted health and medical policies that align with the epidemiological trends of appendicitis in this population.

## Acknowledgments

The authors appreciate the works by Global Burden of Disease Study 2021 collaborators, and JD_GBDR(V2.22, Jingding Medical Technology Co.,Ltd.) was used for drawing of the figures.

## Author contributions

**Data curation:** Ming Yan, Han Wang, Tao Hu, Shun Xu.

**Formal analysis:** Ming Yan, Tao Hu.

**Software:** Han Wang.

**Writing – original draft:** Qian Sun.

**Writing – review & editing:** Xinghao Chen, Shun Xu.


















