# Supplementary material for: Global burden and trends of appendicitis among adolescents and young adults: A systematic analysis for the Global Burden of Disease study 2021 and predictions to 2040
Source: Medicine (Baltimore). 2026 Jul 3;105(27):e49625. doi: 10.1097/MD.0000000000049625 (PMC13336947; doi:10.1097/MD.0000000000049625)
Supplement: Supplementary file 1 [file medi-105-e49625-s001.docx]

S1 Table. Death of appendicitis in AYAs between 1990 to 2021 at the Global, Sex and Regional level, and PC from 1990 to 2021.

| Location/Sex | | 1990 | | | 2021 | | | 1990-2021 | |
| --- | --- | --- | --- | --- | --- | --- | --- | --- | --- |
|  | Death cases (95% UI) | | Death Rate  (95% UI) | Death cases (95% UI) | | Death Rate  (95% UI) | PC  (95% UI) | | EAPC  (95% CI) |
| Sex |  | |  |  | |  |  | |  |
| Male | 5518.20(3881.99-7384.97) | | 0.50(0.35-0.67) | 3070.30(2576.46-3802.39) | | 0.20(0.17-0.25) | -59.15(-67.72--36.26) | | -2.66(-2.83--2.49) |
| Female | 5991.39(3785.75-8368.88) | | 0.55(0.35-0.77) | 3666.40(2638.10-4941.50) | | 0.25(0.18-0.34) | -54.75(-69.26--31.63) | | -2.26(-2.50--2.03) |
| Global | 11509.59(8989.49-15268.25) | | 0.53(0.41-0.70) | 6736.71(5623.45-8112.33) | | 0.23(0.19-0.27) | -56.88(-66.65--42.77) | | -3.01(-3.23--2.78) |
| Socio-demographic index | | | | | | | | | |
| High SDI | 175.66(156.90-189.76) | | 0.05(0.05-0.06) | 74.28(69.31-81.50) | | 0.02(0.02-0.02) | -58.47(-61.93--53.25) | | -2.66(-2.85--2.47) |
| High-middle SDI | 494.36(439.30-554.76) | | 0.11(0.10-0.12) | 153.07(134.97-186.36) | | 0.04(0.03-0.04) | -68.17(-73.01--60.56) | | -3.93(-4.08--3.78) |
| Middle SDI | 2691.61(2143.64-3191.40) | | 0.36(0.29-0.42) | 1590.33(1335.32-1935.40) | | 0.17(0.14-0.21) | -52.05(-60.50--36.38) | | -2.43(-2.48--2.39) |
| Low-middle SDI | 5972.81(4518.93-8268.69) | | 1.32(0.10-1.82) | 3404.89(2752.84-4230.54) | | 0.42(0.34-0.53) | -67.79(-77.61--55.52) | | -3.94(-4.20--3.68) |
| Low SDI | 2169.56(1364.01-3123.84) | | 1.18(0.74-1.70) | 1510.00(1111.10-2081.49) | | 0.34(0.25-0.46) | -71.43(-79.09--60.18) | | -4.50(-4.89--4.11) |
| Regions |  | |  |  | |  |  | |  |
| High-income Asia Pacific | 41.34(28.79-50.22) | | 0.06(0.04-0.07) | 6.63(5.59-8.05) | | 0.01(0.01-0.02) | -78.60(-83.58--68.74) | | -4.33(-4.99--3.66) |
| Central Asia | 86.74(77.67-95.41) | | 0.31(0.27-0.34) | 35.98(27.87-53.81) | | 0.10(0.08-0.14) | -68.44(-75.40--54.15) | | -4.68(-5.30--4.07) |
| East Asia | 646.67(440.02-787.27) | | 0.11(0.08-0.14) | 93.99(72.03-125.62) | | 0.02(0.02-0.03) | -82.84(-88.23--71.77) | | -6.29(-6.63--5.94) |
| South Asia | 7696.80(5742.15-10312.39) | | 1.78(1.33-2.39) | 3992.57(3117.73-5176.13) | | 0.51(0.39-0.65) | -71.69(-80.54--59.69) | | -4.45(-4.81--4.08) |
| Southeast Asia | 1060.71(655.88-1521.93) | | 0.54(0.33-0.77) | 666.53(453.23-834.25) | | 0.24(0.16-0.30) | -55.36(-65.91--37.95) | | -2.77(-2.89--2.66) |
| Australasia | 2.29(1.99-2.62) | | 0.03(0.02-0.03) | 1.41(1.21-1.64) | | 0.01(0.01-0.02) | -52.01(-60.39--41.82) | | -2.43(-2.60--2.25) |
| Caribbean | 78.95(62.41-99.87) | | 0.53(0.42-0.67) | 67.97(46.30-92.61) | | 0.37(0.25-0.51) | -29.69(-47.31--11.74) | | -0.50(-0.92--0.08) |
| Central Europe | 47.46(44.65-50.53) | | 0.10(0.10-0.11) | 7.11(6.42-7.89) | | 0.02(0.02-0.02) | -79.95(-82.07--77.62) | | -4.98(-5.38--4.57) |
| Eastern Europe | 143.27(134.64-153.63) | | 0.17(0.16-0.18) | 30.54(27.98-33.24) | | 0.05(0.04-0.05) | -72.37(-75.50--69.04) | | -4.36(-4.61--4.12) |
| Western Europe | 56.08(53.34-59.23) | | 0.04(0.04-0.04) | 16.97(15.99-18.06) | | 0.01(0.01-0.01) | -66.40(-68.72--63.73) | | -3.46(-3.59--3.33) |
| Andean Latin America | 302.10(245.07-361.44) | | 1.95(1.59-2.34) | 100.90(78.93-133.96) | | 0.37(0.29-0.50) | -80.93(-85.69--72.71) | | -5.31(-5.83--4.79) |
| Central Latin America | 327.18(311.87-343.30) | | 0.48(0.46-0.50) | 382.30(340.79-428.83) | | 0.38(0.34-0.42) | -21.15(-30.62--10.76) | | -0.34(-0.70-0.03) |
| Southern Latin America | 35.72(32.18-39.15) | | 0.19(0.17-0.21) | 24.47(21.0-28.77) | | 0.10(0.08-0.11) | -49.34(-57.80--39.24) | | -1.63(-2.09--1.15) |
| Tropical Latin America | 190.42(178.38-202.68) | | 0.30(0.28-0.32) | 248.430(234.27-264.52) | | 0.28(0.27-0.30) | -4.99(-12.99-3.60) | | 0.12(-0.02-0.27) |
| North Africa and Middle  East | 223.06(143.15-321.79) | | 0.17(0.11-0.24) | 168.19(108.16-230.59) | | 0.07(0.04-0.09) | -60.31(-71.54--42.70) | | -3.19(-3.33--3.06) |
| High-income North  America | 44.12(42.61-45.77) | | 0.04(0.04-0.04) | 37.33(35.17-39.22) | | 0.03(0.03-0.03) | -22.216(-26.26--18.06) | | -0.85(-1.05--0.64) |
| Oceania | 4.94(2.52-11.23) | | 0.19(0.10-0.42) | 6.90(3.99-12.98) | | 0.12(0.07-0.23) | -34.07(-55.77--3.93) | | -1.42(-1.55--1.29) |
| Central Sub-Saharan  Africa | 63.03(30.26-110.05) | | 0.30(0.15-0.53) | 136.08(72.90-247.17) | | 0.25(0.14-0.46) | -17.13(-47.92-47.41) | | -0.43(-0.53--0.33) |
| Eastern Sub-Saharan  Africa | 181.32(89.45-452.79) | | 0.26(0.13-0.64) | 357.13(211.40-863.34) | | 0.20(0.12-0.49) | -20.30(-43.96-26.78) | | -0.72(-0.83--0.61) |
| Southern Sub-Saharan  Africa | 69.32(55.46-109.45) | | 0.32(0.26-0.51) | 85.79(69.30-110.68) | | 0.25(0.20-0.33) | -21.41(-50.49-4.03) | | -0.44(-1.26-0.39) |
| Western Sub-Saharan  Africa | 208.09(119.64-451.31) | | 0.29(0.17-0.63) | 269.47(184.07-412.61) | | 0.14(0.10-0.22) | -51.52(-67.75--28.74) | | -2.59(-2.79--2.40) |

AYAs = adolescents and young adults, PC = percentage change, EAPC = estimated annual percentage changes.
