# Supplementary material for: Global burden and trends of appendicitis among adolescents and young adults: A systematic analysis for the Global Burden of Disease study 2021 and predictions to 2040
Source: Medicine (Baltimore). 2026 Jul 3;105(27):e49625. doi: 10.1097/MD.0000000000049625 (PMC13336947; doi:10.1097/MD.0000000000049625)
Supplement: Supplementary file 2 [file medi-105-e49625-s002.docx]

S2 Table. DALYs of appendicitis in AYAs between 1990 to 2021 at the Global, Sex and Regional level,and PC from 1990 to 2021.

| Location/Sex | | 1990 | | | 2021 | | | 1990-2021 | |
| --- | --- | --- | --- | --- | --- | --- | --- | --- | --- |
|  | DALYs cases (95% UI) | | DALYs Rate  (95% UI) | DALYs cases (95% UI) | | DALYs Rate  (95% UI) | PC  (95% UI) | | EAPC  (95% CI) |
| Sex |  | |  |  | |  |  | |  |
| Male | 397641.38(293925.52-519804.39) | | 35.87(26.52-46.89) | 255988.10(209932.40-309512.09) | | 16.956(13.91-20.50) | -52.733(-61.98--31.20) | | -2.637(-2.79--2.48) |
| Female | 441444.47(295336.95-597941.92) | | 40.75(27.26-55.20) | 301346.68(229114.00-392400.14) | | 20.57(15.64-26.78) | -49.53(-63.76--28.79) | | -2.55(-2.77--2.32) |
| Global | 839085.85(675015.55-1073813.90) | | 38.28(30.80-48.99) | 557334.78(463862.93-681533.79) | | 18.74(15.59-22.91) | -51.06(-60.76--37.47) | | -2.59(-2.78--2.40) |
| Socio-demographic index | | | | | | | | | |
| High SDI | 28479.93(20800.00-38304.28) | | 8.21(6.00-11.04) | 21264.10(14939.10-29249.18) | | 6.02(4.23-8.28) | -26.66(-33.38--20.49) | | -0.92(-0.99--0.84) |
| High-middle SDI | 51264.27(41829.88-63648.68) | | 11.33(9.24-14.07) | 31533.07(21821.21-44321.53) | | 7.16(4.96-10.07) | -36.78(-48.96--24.44) | | -1.51(-1.60--1.42) |
| Middle SDI | 205111.08(165733.92-242219.92) | | 27.25(22.02-32.18) | 141893.85(115858.41-175467.49) | | 15.30(12.49-18.92) | -43.86(-53.12--29.14) | | -1.91(-1.96--1.87) |
| Low-middle SDI | 407601.01(316692.88-557144.95) | | 89.90(69.85-122.88) | 252330.34(207395.30-312322.90) | | 31.44(25.84-38.92) | -65.02(-74.90--52.82) | | -3.70(-3.94--3.47) |
| Low SDI | 146211.97(92477.96-205953.14) | | 79.33(50.18-111.74) | 109978.11(83079.92-146327.29) | | 24.49(18.50-32.59) | -69.13(-76.92--57.30) | | -4.29(-4.66--3.92) |
| Regions |  | |  |  | |  |  | |  |
| High-income Asia Pacific | 8179.38(5676.68-11288.73) | | 12.12(8.41-16.73) | 4101.38(25889.00-5954.80) | | 8.12(5.12-11.78) | -33.03(-44.79--21.17) | | -1.12(-1.36--0.89) |
| Central Asia | 6403.92(5664.87-7187.91) | | 22.51(19.91-25.26) | 3456.40(2716.10-4923.18) | | 9.25(7.27-13.17) | -58.92(-66.93--45.74) | | -3.62(-4.13--3.11) |
| East Asia | 69886.35(52371.60-88625.68) | | 12.35(9.26-15.67) | 34158.74(21742.42-50684.59) | | 7.13(4.54-10.58) | -42.28(-57.70--24.61) | | -1.77(-1.89--1.64) |
| South Asia | 524704.64(400391.50-687453.13) | | 121.57(92.77-159.27) | 299146.58(239230.25-379842.72) | | 37.82(30.25-48.03) | -68.89(-77.81--56.56) | | -4.18(-4.51--3.85) |
| Southeast Asia | 73134.63(48200.61-100439.64) | | 37.12(24.47-50.98) | 50005.09(36037.73-62169.01) | | 18.03(13.00-22.42) | -51.43(-62.21--35.03) | | -2.50(-2.59--2.40) |
| Australasia | 613.01(393.76-917.43) | | 7.52(4.83-11.25) | 673.43(394.30-1045.10) | | 6.43(3.77-9.98) | -14.45(-36.81-13.64) | | -0.48(-0.52--0.43) |
| Caribbean | 5441.13(4395.92-6729.33) | | 36.61(29.57-45.27) | 4879.14(3511.33-6358.60) | | 26.80(19.29-34.93) | -26.77(-42.24--10.88) | | -0.44(-0.81--0.06) |
| Central Europe | 4797.48(3992.49-5873.75) | | 10.24(8.52-12.54) | 1892.44(1329.99-2548.77) | | 5.40(3.80-7.28) | -47.23(-56.65--38.60) | | -2.01(-2.18--1.83) |
| Eastern Europe | 11803.96(10372.02-13773.52) | | 13.76(12.09-16.06) | 4235.50(3202.82-5745.11) | | 6.40(4.84-8.68) | -53.49(-61.62--44.68) | | -2.64(-2.87--2.40) |
| Western Europe | 9719.55(7014.56-13149.31) | | 6.74(4.87-9.12) | 6870.92(4490.85-9970.49) | | 5.30(3.46-7.68) | -21.49(-32.31--9.71) | | -0.78(-0.84--0.73) |
| Andean Latin America | 20536.76(16792.73-24534.67) | | 132.81(108.60-158.66) | 8256.08(6657.83-10526.01) | | 30.49(24.59-38.87) | -77.04(-82.51--68.29) | | -4.82(-5.31--4.32) |
| Central Latin America | 23198.79(21828.48-24829.11) | | 33.98(31.98-36.37) | 27624.30(24528.60-31444.98) | | 27.31(24.25-31.08) | -19.64(-28.54--10.43) | | -0.35(-0.67--0.03) |
| Southern Latin America | 2892.24(2490.19-3429.36) | | 15.160(13.05-17.97) | 2640.50(2055.26-3405.32) | | 10.24(7.97-13.20) | -32.48(-45.41--17.99) | | -0.92(-1.23--0.61) |
| Tropical Latin America | 14537.26(13285.37-16100.56) | | 22.60(20.66-25.04) | 18333.09(16732.35-20142.94) | | 20.76(18.95-22.81) | -8.16(-14.80--1.39) | | -0.02(-0.14-0.10) |
| North Africa and Middle  East | 19077.86(13572.83-26183.58) | | 14.26(10.14-19.57) | 20876.87(15048.37-28017.46) | | 8.21(5.92-11.02) | -42.40(-56.82--21.91) | | -1.98(-2.07--1.89) |
| High-income North  America | 7555.01(5585.81-10022.27) | | 6.67(4.93-8.85) | 6964.25(5315.64-8802.56) | | 5.65(4.32-7.15) | -15.20(-27.05--3.65) | | -0.56(-0.62--0.49) |
| Oceania | 353.18(207.46-719.57) | | 13.30(7.81-27.09) | 539.60(362.57-917.58) | | 9.58(6.44-16.29) | -27.97(-50.01--1.70) | | -1.15(-1.27--1.040) |
| Central Sub-Saharan  Africa | 4384.04(2273.35-7392.18) | | 21.12(10.95-35.60) | 9822.97(5841.69-16735.79) | | 18.16(10.80-30.94) | -14.01(-44.02-43.67) | | -0.34(-0.42--0.25) |
| Eastern Sub-Saharan  Africa | 12707.38(6786.57-30111.49) | | 17.93(9.57-42.48) | 26024.51(16654.32-57838.26) | | 14.86(9.51-33.02) | -17.13(-39.21-26.42) | | -0.63(-0.72--0.54) |
| Southern Sub-Saharan  Africa | 4855.38(3931.80-7395.57) | | 22.46(18.19-34.21) | 6151.30(5053.15-7729.70) | | 18.07(14.85-22.71) | -19.54(-47.17-2.63) | | -0.42(-1.14-0.31) |
| Western Sub-Saharan  Africa | 14303.88(8954.69-29317.48) | | 19.99(12.51-40.96) | 20681.73(14945.49-29944.82) | | 10.82(7.82-15.66) | -45.88(-63.12--22.60) | | -2.25(-2.43--2.07) |

AYAs = adolescents and young adults, PC = percentage change, EAPC = estimated annual percentage changes.
