# Supplementary figures and images for: Global burden and trends of appendicitis among adolescents and young adults: A systematic analysis for the Global Burden of Disease study 2021 and predictions to 2040
Source: Medicine (Baltimore). 2026 Jul 3;105(27):e49625. doi: 10.1097/MD.0000000000049625 (PMC13336947; doi:10.1097/MD.0000000000049625)

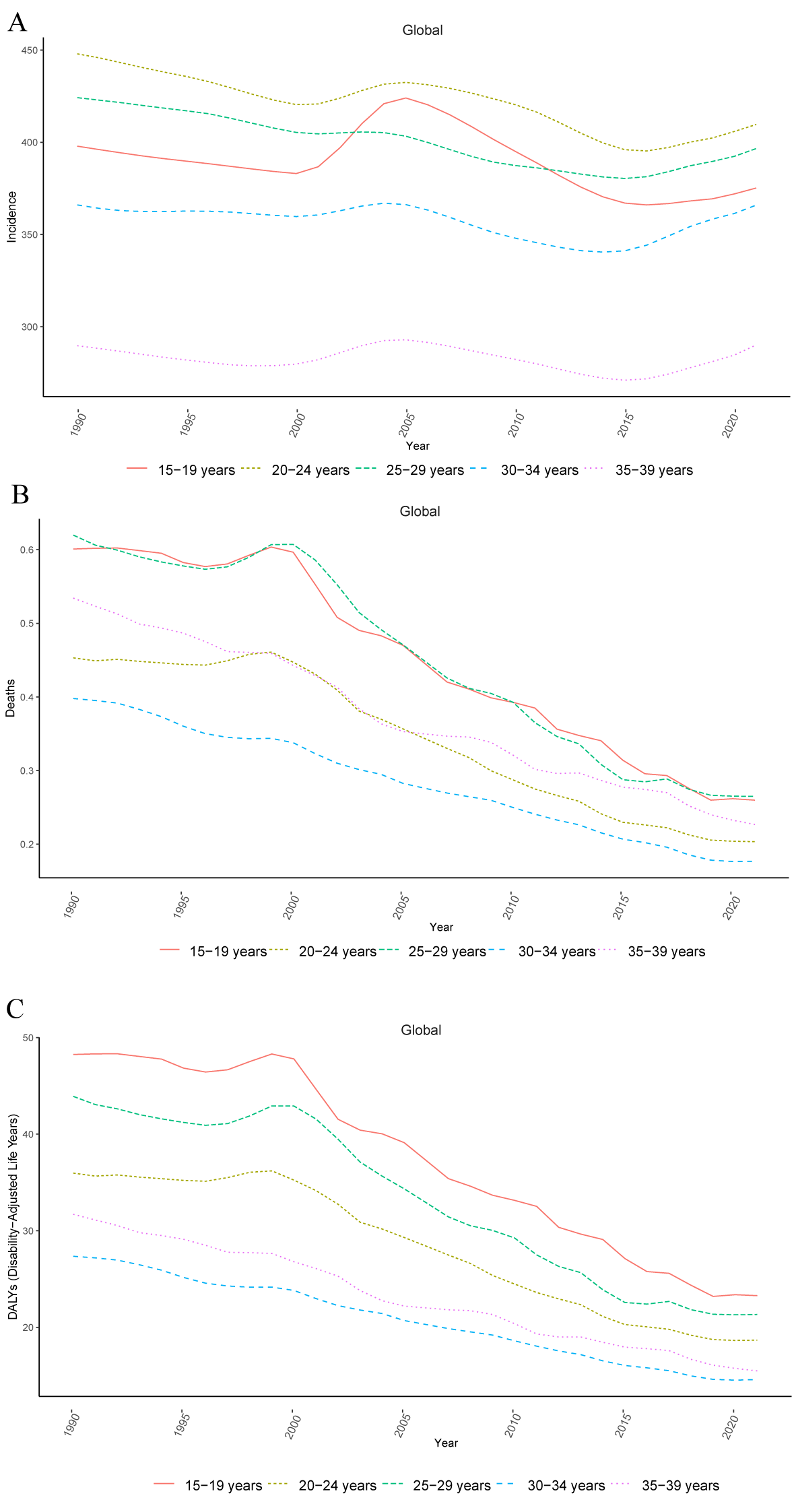

Supplement: Supplementary file 3 [file medi-105-e49625-s003.tif]

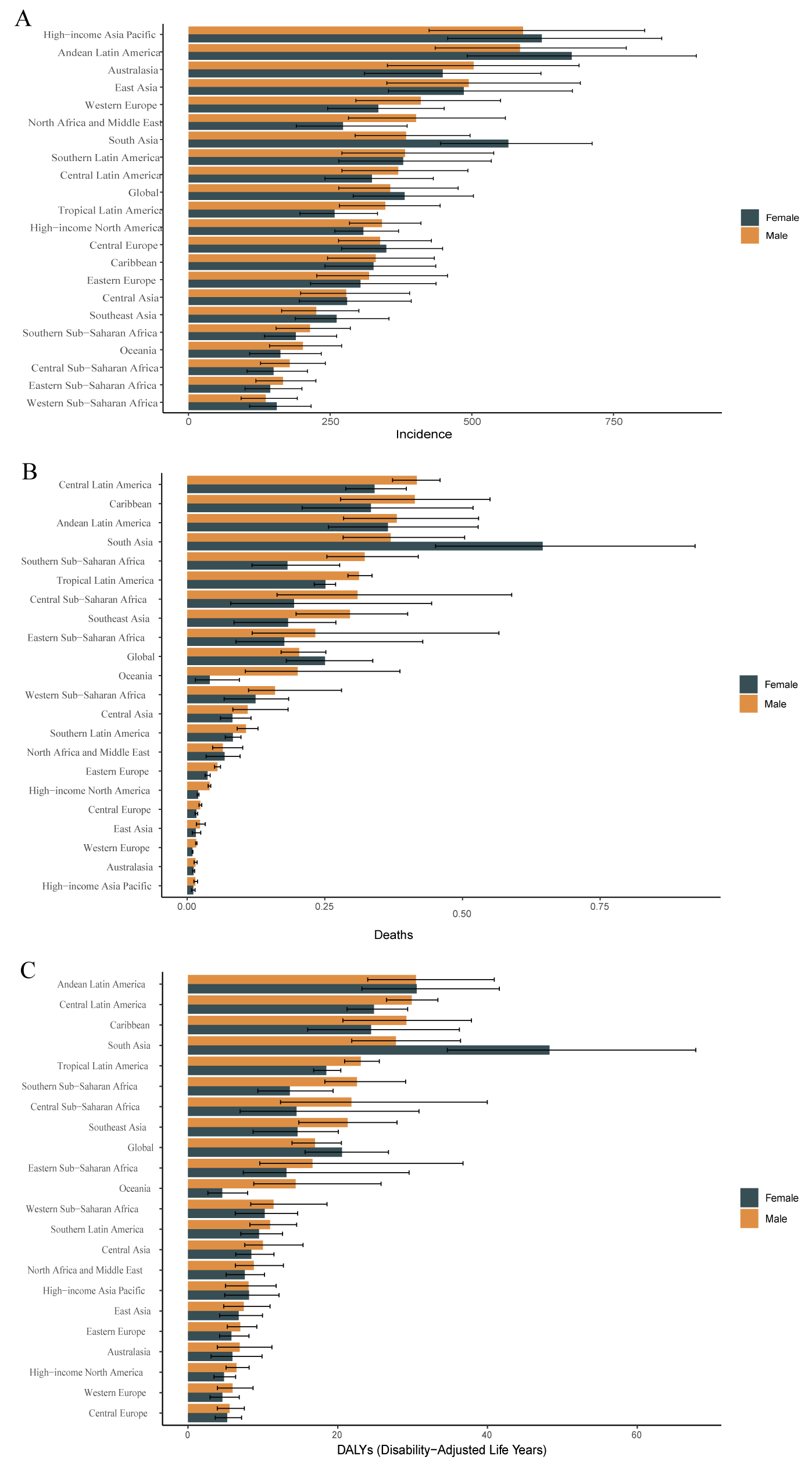

Supplement: Supplementary file 4 [file medi-105-e49625-s004.tif]

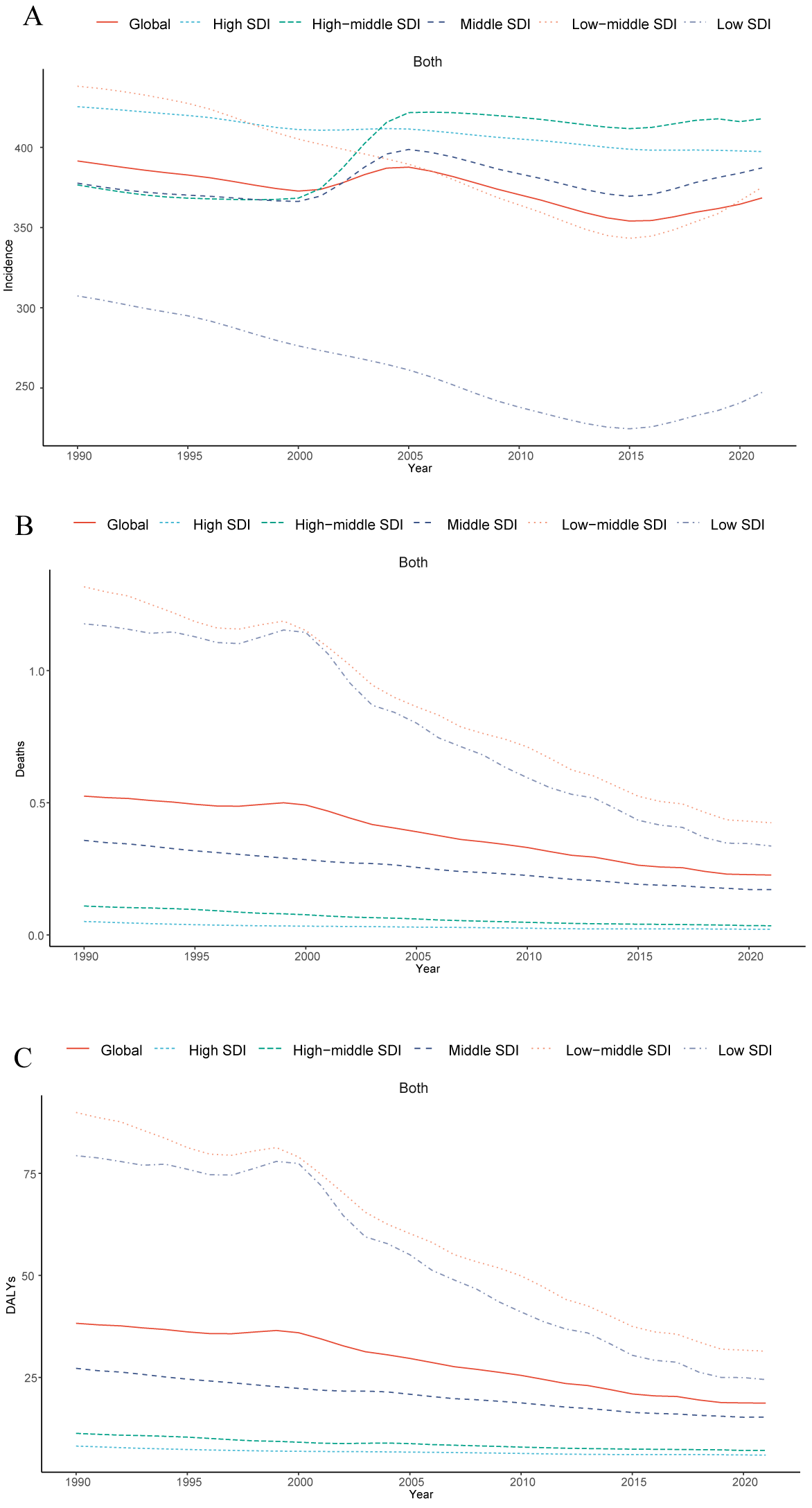

Supplement: Supplementary file 6 [file medi-105-e49625-s006.tif]

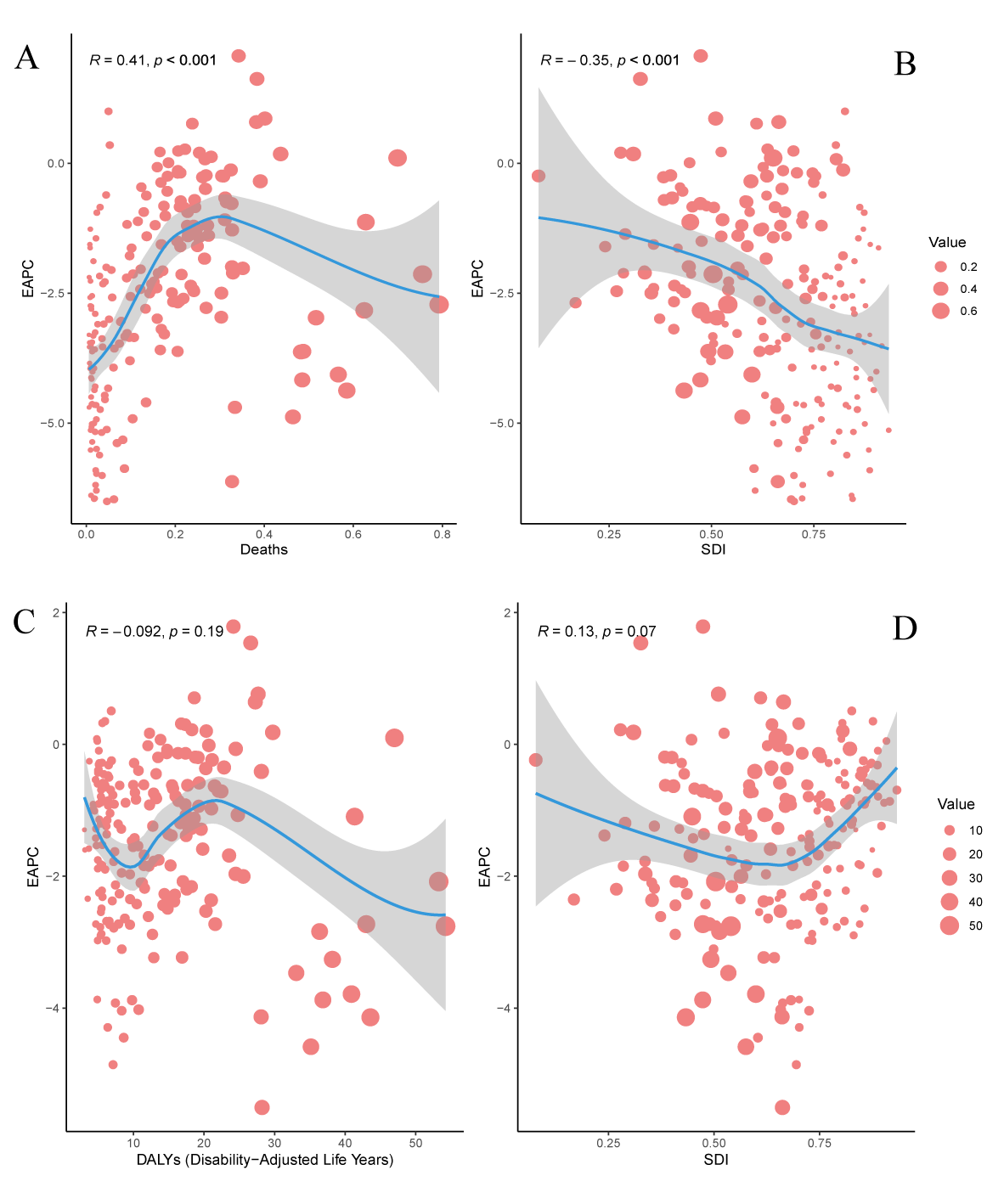

Supplement: Supplementary file 7 [file medi-105-e49625-s007.tif]
