# Supplementary material for: Global burden and trends of appendicitis among adolescents and young adults: A systematic analysis for the Global Burden of Disease study 2021 and predictions to 2040
Source: Medicine (Baltimore). 2026 Jul 3;105(27):e49625. doi: 10.1097/MD.0000000000049625 (PMC13336947; doi:10.1097/MD.0000000000049625)
Supplement: Supplementary file 5 [file medi-105-e49625-s005.docx]

S3 Table. Incidence burden for appendicitis among AYAs by 204 countries or territories in 1990 and 2021, and PC from 1990 to 2021.

| Location | 1990 | | 2021 | |  |  |
| --- | --- | --- | --- | --- | --- | --- |
|  | Incidence case (95%UI) | Incidence rate (95%UI) | Incidence case (95%UI) | Incidence rate (95%UI) | Percentage change(95%UI) | EAPC |
| American Samoa | 36.15(24.80,51.26) | 178.68(122.60,253.35) | 37.59(26.79-52.08) | 215.11(153.30,298.01) | 20.39(11.63,32.12) | 0.52(0.48,0.56) |
| Antigua and Barbuda | 74.73(52.99,102.46) | 289.79(205.48,397.29) | 126.22(89.82,175.42) | 366.94(261.10,509.96) | 26.62(17.72,36.22) | 0.71(0.67,0.76) |
| Arab Republic of Egypt | 61246.35(41738.09,86801.81) | 279.38(190.39,395.95) | 139037.85(95567.03,197584.44) | 329.38(226.40,468.08) | 17.90(9.62,26.89) | 0.42(0.33,0.50) |
| Argentine Republic | 37148.95(25913.37,52036.60) | 304.12(212.14,426.00) | 65329.92(46234.52,91876.51) | 372.83(263.86,524.33) | 22.59(14.81,33.41) | 0.50(0.45,0.56) |
| Australia | 33240.71(22450.19,46953.58) | 490.83(331.49,693.31) | 41399.76(28469.50,57260.64) | 477.53(328.39,660.48) | -2.71(-8.61,3.40) | -0.07(-0.09,-0.05) |
| Barbados | 307.46(217.10,427.77) | 281.69(198.90,391.91) | 351.78(246.85,482.34) | 356.13(249.90,488.31) | 26.43(17.08,34.71) | 0.68(0.64,0.72) |
| Belize | 185.34(130.91,254.18) | 253.30(178.91,347.39) | 625.23(453.74,839.11) | 331.17(240.33,444.46) | 30.74(21.19,41.85) | 0.91(0.85,0.97) |
| Bermuda | 73.02(50.50,106.22) | 283.94(196.38,413.06) | 68.06(47.65,97.19) | 388.69(272.15,555.09) | 36.89(27.58,47.76) | 0.95(0.91,0.98) |
| Bolivarian Republic of Venezuela | 28691.43(21684.83,38050.43) | 358.23(270.75,475.09) | 32777.31(23858.17,44958.07) | 350.08(254.82,480.18) | -2.27(-10.19,5.38) | -0.31(-0.56,-0.06) |
| Bosnia and Herzegovina | 5577.28(3838.29,7864.51) | 293.68(202.11,414.12) | 3008.80(2084.36,4249.72) | 299.00(207.14,422.32) | 1.81(-3.89,7.72) | 0.14(0.06,0.21) |
| Brunei Darussalam | 663.87(451.67,925.30) | 538.47(366.35,750.52) | 1061.77(725.97,1466.19) | 520.37(355.80,718.58) | -3.36(-9.59,4.04) | -0.08(-0.14,-0.02) |
| Burkina Faso | 4239.05(2942.07,5996.39) | 132.97(92.29,188.09) | 13257.33(9304.10,18379.59) | 153.18(107.51,212.37) | 15.20(7.10,25.85) | 0.42(0.34,0.49) |
| Canada | 40033.04(27796.79,56508.20) | 360.12(250.04,508.32) | 41768.75(28702.13,58820.99) | 352.13(241.98,495.89) | -2.22(-7.51,3.62) | -0.05(-0.07,-0.03) |
| Central African Republic | 1350.53(999.63,1783.62) | 129.69(95.99,171.28) | 3189.68(2384.27,4221.29) | 146.10(109.21,193.35) | 12.65(3.66,21.10) | 0.38(0.32,0.43) |
| Commonwealth of Dominica | 74.64(53.29,102.33) | 255.58(182.48,350.37) | 80.21(57.52,109.55) | 309.71(222.10,422.99) | 21.18(11.69,30.90) | 0.54(0.48,0.60) |
| Commonwealth of the Bahamas | 323.53(230.50,441.44) | 274.15(195.31,374.05) | 540.17(385.55,733.42) | 349.25(249.29,474.21) | 27.40(19.55,36.67) | 0.78(0.72,0.83) |
| Cook Islands | 13.54(9.25,19.13) | 175.49(119.91,247.92) | 12.26(8.46,17.66) | 208.11(143.62,299.70) | 18.59(10.45,28.62) | 0.46(0.42,0.49) |
| Czech Republic | 11397.48(7967.03,16223.56) | 307.16(214.71,437.22) | 12631.96(9581.52,15388.83) | 427.76(324.46,521.11) | 39.26(12.55,78.14) | 0.88(0.50,1.26) |
| Democratic People's Republic of Korea | 17722.45(12306.80,24856.75) | 212.48(147.55,298.01) | 26677.44(18627.68,37307.89) | 265.23(185.20,370.91) | 24.83(16.17,34.06) | 0.78(0.72,0.85) |
| Democratic Republic of Sao Tome and Principe | 55.18(36.44,80.10) | 128.55(84.90,186.60) | 143.07(97.67,200.86) | 157.41(107.46,221.00) | 22.45(13.71,32.53) | 0.65(0.56,0.74) |
| Democratic Republic of the Congo | 17897.18(12760.67,24792.85) | 124.93(89.08,173.07) | 59522.64(41425.32,81779.90) | 164.95(114.80,226.63) | 32.03(23.01,40.93) | 0.86(0.75,0.96) |
| Democratic Republic of Timor-Leste | 561.85(400.47,761.08) | 176.52(125.82,239.11) | 1147.07(800.09,1596.75) | 200.77(140.04,279.48) | 13.74(5.36,22.34) | 0.42(0.37,0.47) |
| Democratic Socialist Republic of Sri Lanka | 13945.94(9701.48,19915.19) | 188.60(131.20,269.32) | 17424.29(12169.37,24735.19) | 216.06(150.90,306.71) | 14.56(6.42,21.63) | 0.33(0.28,0.39) |
| Dominican Republic | 9061.32(6658.33,12232.24) | 294.96(216.74,398.18) | 16852.54(12472.08,22394.74) | 370.61(274.28,492.49) | 25.64(17.42,33.48) | 0.56(0.49,0.63) |
| Eastern Republic of Uruguay | 3583.21(2499.45,5099.80) | 315.47(220.06,449.00) | 4566.31(3193.97,6423.07) | 381.72(267.00,536.94) | 21.00(13.37,30.84) | 0.48(0.42,0.54) |
| Federal Democratic Republic of Ethiopia | 18968.28(13721.76,25556.64) | 103.82(75.11,139.89) | 58261.09(40238.17,80713.74) | 125.69(86.80,174.12) | 21.06(12.48,30.01) | 0.45(0.34,0.56) |
| Federal Democratic Republic of Nepal | 39329.78(33209.52,46352.18) | 538.39(454.61,634.52) | 66076.87(49890.54,85765.88) | 492.83(372.11,639.68) | -8.46(-21.88,5.45) | -0.95(-1.19,-0.72) |
| Federal Republic of Germany | 103259.75(70412.13,144815.12) | 347.58(237.01,487.46) | 93606.16(63964.06,131546.23) | 370.01(252.84,519.98) | 6.45(-0.51,12.43) | 0.28(0.23,0.33) |
| Federal Republic of Nigeria | 41148.42(28827.75,57654.67) | 120.51(84.43,168.86) | 117196.46(79804.40,165138.84) | 130.31(88.73,183.61) | 8.13(4.53,11.56) | 0.14(0.02,0.27) |
| Federal Republic of Somalia | 3965.77(2885.79,5368.94) | 136.79(99.54,185.18) | 12657.08(9253.03,17383.46) | 152.67(111.61,209.67) | 11.61(2.50,21.15) | 0.24(0.15,0.32) |
| Federated States of Micronesia | 77.27(56.41,101.25) | 192.86(140.79,252.72) | 92.79(66.83,122.22) | 218.49(157.38,287.81) | 13.29(6.47,21.72) | 0.34(0.28,0.40) |
| Federative Republic of Brazil | 220616.30(168710.50,280454.32) | 351.60(268.88,446.97) | 257429.30(196954.63,330258.16) | 301.97(231.03,387.40) | -14.12(-17.72,-10.09) | -0.53(-0.70,-0.37) |
| French Republic | 78023.99(52596.31,110922.29) | 354.64(239.07,504.18) | 76094.16(51502.12,107886.50) | 383.03(259.24,543.05) | 8.00(0.73,14.81) | 0.28(0.25,0.31) |
| Gabonese Republic | 487.59(339.74,677.17) | 126.77(88.33,176.06) | 1271.61(886.58,1757.00) | 169.65(118.28,234.41) | 33.83(25.41,43.27) | 0.96(0.87,1.05) |
| Georgia | 5101.24(3516.17,7285.81) | 239.61(165.16,342.22) | 3183.44(2262.13,4477.07) | 280.56(199.36,394.56) | 17.09(7.20,27.19) | 0.54(0.47,0.61) |
| Grand Duchy of Luxembourg | 553.35(381.02,776.38) | 374.97(258.19,526.10) | 1001.13(853.54,1149.31) | 453.81(386.91,520.98) | 21.03(-7.69,65.16) | 0.68(0.50,0.86) |
| Greenland | 87.70(58.95,124.47) | 331.35(222.71,470.24) | 67.64(46.98,96.69) | 331.60(230.30,474.01) | 0.07(-6.56,8.48) | 0.15(0.08,0.21) |
| Grenada | 84.56(60.22,114.36) | 253.62(180.63,343.02) | 132.69(92.16,184.84) | 328.30(228.02,457.30) | 29.45(21.29,39.83) | 0.71(0.64,0.79) |
| Guam | 109.56(74.56,155.94) | 172.73(117.55,245.85) | 110.53(75.89,157.64) | 199.38(136.88,284.36) | 15.43(8.47,23.90) | 0.38(0.33,0.44) |
| Hashemite Kingdom of Jordan | 4936.83(3332.96,6987.39) | 321.21(216.86,454.63) | 21498.55(15068.79,29411.77) | 400.48(280.70,547.89) | 24.68(13.75,38.00) | 0.81(0.73,0.89) |
| Hellenic Republic | 9835.07(6538.74,13724.41) | 261.62(173.93,365.08) | 7804.19(5392.41,11013.01) | 280.34(193.70,395.61) | 7.16(-1.48,16.99) | 0.18(0.12,0.24) |
| Hungary | 11167.55(7911.49,15918.45) | 302.16(214.06,430.70) | 8846.89(6138.73,12580.62) | 321.53(223.10,457.22) | 6.41(-0.92,13.30) | 0.01(-0.06,0.07) |
| Independent State of Papua New Guinea | 2754.50(1891.48,3793.87) | 166.42(114.28,229.21) | 7557.46(5157.88,10540.46) | 176.60(120.52,246.30) | 6.12(-0.91,12.85) | 0.06(-0.02,0.13) |
| Independent State of Samoa | 117.41(80.86,164.59) | 175.34(120.75,245.79) | 163.77(115.30,227.25) | 203.83(143.50,282.83) | 16.25(7.81,25.54) | 0.31(0.24,0.38) |
| Ireland | 4942.40(3277.05,7032.33) | 360.30(238.90,512.66) | 5833.13(3916.04,8243.47) | 372.79(250.27,526.84) | 3.47(-2.23,10.16) | 0.02(-0.02,0.07) |
| Islamic Republic of Afghanistan | 8419.83(6229.65,10953.92) | 267.33(197.80,347.79) | 34746.38(24792.96,46112.43) | 284.32(202.87,377.32) | 6.35(-3.58,17.37) | 0.16(-0.03,0.35) |
| Islamic Republic of Iran | 76229.25(53189.42,106081.00) | 350.99(244.91,488.44) | 120565.99(85018.99,170318.81) | 347.42(244.99,490.79) | -1.02(-10.51,9.66) | -0.26(-0.40,-0.13) |
| Islamic Republic of Mauritania | 1047.39(731.26,1472.52) | 136.23(95.11,191.53) | 2874.94(1931.02,4085.63) | 168.42(113.12,239.34) | 23.62(13.61,32.66) | 0.53(0.44,0.62) |
| Islamic Republic of Pakistan | 161287.26(125204.10,203224.44) | 395.26(306.84,498.04) | 320090.25(251610.32,400167.37) | 323.63(254.39,404.59) | -18.12(-22.25,-13.58) | -0.79(-0.90,-0.69) |
| Jamaica | 2742.52(1918.99,3776.74) | 279.06(195.27,384.30) | 4092.23(2833.62,5624.63) | 342.95(237.47,471.37) | 22.89(13.08,32.83) | 0.63(0.58,0.69) |
| Japan | 327330.47(226484.34,456754.83) | 730.41(505.38,1019.21) | 201553.70(151096.17,265593.41) | 621.88(466.20,819.48) | -14.86(-22.40,-5.42) | -0.59(-0.73,-0.44) |
| Kingdom of Bahrain | 779.87(534.67,1094.14) | 304.23(208.58,426.83) | 2620.07(1779.71,3766.47) | 372.40(252.96,535.34) | 22.41(13.62,31.73) | 0.67(0.61,0.74) |
| Kingdom of Belgium | 16420.22(11096.55,23123.64) | 441.33(298.25,621.50) | 18972.95(16353.47,21887.25) | 541.70(466.91,624.90) | 22.74(-3.68,63.60) | 0.37(0.20,0.53) |
| Kingdom of Bhutan | 1099.76(884.75,1374.60) | 408.14(328.34,510.13) | 1762.39(1314.55,2330.86) | 508.49(379.28,672.51) | 24.59(9.85,40.65) | 0.48(0.35,0.61) |
| Kingdom of Cambodia | 13963.82(11501.57,16920.18) | 362.67(298.72,439.45) | 23908.36(18504.57,30328.95) | 330.08(255.48,418.73) | -8.99(-19.31,0.71) | -0.41(-0.50,-0.33) |
| Kingdom of Denmark | 7547.46(5115.36,10798.01) | 395.59(268.11,565.96) | 7705.47(5208.75,10809.49) | 422.53(285.62,592.74) | 6.81(0.99,12.73) | 0.24(0.08,0.40) |
| Kingdom of Eswatini | 549.94(394.24,749.08) | 182.48(130.82,248.56) | 1033.59(741.33,1389.48) | 203.01(145.61,272.91) | 11.25(3.55,20.04) | 0.26(0.13,0.40) |
| Kingdom of Lesotho | 917.88(659.59,1249.75) | 170.11(122.24,231.62) | 1496.46(1084.53,2012.09) | 179.90(130.38,241.89) | 5.75(-1.30,13.22) | 0.04(-0.10,0.18) |
| Kingdom of Morocco | 28486.03(19491.76,39560.76) | 273.99(187.48,380.51) | 46668.03(32238.99,65560.61) | 317.87(219.59,446.56) | 16.02(8.59,23.86) | 0.42(0.35,0.49) |
| Kingdom of Norway | 8672.21(5991.39,12206.24) | 542.08(374.51,762.98) | 8553.34(6281.52,11545.14) | 481.94(353.94,650.52) | -11.09(-19.27,-2.31) | -0.32(-0.39,-0.25) |
| Kingdom of Saudi Arabia | 19913.14(13621.75,28101.28) | 299.66(204.98,422.88) | 65557.03(45725.18,93436.07) | 353.94(246.87,504.45) | 18.11(5.68,31.36) | 0.56(0.52,0.61) |
| Kingdom of Spain | 53604.68(35985.00,76572.20) | 361.47(242.65,516.34) | 46382.52(32117.26,65809.96) | 373.57(258.67,530.04) | 3.35(-5.31,11.87) | 0.00(-0.04,0.05) |
| Kingdom of Sweden | 15142.44(10251.65,21220.48) | 515.85(349.24,722.91) | 16675.24(11725.22,23447.50) | 514.43(361.72,723.36) | -0.27(-7.20,6.30) | 0.05(-0.01,0.11) |
| Kingdom of Thailand | 59528.91(41944.02,81708.18) | 229.56(161.75,315.09) | 50654.94(35155.77,70149.70) | 238.94(165.83,330.90) | 4.09(-3.42,12.93) | -0.06(-0.10,-0.01) |
| Kingdom of the Netherlands | 22209.18(15073.27,31110.20) | 368.32(249.98,515.94) | 20529.47(13896.70,28705.19) | 388.79(263.17,543.62) | 5.56(-0.43,13.19) | 0.25(0.20,0.30) |
| Kingdom of Tonga | 66.28(45.63,92.72) | 179.65(123.67,251.30) | 79.81(55.29,110.34) | 205.18(142.14,283.66) | 14.21(7.77,21.45) | 0.30(0.26,0.35) |
| Kyrgyz Republic | 4654.14(3297.69,6488.78) | 258.04(182.84,359.76) | 7655.51(5261.17,10738.04) | 281.29(193.32,394.56) | 9.01(-0.05,17.42) | 0.28(0.13,0.44) |
| Lao People's Democratic Republic | 2808.73(2087.80,3719.51) | 181.79(135.13,240.74) | 6503.06(4663.22,8830.98) | 202.72(145.37,275.29) | 11.51(2.00,20.29) | 0.29(0.22,0.35) |
| Lebanese Republic | 3421.99(2315.91,4909.16) | 296.85(200.90,425.86) | 8415.57(5839.97,12023.16) | 362.69(251.69,518.16) | 22.18(10.78,36.53) | 0.70(0.68,0.72) |
| Malaysia | 13996.43(9453.52,19603.25) | 188.58(127.37,264.13) | 30443.80(21015.09,42553.88) | 218.98(151.16,306.09) | 16.12(6.87,25.64) | 0.46(0.43,0.50) |
| Mongolia | 4368.23(3624.21,5314.80) | 494.32(410.13,601.44) | 4834.47(3662.02,6284.91) | 383.04(290.15,497.96) | -22.51(-33.14,-9.10) | -0.96(-1.12,-0.79) |
| Montenegro | 776.44(531.50,1097.45) | 309.31(211.73,437.19) | 657.25(451.82,940.07) | 319.48(219.62,456.95) | 3.29(-3.30,9.59) | 0.11(0.08,0.15) |
| New Zealand | 7239.97(5171.55,9778.15) | 524.08(374.35,707.81) | 8439.72(6391.06,10982.44) | 468.51(354.78,609.66) | -10.60(-17.37,-2.71) | -0.16(-0.25,-0.08) |
| North Macedonia | 2353.89(1603.28,3295.47) | 296.42(201.90,414.99) | 2312.57(1604.77,3339.19) | 302.39(209.84,436.63) | 2.01(-5.71,10.33) | 0.11(0.06,0.17) |
| Northern Mariana Islands | 43.76(30.54,62.50) | 186.87(130.41,266.88) | 34.30(24.20,48.92) | 208.04(146.79,296.72) | 11.33(3.41,19.40) | 0.08(-0.03,0.18) |
| Palestine | 2491.45(1680.63,3495.54) | 324.62(218.98,455.45) | 8122.65(5535.32,11709.32) | 371.98(253.49,536.23) | 14.59(6.51,23.74) | 0.39(0.32,0.47) |
| People's Democratic Republic of Algeria | 31682.39(21759.56,44862.91) | 313.55(215.35,443.99) | 58706.09(41146.76,83164.92) | 344.78(241.66,488.43) | 9.96(-1.22,21.41) | 0.20(0.15,0.26) |
| People's Republic of Bangladesh | 198402.43(161037.69,241612.20) | 470.04(381.52,572.41) | 375317.08(277940.92,495320.81) | 545.41(403.90,719.80) | 16.04(2.33,31.21) | 0.03(-0.14,0.21) |
| People's Republic of China | 2391276.67(1656838.35,3311699.93) | 436.25(302.27,604.17) | 2307449.78(1663795.64,3213736.67) | 500.05(360.56,696.45) | 14.62(6.06,24.62) | 0.79(0.57,1.01) |
| Plurinational State of Bolivia | 11604.75(9778.27,13843.29) | 470.58(396.52,561.35) | 23993.56(18061.71,31490.82) | 488.27(367.56,640.84) | 3.76(-12.81,19.51) | -0.18(-0.31,-0.05) |
| Portuguese Republic | 9025.73(6020.12,12847.99) | 238.39(159.01,339.35) | 9174.76(6804.61,11878.37) | 310.84(230.54,402.44) | 30.39(11.67,58.47) | 0.99(0.85,1.13) |
| Principality of Andorra | 87.30(59.42,122.52) | 349.09(237.60,489.89) | 94.15(64.78,131.46) | 369.26(254.08,515.57) | 5.78(-1.07,14.18) | 0.15(0.10,0.21) |
| Principality of Monaco | 30.90(20.82,43.59) | 337.72(227.60,476.49) | 34.34(23.22,48.86) | 368.43(249.11,524.11) | 9.10(2.68,15.80) | 0.36(0.31,0.41) |
| Puerto Rico | 3821.05(2657.76,5312.30) | 270.12(187.88,375.54) | 3792.64(2619.51,5346.00) | 366.70(253.27,516.89) | 35.75(24.62,45.85) | 1.03(0.99,1.07) |
| Republic of Albania | 4267.26(2881.06,6071.41) | 300.41(202.82,427.42) | 2990.39(2030.28,4205.63) | 315.42(214.15,443.60) | 5.00(-2.45,12.27) | 0.31(0.24,0.39) |
| Republic of Angola | 4977.13(3569.82,6706.23) | 127.21(91.24,171.40) | 20052.85(14133.51,27709.31) | 164.83(116.17,227.76) | 29.57(20.93,37.97) | 0.77(0.67,0.88) |
| Republic of Armenia | 3805.73(2646.87,5281.61) | 264.80(184.17,367.49) | 3141.03(2169.37,4516.07) | 292.18(201.79,420.08) | 10.34(2.10,20.65) | 0.33(0.27,0.39) |
| Republic of Austria | 11507.96(7578.49,16336.28) | 383.35(252.45,544.18) | 11918.55(8747.06,15763.23) | 422.27(309.91,558.49) | 10.15(-0.72,27.05) | 0.52(0.40,0.64) |
| Republic of Azerbaijan | 8389.28(5955.63,11344.06) | 263.97(187.39,356.94) | 11581.14(8000.13,16291.44) | 273.40(188.87,384.60) | 3.57(-5.79,14.16) | 0.11(-0.04,0.27) |
| Republic of Belarus | 9801.25(6868.16,14059.86) | 248.48(174.12,356.44) | 8664.96(6011.02,12420.35) | 295.36(204.90,423.37) | 18.87(9.69,29.48) | 0.66(0.58,0.74) |
| Republic of Benin | 2204.09(1523.87,3071.01) | 129.44(89.49,180.35) | 8157.70(5500.25,11318.88) | 155.61(104.92,215.90) | 20.22(12.44,29.78) | 0.53(0.44,0.62) |
| Republic of Botswana | 1000.83(725.98,1351.25) | 194.43(141.03,262.50) | 2200.59(1583.06,2999.79) | 206.56(148.59,281.57) | 6.24(-2.60,14.85) | 0.18(0.08,0.28) |
| Republic of Bulgaria | 9031.53(6245.16,12864.45) | 303.39(209.79,432.14) | 5919.08(4157.59,8423.54) | 311.59(218.87,443.44) | 2.71(-5.06,12.03) | -0.01(-0.05,0.03) |
| Republic of Burundi | 2798.57(1943.77,3803.08) | 135.00(93.77,183.46) | 8602.71(6158.66,11360.74) | 163.17(116.81,215.48) | 20.87(13.66,29.99) | 0.60(0.54,0.65) |
| Republic of Cabo Verde | 174.81(114.37,248.85) | 133.68(87.46,190.31) | 420.95(286.56,596.15) | 167.96(114.34,237.87) | 25.64(13.77,41.75) | 0.81(0.72,0.90) |
| Republic of Cameroon | 5104.54(3552.69,7051.64) | 134.21(93.41,185.41) | 20158.72(13960.13,28738.09) | 156.36(108.28,222.90) | 16.50(8.20,25.30) | 0.40(0.29,0.51) |
| Republic of Chad | 2769.52(1956.28,3809.60) | 131.94(93.20,181.49) | 9410.47(6645.55,12992.24) | 149.54(105.60,206.46) | 13.34(5.75,22.23) | 0.36(0.25,0.46) |
| Republic of Chile | 18919.81(13242.88,26209.24) | 330.34(231.22,457.62) | 28229.76(19658.23,39694.22) | 398.98(277.84,561.01) | 20.78(11.39,28.71) | 0.47(0.44,0.51) |
| Republic of Colombia | 36598.01(25764.13,49725.60) | 260.41(183.32,353.81) | 72728.34(52453.22,99767.16) | 361.94(261.04,496.50) | 38.99(28.67,49.15) | 1.05(1.03,1.08) |
| Republic of Costa Rica | 3803.13(2661.68,5210.36) | 296.06(207.20,405.60) | 6902.75(4930.13,9666.88) | 362.68(259.03,507.91) | 22.50(12.89,33.47) | 0.64(0.62,0.66) |
| Republic of Croatia | 6761.24(5601.27,7916.97) | 372.62(308.69,436.31) | 4157.15(3883.93,4424.77) | 333.06(311.17,354.50) | -10.62(-21.99,5.81) | -0.31(-0.36,-0.27) |
| Republic of Cuba | 15843.61(11295.65,21422.51) | 324.69(231.48,439.02) | 14228.16(10218.72,19931.87) | 396.81(284.99,555.88) | 22.21(11.81,35.32) | 0.45(0.36,0.54) |
| Republic of Cyprus | 704.39(468.80,1009.62) | 229.03(152.43,328.27) | 1077.32(797.10,1472.96) | 214.71(158.87,293.57) | -6.25(-15.16,6.11) | -0.27(-0.37,-0.18) |
| Republic of Côte d'Ivoire | 6145.63(4185.97,8562.19) | 129.93(88.50,181.02) | 17224.22(11969.66,23854.48) | 153.63(106.76,212.76) | 18.24(10.28,26.09) | 0.51(0.41,0.61) |
| Republic of Djibouti | 257.08(175.78,365.91) | 146.63(100.26,208.71) | 944.87(647.57,1341.72) | 174.73(119.75,248.11) | 19.16(8.92,30.32) | 0.44(0.31,0.57) |
| Republic of Ecuador | 23321.07(19373.63,28141.56) | 565.18(469.51,682.00) | 45733.77(33808.93,60501.83) | 626.11(462.85,828.29) | 10.78(-6.08,27.09) | -0.09(-0.26,0.09) |
| Republic of El Salvador | 8272.35(6337.77,10707.41) | 395.94(303.34,512.48) | 8488.92(6056.77,11615.12) | 327.51(233.68,448.12) | -17.28(-27.82,-6.58) | -1.23(-1.47,-0.98) |
| Republic of Equatorial Guinea | 188.35(136.37,256.82) | 124.73(90.31,170.08) | 1191.74(818.65,1662.67) | 171.32(117.69,239.02) | 37.35(24.86,49.15) | 1.00(0.92,1.07) |
| Republic of Estonia | 1484.88(1038.31,2083.54) | 261.43(182.81,366.83) | 1174.26(814.11,1716.22) | 296.94(205.87,433.99) | 13.58(5.48,22.23) | 0.51(0.42,0.60) |
| Republic of Fiji | 582.77(403.74,814.58) | 180.73(125.21,252.62) | 728.69(516.43,1014.04) | 204.27(144.77,284.26) | 13.02(4.05,21.58) | 0.31(0.24,0.37) |
| Republic of Finland | 8647.25(6549.67,11298.68) | 476.32(360.78,622.37) | 7690.68(5976.02,9805.56) | 461.63(358.70,588.57) | -3.09(-8.73,2.99) | -0.14(-0.21,-0.07) |
| Republic of Ghana | 9934.35(7224.81,13348.41) | 173.07(125.86,232.54) | 27940.61(20176.11,38036.81) | 195.37(141.07,265.96) | 12.88(5.99,20.44) | 0.15(0.08,0.23) |
| Republic of Guatemala | 19111.97(16412.67,22183.17) | 647.00(555.62,750.97) | 27048.48(20870.96,34115.07) | 397.38(306.62,501.20) | -38.58(-47.72,-28.89) | -2.46(-2.82,-2.09) |
| Republic of Guinea | 2658.92(1891.33,3668.37) | 129.42(92.06,178.56) | 7952.54(5401.65,11103.99) | 153.91(104.54,214.90) | 18.92(10.92,27.45) | 0.50(0.42,0.58) |
| Republic of Guinea-Bissau | 531.01(382.03,710.36) | 143.19(103.02,191.55) | 1280.44(910.67,1773.03) | 151.75(107.93,210.13) | 5.98(-1.14,13.52) | 0.01(-0.08,0.10) |
| Republic of Guyana | 991.03(767.93,1256.54) | 291.17(225.62,369.18) | 1094.03(833.47,1389.91) | 352.08(268.22,447.30) | 20.92(13.89,28.28) | 0.32(0.20,0.43) |
| Republic of Haiti | 5176.95(4056.64,6533.86) | 212.67(166.65,268.41) | 12941.17(10091.84,16629.64) | 235.75(183.84,302.94) | 10.85(4.27,17.62) | 0.19(0.11,0.28) |
| Republic of Honduras | 7652.21(6296.77,9411.56) | 443.23(364.72,545.14) | 15261.06(11642.38,19865.13) | 347.19(264.86,451.93) | -21.67(-30.81,-12.69) | -1.15(-1.31,-1.00) |
| Republic of Iceland | 361.50(245.92,513.91) | 347.99(236.73,494.70) | 545.21(401.95,684.10) | 455.59(335.88,571.65) | 30.92(11.76,59.82) | 0.96(0.72,1.20) |
| Republic of India | 2020250.77(1609437.45,2535702.17) | 592.40(471.94,743.55) | 2972773.19(2334072.00,3778073.13) | 487.78(382.98,619.91) | -17.66(-21.37,-13.31) | -1.02(-1.15,-0.88) |
| Republic of Indonesia | 250288.44(182745.94,332684.86) | 320.70(234.16,426.28) | 308162.96(226870.90,406308.80) | 270.62(199.23,356.80) | -15.62(-18.31,-12.59) | -0.64(-0.70,-0.59) |
| Republic of Iraq | 21409.44(14232.53,30696.10) | 297.97(198.08,427.21) | 63502.92(43272.58,90237.82) | 364.20(248.17,517.52) | 22.23(15.02,30.87) | 0.63(0.57,0.69) |
| Republic of Italy | 86225.42(58709.07,120991.60) | 403.90(275.00,566.75) | 54757.75(42711.74,69382.79) | 346.68(270.41,439.27) | -14.17(-27.84,4.14) | -0.89(-1.06,-0.72) |
| Republic of Kazakhstan | 18135.56(12816.69,24313.23) | 267.13(188.79,358.13) | 19987.43(13910.81,28521.75) | 286.79(199.60,409.24) | 7.36(-1.27,17.17) | 0.32(0.17,0.47) |
| Republic of Kenya | 11754.20(8075.89,16338.68) | 134.22(92.22,186.57) | 31261.30(21635.03,43092.69) | 144.38(99.92,199.03) | 7.57(3.97,12.26) | -0.04(-0.20,0.11) |
| Republic of Kiribati | 58.62(42.74,77.22) | 191.99(140.00,252.92) | 103.90(77.35,134.74) | 208.95(155.56,270.98) | 8.83(1.85,15.88) | 0.07(-0.01,0.15) |
| Republic of Korea | 124168.86(86702.68,174470.42) | 589.93(411.93,828.91) | 93375.39(63817.06,133117.54) | 583.54(398.82,831.91) | -1.08(-8.87,7.39) | -0.09(-0.11,-0.07) |
| Republic of Latvia | 2554.01(1781.33,3617.78) | 267.72(186.73,379.23) | 1592.00(1097.30,2324.13) | 295.58(203.73,431.50) | 10.40(2.92,18.74) | 0.33(0.23,0.42) |
| Republic of Liberia | 1179.33(800.31,1645.59) | 127.83(86.75,178.37) | 3691.33(2530.31,5215.70) | 164.41(112.70,232.31) | 28.62(20.05,38.44) | 0.79(0.72,0.87) |
| Republic of Lithuania | 3653.91(2519.67,5116.04) | 262.24(180.84,367.18) | 2413.63(1679.46,3395.15) | 299.79(208.60,421.71) | 14.32(6.48,24.73) | 0.46(0.37,0.56) |
| Republic of Madagascar | 6637.73(4634.47,9196.13) | 146.54(102.31,203.02) | 19603.04(13575.85,27084.11) | 167.39(115.93,231.28) | 14.23(7.25,24.11) | 0.28(0.16,0.39) |
| Republic of Malawi | 5344.72(3725.65,7257.86) | 143.04(99.71,194.24) | 14106.06(10063.68,19384.55) | 172.38(122.98,236.88) | 20.51(12.89,29.94) | 0.50(0.40,0.59) |
| Republic of Maldives | 150.35(100.41,211.65) | 185.01(123.55,260.44) | 519.56(352.33,745.67) | 199.70(135.42,286.62) | 7.94(-8.56,27.62) | 0.24(0.18,0.30) |
| Republic of Mali | 3790.51(2603.41,5242.64) | 126.96(87.20,175.59) | 13583.55(9181.92,19740.63) | 152.50(103.09,221.63) | 20.12(11.82,29.98) | 0.49(0.40,0.58) |
| Republic of Malta | 396.85(272.56,557.82) | 287.59(197.52,404.24) | 467.92(399.03,521.68) | 349.57(298.10,389.73) | 21.55(-11.02,74.79) | 0.83(0.56,1.10) |
| Republic of Mauritius | 932.47(615.96,1324.99) | 187.58(123.91,266.54) | 953.36(647.16,1377.67) | 209.43(142.17,302.65) | 11.65(4.35,19.54) | 0.25(0.18,0.32) |
| Republic of Moldova | 4532.08(3229.31,6451.87) | 260.04(185.29,370.20) | 3597.12(2516.14,5192.46) | 290.03(202.87,418.66) | 11.53(2.26,21.35) | 0.33(0.22,0.43) |
| Republic of Mozambique | 6563.40(4657.66,9048.15) | 138.38(98.20,190.77) | 21760.10(15864.94,28693.90) | 180.98(131.95,238.65) | 30.78(20.99,43.31) | 0.72(0.62,0.81) |
| Republic of Namibia | 1017.94(725.22,1397.25) | 182.03(129.69,249.86) | 2164.26(1509.42,2990.70) | 207.09(144.43,286.17) | 13.77(6.18,22.12) | 0.37(0.24,0.50) |
| Republic of Nauru | 7.83(5.68,10.45) | 193.56(140.48,258.42) | 10.22(7.38,13.61) | 219.69(158.64,292.42) | 13.50(6.26,20.78) | 0.24(0.13,0.34) |
| Republic of Nicaragua | 4194.78(3018.52,5655.97) | 284.19(204.50,383.18) | 9070.75(6521.98,12515.50) | 318.70(229.15,439.74) | 12.14(2.83,20.99) | 0.29(0.23,0.35) |
| Republic of Niue | 1.50(1.03,2.11) | 186.08(128.24,261.94) | 1.18(0.83,1.64) | 207.87(145.70,288.41) | 11.71(5.68,19.65) | 0.22(0.18,0.26) |
| Republic of Palau | 14.07(9.91,19.29) | 201.66(142.04,276.49) | 14.45(10.61,19.77) | 245.41(180.19,335.74) | 21.70(11.00,35.00) | 0.39(0.30,0.49) |
| Republic of Panama | 3144.06(2237.23,4268.58) | 310.81(221.17,421.98) | 5845.84(4181.20,8063.72) | 354.20(253.34,488.58) | 13.96(6.40,21.12) | 0.20(0.10,0.29) |
| Republic of Paraguay | 5784.78(4270.09,7652.47) | 369.09(272.45,488.26) | 9476.22(6757.42,12942.86) | 309.73(220.86,423.03) | -16.08(-22.15,-10.53) | -0.72(-0.93,-0.51) |
| Republic of Peru | 77464.03(66050.94,90995.89) | 873.20(744.55,1025.73) | 100905.37(72201.43,135233.79) | 678.99(485.84,909.99) | -22.24(-37.17,-5.82) | -1.72(-2.12,-1.33) |
| Republic of Poland | 59632.90(41719.43,84283.23) | 412.83(288.82,583.48) | 43897.62(35613.18,53825.91) | 362.83(294.36,444.90) | -12.11(-27.22,6.75) | -0.80(-0.99,-0.62) |
| Republic of Rwanda | 4148.63(3003.66,5525.56) | 151.38(109.60,201.62) | 9568.43(6821.57,13000.50) | 168.71(120.28,229.23) | 11.45(2.46,23.11) | 0.25(0.17,0.33) |
| Republic of San Marino | 33.86(22.49,47.71) | 360.58(239.45,508.03) | 34.11(23.10,47.88) | 380.56(257.75,534.23) | 5.54(0.11,11.64) | 0.20(0.11,0.30) |
| Republic of Senegal | 3771.78(2632.42,5257.43) | 136.69(95.40,190.52) | 10178.40(6889.94,14356.21) | 157.86(106.86,222.65) | 15.49(8.04,22.48) | 0.28(0.19,0.38) |
| Republic of Serbia | 8545.42(5892.07,12149.74) | 237.96(164.07,338.33) | 8541.16(6809.16,10583.76) | 288.13(229.71,357.04) | 21.09(-2.13,53.17) | 0.61(0.42,0.80) |
| Republic of Seychelles | 68.19(48.13,94.33) | 218.43(154.18,302.16) | 94.26(67.97,131.93) | 245.55(177.08,343.69) | 12.42(3.95,21.60) | 0.30(0.23,0.36) |
| Republic of Sierra Leone | 2106.45(1425.67,2947.35) | 131.87(89.25,184.51) | 6147.34(4217.53,8428.84) | 164.82(113.08,225.99) | 24.99(16.17,36.41) | 0.68(0.57,0.79) |
| Republic of Singapore | 8960.76(6025.89,12660.65) | 593.72(399.26,838.86) | 10491.34(7342.18,15160.60) | 545.37(381.67,788.09) | -8.14(-16.94,2.30) | -0.23(-0.32,-0.15) |
| Republic of Slovenia | 2382.58(1637.99,3426.03) | 310.91(213.74,447.07) | 2163.76(1938.99,2360.68) | 380.01(340.53,414.59) | 22.23(-12.05,75.08) | 0.22(0.02,0.42) |
| Republic of South Africa | 34587.68(24622.93,46918.76) | 219.80(156.48,298.17) | 50089.80(35736.84,68127.40) | 206.58(147.39,280.97) | -6.02(-11.08,-0.73) | -0.31(-0.37,-0.24) |
| Republic of South Sudan | 3042.29(2121.82,4263.55) | 131.79(91.91,184.69) | 5613.58(3851.03,7805.05) | 155.97(107.00,216.86) | 18.35(9.92,28.91) | 0.42(0.33,0.51) |
| Republic of Sudan | 20479.06(14324.60,28364.35) | 268.73(187.97,372.21) | 60327.69(41672.81,84433.81) | 326.35(225.43,456.75) | 21.44(13.62,29.13) | 0.55(0.46,0.64) |
| Republic of Suriname | 413.28(291.53,554.53) | 253.79(179.02,340.53) | 661.65(477.12,909.85) | 308.24(222.28,423.87) | 21.46(11.79,30.34) | 0.61(0.56,0.66) |
| Republic of Tajikistan | 5674.93(4068.94,7757.52) | 268.36(192.42,366.85) | 11300.47(8045.47,15507.64) | 270.92(192.88,371.78) | 0.95(-6.28,8.84) | -0.10(-0.22,0.02) |
| Republic of the Congo | 1214.57(866.52,1631.85) | 128.21(91.47,172.26) | 3705.59(2583.05,5150.04) | 167.25(116.58,232.44) | 30.45(20.92,40.74) | 0.88(0.81,0.95) |
| Republic of the Gambia | 524.45(358.22,729.68) | 139.10(95.01,193.53) | 1614.69(1101.03,2289.39) | 161.45(110.09,228.91) | 16.07(6.57,25.00) | 0.39(0.31,0.48) |
| Republic of the Marshall Islands | 33.26(24.13,44.32) | 193.90(140.66,258.38) | 49.56(36.05,66.82) | 208.93(151.98,281.67) | 7.75(1.41,14.99) | 0.19(0.12,0.26) |
| Republic of the Niger | 3682.05(2562.89,5131.00) | 132.29(92.08,184.35) | 13655.82(9338.83,19060.88) | 153.14(104.73,213.75) | 15.76(7.78,27.32) | 0.43(0.34,0.51) |
| Republic of the Philippines | 46842.08(33258.39,63906.93) | 180.72(128.32,246.56) | 90511.70(64450.57,123695.84) | 191.56(136.40,261.79) | 5.99(3.34,8.12) | 0.07(-0.01,0.14) |
| Republic of the Union of Myanmar | 39903.44(30583.81,51753.55) | 232.44(178.15,301.46) | 56453.05(41722.91,75624.16) | 251.11(185.59,336.38) | 8.03(0.76,16.58) | 0.18(0.13,0.23) |
| Republic of Trinidad and Tobago | 1276.82(902.81,1771.85) | 254.68(180.08,353.42) | 1627.10(1151.67,2332.26) | 326.87(231.36,468.53) | 28.35(17.84,38.90) | 0.87(0.81,0.92) |
| Republic of Tunisia | 10558.72(7152.76,14807.79) | 306.96(207.94,430.48) | 15154.29(10543.00,21534.58) | 348.23(242.27,494.85) | 13.45(3.40,26.24) | 0.36(0.31,0.41) |
| Republic of Turkey | 68106.46(45884.66,97453.46) | 284.74(191.83,407.43) | 111218.93(76889.70,155206.39) | 349.09(241.34,487.16) | 22.60(14.75,32.57) | 0.64(0.58,0.70) |
| Republic of Uganda | 8985.16(6283.89,12335.78) | 140.08(97.97,192.32) | 29650.75(20919.41,40122.13) | 172.40(121.63,233.29) | 23.07(13.84,33.99) | 0.66(0.56,0.76) |
| Republic of Uzbekistan | 23163.28(16484.72,31955.05) | 269.86(192.05,372.29) | 36903.40(25890.16,52280.38) | 268.64(188.47,380.58) | -0.45(-9.55,8.87) | 0.01(-0.16,0.17) |
| Republic of Vanuatu | 119.30(86.77,160.27) | 203.66(148.12,273.59) | 264.20(195.28,345.66) | 211.97(156.67,277.33) | 4.08(-1.70,11.54) | -0.01(-0.07,0.04) |
| Republic of Yemen | 12485.81(8686.84,17223.81) | 271.61(188.97,374.68) | 42414.33(29324.83,59811.10) | 308.25(213.12,434.68) | 13.49(7.64,20.15) | 0.37(0.30,0.45) |
| Republic of Zambia | 4460.41(3188.77,6080.67) | 147.00(105.09,200.40) | 14227.41(9916.09,19478.17) | 175.80(122.53,240.68) | 19.59(10.47,28.88) | 0.50(0.41,0.60) |
| Republic of Zimbabwe | 7042.21(4906.89,9764.94) | 177.63(123.77,246.30) | 11724.72(8289.75,16023.24) | 185.00(130.80,252.83) | 4.15(-4.07,12.87) | -0.14(-0.30,0.03) |
| Romania | 25891.62(17884.18,35983.78) | 298.05(205.87,414.22) | 16653.38(11791.21,23885.50) | 309.01(218.79,443.21) | 3.68(-3.41,10.38) | -0.06(-0.12,0.01) |
| Russian Federation | 167699.90(118043.54,236504.99) | 288.29(202.92,406.56) | 145178.37(103085.34,209373.89) | 312.38(221.81,450.51) | 8.36(3.15,14.36) | 0.31(0.20,0.42) |
| Saint Kitts and Nevis | 48.31(34.77,64.92) | 279.57(201.18,375.65) | 77.95(55.20,109.55) | 342.96(242.89,482.02) | 22.68(12.47,35.01) | 0.41(0.32,0.49) |
| Saint Lucia | 152.17(105.94,210.38) | 270.48(188.31,373.95) | 233.68(167.04,312.14) | 353.56(252.73,472.26) | 30.72(19.13,42.07) | 0.77(0.72,0.83) |
| Saint Vincent and the Grenadines | 114.60(79.13,157.68) | 249.50(172.28,343.29) | 132.16(93.17,184.15) | 319.84(225.48,445.66) | 28.19(16.25,39.22) | 0.76(0.70,0.82) |
| Slovak Republic | 6392.89(4342.44,9184.98) | 312.30(212.13,448.69) | 6729.92(4991.09,8701.17) | 393.26(291.65,508.45) | 25.93(8.68,48.37) | 0.70(0.40,0.99) |
| Socialist Republic of Viet Nam | 58907.53(41207.54,81975.27) | 206.57(144.50,287.45) | 86664.41(60620.94,120876.73) | 225.75(157.91,314.86) | 9.29(-1.85,21.89) | 0.17(0.11,0.23) |
| Solomon Islands | 225.42(160.14,307.00) | 175.74(124.85,239.34) | 544.21(392.59,723.62) | 198.97(143.53,264.56) | 13.22(6.30,21.26) | 0.25(0.19,0.32) |
| State of Eritrea | 1783.98(1247.70,2433.10) | 137.66(96.28,187.75) | 4650.78(3353.61,6221.69) | 166.10(119.77,222.21) | 20.66(11.97,30.43) | 0.44(0.34,0.53) |
| State of Israel | 6712.49(4486.18,9681.14) | 351.26(234.76,506.61) | 12442.90(8296.89,17604.30) | 374.44(249.68,529.76) | 6.60(1.17,13.48) | 0.16(0.12,0.19) |
| State of Kuwait | 2728.97(1820.96,3848.22) | 322.91(215.47,455.35) | 7518.74(5154.85,10906.00) | 354.20(242.84,513.77) | 9.69(-0.86,20.82) | 0.29(0.23,0.36) |
| State of Libya | 5269.00(3608.99,7367.87) | 313.59(214.80,438.51) | 10437.04(7370.49,14525.05) | 347.84(245.64,484.08) | 10.92(1.89,22.06) | 0.29(0.23,0.34) |
| State of Qatar | 727.93(497.23,1047.63) | 307.86(210.29,443.06) | 6114.56(4210.88,9068.18) | 370.02(254.82,548.75) | 20.19(10.19,34.62) | 0.79(0.72,0.86) |
| Sultanate of Oman | 2620.81(1788.67,3660.24) | 315.88(215.58,441.15) | 8414.42(5755.39,12438.03) | 363.58(248.69,537.44) | 15.10(3.95,27.83) | 0.48(0.43,0.53) |
| Swiss Confederation | 13001.65(10562.74,15588.07) | 493.43(400.87,591.59) | 14456.84(11779.77,17370.24) | 520.68(424.26,625.61) | 5.52(0.01,10.86) | 0.24(0.18,0.31) |
| Syrian Arab Republic | 15436.67(10775.13,21754.80) | 321.77(224.60,453.47) | 18741.12(13009.27,26468.76) | 368.47(255.78,520.41) | 14.51(3.73,24.27) | 0.24(0.13,0.36) |
| Taiwan (Province of China) | 11937.30(7960.77,17457.91) | 129.38(86.28,189.21) | 14294.03(10699.40,19249.97) | 189.46(141.81,255.14) | 46.43(26.86,74.06) | 1.32(1.27,1.37) |
| Togolese Republic | 1816.62(1251.71,2499.40) | 132.52(91.31,182.32) | 5232.10(3604.21,7306.66) | 155.51(107.12,217.17) | 17.35(9.28,26.68) | 0.45(0.34,0.57) |
| Tokelau | 1.00(0.70,1.39) | 172.82(120.84,239.21) | 0.99(0.70,1.37) | 201.10(141.07,276.48) | 16.36(8.78,24.13) | 0.38(0.33,0.43) |
| Turkmenistan | 4070.38(2938.15,5541.28) | 265.16(191.40,360.98) | 5750.27(3995.87,8056.04) | 276.47(192.12,387.33) | 4.27(-3.45,13.14) | 0.03(-0.12,0.18) |
| Tuvalu | 6.38(4.47,8.70) | 176.87(123.96,241.07) | 10.63(7.64,14.54) | 213.94(153.66,292.68) | 20.96(13.58,29.42) | 0.44(0.39,0.50) |
| Ukraine | 58825.47(41073.79,82518.16) | 309.70(216.24,434.43) | 43196.52(30703.81,61789.81) | 313.36(222.73,448.24) | 1.18(-6.64,10.95) | 0.06(-0.08,0.19) |
| Union of the Comoros | 258.41(181.70,356.07) | 149.59(105.19,206.13) | 532.78(368.48,738.93) | 171.98(118.95,238.53) | 14.97(6.74,24.20) | 0.34(0.25,0.42) |
| United Arab Emirates | 2837.56(1956.38,4008.36) | 296.84(204.66,419.32) | 13185.70(9319.16,19822.81) | 328.22(231.97,493.42) | 10.57(-6.45,32.12) | 0.35(0.26,0.44) |
| United Kingdom of Great Britain and Northern Ireland | 63452.68(45227.93,86678.92) | 303.60(216.40,414.74) | 67960.14(50895.69,89196.40) | 312.44(233.99,410.07) | 2.91(-2.88,10.36) | 0.20(0.16,0.24) |
| United Mexican States | 103439.77(75243.48,138388.93) | 290.06(210.99,388.06) | 172360.85(128654.44,227052.66) | 334.61(249.76,440.78) | 15.36(7.89,23.79) | 0.40(0.34,0.46) |
| United Republic of Tanzania | 13531.66(9178.31,18973.88) | 139.66(94.73,195.83) | 40383.03(28260.78,55370.74) | 173.07(121.12,237.30) | 23.92(15.10,34.18) | 0.56(0.47,0.66) |
| United States of America | 373564.73(274384.69,502879.73) | 365.63(268.56,492.20) | 358698.87(304117.46,421673.94) | 322.28(273.24,378.86) | -11.86(-27.34,8.12) | -0.46(-0.51,-0.40) |
| United States Virgin Islands | 136.70(101.34,180.92) | 345.08(255.83,456.71) | 87.88(63.06,122.46) | 379.98(272.65,529.49) | 10.11(1.48,18.76) | 0.07(-0.04,0.17) |

AYAs = adolescents and young adults, PC = percentage change, EAPC = estimated annual percentage changes.
