# Supplementary material for: Global burden and trends of appendicitis among adolescents and young adults: A systematic analysis for the Global Burden of Disease study 2021 and predictions to 2040
Source: Medicine (Baltimore). 2026 Jul 3;105(27):e49625. doi: 10.1097/MD.0000000000049625 (PMC13336947; doi:10.1097/MD.0000000000049625)
Supplement: Supplementary file 8 [file medi-105-e49625-s008.docx]

S4 Table. Death burden for appendicitis among AYAs by 204 countries or territories in 1990 and 2021, and PC from 1990 to 2021.

| Location | 1990 | | 2021 | |  |  |
| --- | --- | --- | --- | --- | --- | --- |
|  | Death case (95%UI) | Death rate (95%UI) | Death case (95%UI) | Death rate (95%UI) | Percentage change(95%UI) | EAPC |
| American Samoa | 0.05(0.03,0.07) | 0.25(0.17,0.37) | 0.03(0.02,0.05) | 0.18(0.12,0.26) | -28.66(-58.45,25.40) | -1.01(-1.35,-0.67) |
| Antigua and Barbuda | 0.08(0.07,0.09) | 0.29(0.25,0.33) | 0.06(0.05,0.07) | 0.17(0.14,0.21) | -42.65(-54.74,-25.41) | -0.37(-0.99,0.26) |
| Arab Republic of Egypt | 22.14(10.82,29.15) | 0.10(0.05,0.13) | 9.54(6.51,13.88) | 0.02(0.02,0.03) | -77.62(-85.54,-55.33) | -6.30(-7.17,-5.42) |
| Argentine Republic | 20.49(18.07,23.07) | 0.17(0.15,0.19) | 16.05(13.42,19.08) | 0.09(0.08,0.11) | -45.39(-55.97,-32.66) | -1.17(-1.69,-0.65) |
| Australia | 1.88(1.61,2.18) | 0.03(0.02,0.03) | 1.12(0.95,1.32) | 0.01(0.01,0.02) | -53.42(-62.37,-42.23) | -2.55(-2.70,-2.40) |
| Barbados | 0.29(0.26,0.33) | 0.27(0.24,0.30) | 0.12(0.09,0.16) | 0.12(0.09,0.16) | -54.87(-66.79,-37.85) | -2.42(-2.77,-2.07) |
| Belize | 0.18(0.16,0.20) | 0.25(0.22,0.28) | 0.45(0.38,0.54) | 0.24(0.20,0.28) | -3.01(-23.08,19.31) | 0.77(0.44,1.10) |
| Bermuda | 0.05(0.05,0.06) | 0.21(0.18,0.24) | 0.02(0.01,0.02) | 0.10(0.08,0.13) | -53.85(-64.96,-40.44) | -1.78(-2.71,-0.84) |
| Bolivarian Republic of Venezuela | 43.85(39.46,48.90) | 0.55(0.49,0.61) | 36.61(24.88,49.52) | 0.39(0.27,0.53) | -28.58(-52.17,-2.94) | -0.34(-0.78,0.10) |
| Bosnia and Herzegovina | 2.13(1.51,2.92) | 0.11(0.08,0.15) | 0.21(0.14,0.33) | 0.02(0.01,0.03) | -81.40(-89.28,-69.20) | -6.19(-6.74,-5.63) |
| Brunei Darussalam | 0.11(0.07,0.15) | 0.09(0.06,0.12) | 0.09(0.07,0.12) | 0.04(0.03,0.06) | -49.20(-65.75,-17.74) | -2.00(-2.33,-1.67) |
| Burkina Faso | 9.25(4.55,25.28) | 0.29(0.14,0.79) | 14.02(8.74,26.62) | 0.16(0.10,0.31) | -44.14(-66.73,-3.47) | -2.11(-2.53,-1.70) |
| Canada | 3.41(3.07,3.83) | 0.03(0.03,0.03) | 2.81(2.45,3.22) | 0.02(0.02,0.03) | -22.60(-35.08,-7.39) | -0.95(-1.17,-0.72) |
| Central African Republic | 4.41(1.65,7.78) | 0.42(0.16,0.75) | 9.54(3.71,17.10) | 0.44(0.17,0.78) | 3.21(-36.11,78.42) | 0.18(0.06,0.30) |
| Commonwealth of Dominica | 0.08(0.06,0.10) | 0.27(0.21,0.34) | 0.05(0.04,0.07) | 0.21(0.14,0.28) | -24.27(-50.06,11.84) | -0.19(-0.74,0.38) |
| Commonwealth of the Bahamas | 0.40(0.34,0.46) | 0.34(0.29,0.39) | 0.41(0.32,0.52) | 0.27(0.21,0.34) | -20.49(-42.02,5.18) | 0.08(-0.33,0.50) |
| Cook Islands | 0.01(0.00,0.01) | 0.07(0.04,0.10) | 0.00(0.00,0.00) | 0.03(0.02,0.05) | -55.58(-76.64,-11.75) | -2.17(-2.63,-1.71) |
| Czech Republic | 3.61(3.11,4.20) | 0.10(0.08,0.11) | 0.55(0.42,0.70) | 0.02(0.01,0.02) | -80.91(-85.63,-74.96) | -4.65(-5.11,-4.18) |
| Democratic People's Republic of Korea | 8.29(4.09,13.21) | 0.10(0.05,0.16) | 5.69(2.47,10.21) | 0.06(0.02,0.10) | -43.14(-70.36,18.71) | -2.05(-2.17,-1.94) |
| Democratic Republic of Sao Tome and Principe | 0.08(0.03,0.20) | 0.19(0.07,0.48) | 0.07(0.03,0.12) | 0.07(0.04,0.13) | -60.55(-87.73,48.00) | -3.47(-3.90,-3.04) |
| Democratic Republic of the Congo | 43.13(18.84,80.79) | 0.30(0.13,0.56) | 94.34(47.12,189.54) | 0.26(0.13,0.53) | -13.17(-49.99,60.32) | -0.27(-0.38,-0.15) |
| Democratic Republic of Timor-Leste | 1.81(0.83,4.81) | 0.57(0.26,1.51) | 1.87(1.04,4.05) | 0.33(0.18,0.71) | -42.27(-64.99,5.36) | -1.99(-2.67,-1.30) |
| Democratic Socialist Republic of Sri Lanka | 13.38(9.54,17.99) | 0.18(0.13,0.24) | 2.86(1.78,4.13) | 0.04(0.02,0.05) | -80.40(-88.95,-65.31) | -6.01(-6.38,-5.63) |
| Dominican Republic | 19.56(13.83,24.73) | 0.64(0.45,0.81) | 14.90(6.93,22.46) | 0.33(0.15,0.49) | -48.53(-72.34,-23.22) | -1.28(-1.82,-0.74) |
| Eastern Republic of Uruguay | 2.07(1.87,2.28) | 0.18(0.17,0.20) | 1.39(1.21,1.57) | 0.12(0.10,0.13) | -36.34(-45.74,-24.86) | -1.11(-1.51,-0.71) |
| Federal Democratic Republic of Ethiopia | 54.53(21.30,121.52) | 0.30(0.12,0.67) | 73.54(37.89,239.67) | 0.16(0.08,0.52) | -46.85(-70.87,34.87) | -2.40(-2.56,-2.24) |
| Federal Democratic Republic of Nepal | 156.06(80.60,363.42) | 2.14(1.10,4.97) | 78.50(47.70,138.85) | 0.59(0.36,1.04) | -72.60(-85.04,-47.00) | -4.37(-4.52,-4.22) |
| Federal Republic of Germany | 14.63(12.91,16.67) | 0.05(0.04,0.06) | 3.52(3.07,4.07) | 0.01(0.01,0.02) | -71.71(-76.13,-65.56) | -4.01(-4.17,-3.84) |
| Federal Republic of Nigeria | 73.15(35.28,146.00) | 0.21(0.10,0.43) | 78.36(42.24,139.46) | 0.09(0.05,0.16) | -59.33(-76.26,-36.50) | -3.33(-3.60,-3.06) |
| Federal Republic of Somalia | 9.63(3.67,25.29) | 0.33(0.13,0.87) | 25.28(8.42,65.35) | 0.30(0.10,0.79) | -8.21(-49.06,53.57) | -0.24(-0.34,-0.14) |
| Federated States of Micronesia | 0.17(0.08,0.26) | 0.42(0.19,0.66) | 0.11(0.05,0.17) | 0.25(0.12,0.39) | -40.13(-66.20,11.24) | -1.59(-1.70,-1.48) |
| Federative Republic of Brazil | 184.08(172.60,195.78) | 0.29(0.28,0.31) | 239.01(224.45,254.76) | 0.28(0.26,0.30) | -4.44(-12.67,4.61) | 0.13(-0.02,0.27) |
| French Republic | 9.40(8.22,10.68) | 0.04(0.04,0.05) | 2.63(2.28,3.06) | 0.01(0.01,0.02) | -69.04(-74.32,-62.33) | -3.62(-3.78,-3.46) |
| Gabonese Republic | 0.64(0.30,1.04) | 0.17(0.08,0.27) | 1.01(0.51,1.70) | 0.13(0.07,0.23) | -18.57(-56.07,68.92) | -0.62(-0.82,-0.42) |
| Georgia | 3.40(3.01,3.81) | 0.16(0.14,0.18) | 0.52(0.39,0.68) | 0.05(0.03,0.06) | -71.28(-79.77,-61.09) | -5.12(-6.16,-4.07) |
| Grand Duchy of Luxembourg | 0.07(0.06,0.08) | 0.05(0.04,0.06) | 0.02(0.02,0.03) | 0.01(0.01,0.01) | -80.41(-84.22,-75.61) | -5.52(-5.72,-5.31) |
| Greenland | 0.01(0.01,0.02) | 0.04(0.03,0.09) | 0.01(0.00,0.02) | 0.05(0.01,0.08) | 23.95(-70.97,176.80) | 1.00(0.62,1.39) |
| Grenada | 0.10(0.08,0.12) | 0.30(0.25,0.35) | 0.06(0.05,0.08) | 0.15(0.12,0.19) | -47.88(-59.80,-33.82) | -1.19(-1.94,-0.43) |
| Guam | 0.04(0.03,0.05) | 0.06(0.04,0.08) | 0.03(0.02,0.04) | 0.05(0.04,0.07) | -6.19(-42.91,42.99) | 0.35(0.06,0.65) |
| Hashemite Kingdom of Jordan | 1.19(0.72,1.73) | 0.08(0.05,0.11) | 1.19(0.83,1.85) | 0.02(0.02,0.03) | -71.56(-83.32,-38.08) | -4.65(-5.03,-4.27) |
| Hellenic Republic | 0.54(0.47,0.60) | 0.01(0.01,0.02) | 0.18(0.16,0.21) | 0.01(0.01,0.01) | -53.93(-61.51,-44.66) | -2.19(-2.37,-2.01) |
| Hungary | 5.94(5.06,6.91) | 0.16(0.14,0.19) | 0.64(0.52,0.77) | 0.02(0.02,0.03) | -85.63(-88.86,-81.26) | -5.97(-6.49,-5.45) |
| Independent State of Papua New Guinea | 2.54(0.78,7.88) | 0.15(0.05,0.48) | 4.36(2.05,10.17) | 0.10(0.05,0.24) | -33.75(-62.42,50.62) | -1.63(-1.85,-1.41) |
| Independent State of Samoa | 0.12(0.06,0.18) | 0.17(0.09,0.27) | 0.10(0.06,0.17) | 0.13(0.07,0.21) | -25.94(-61.02,27.41) | -0.94(-1.07,-0.80) |
| Ireland | 0.33(0.29,0.37) | 0.02(0.02,0.03) | 0.12(0.10,0.14) | 0.01(0.01,0.01) | -68.96(-74.33,-60.98) | -3.45(-3.82,-3.07) |
| Islamic Republic of Afghanistan | 18.99(6.24,34.03) | 0.60(0.20,1.08) | 40.29(17.09,68.35) | 0.33(0.14,0.56) | -45.32(-70.73,1.78) | -2.11(-2.42,-1.79) |
| Islamic Republic of Iran | 47.53(35.07,73.14) | 0.22(0.16,0.34) | 21.23(16.75,30.09) | 0.06(0.05,0.09) | -72.04(-82.91,-57.64) | -3.93(-4.09,-3.77) |
| Islamic Republic of Mauritania | 2.25(1.10,3.74) | 0.29(0.14,0.49) | 1.67(0.99,3.21) | 0.10(0.06,0.19) | -66.47(-80.48,-33.26) | -3.80(-3.93,-3.66) |
| Islamic Republic of Pakistan | 484.19(232.19,1158.52) | 1.19(0.57,2.84) | 747.28(478.27,1064.31) | 0.76(0.48,1.08) | -36.33(-72.84,22.45) | -2.13(-2.42,-1.85) |
| Jamaica | 1.81(1.60,2.07) | 0.18(0.16,0.21) | 1.62(1.13,2.25) | 0.14(0.09,0.19) | -26.18(-52.22,5.64) | -1.40(-1.84,-0.97) |
| Japan | 6.55(6.35,6.78) | 0.01(0.01,0.02) | 2.94(2.75,3.15) | 0.01(0.01,0.01) | -37.88(-41.76,-33.59) | -1.27(-1.41,-1.12) |
| Kingdom of Bahrain | 0.22(0.16,0.31) | 0.09(0.06,0.12) | 0.29(0.20,0.43) | 0.04(0.03,0.06) | -51.46(-71.08,-18.95) | -2.28(-2.70,-1.86) |
| Kingdom of Belgium | 1.04(0.93,1.16) | 0.03(0.02,0.03) | 0.30(0.26,0.35) | 0.01(0.01,0.01) | -69.38(-74.23,-63.14) | -3.95(-4.10,-3.80) |
| Kingdom of Bhutan | 4.33(1.84,9.84) | 1.61(0.68,3.65) | 1.68(0.82,4.09) | 0.49(0.24,1.18) | -69.77(-84.25,-34.15) | -4.17(-4.30,-4.04) |
| Kingdom of Cambodia | 52.99(25.20,87.65) | 1.38(0.65,2.28) | 45.24(23.48,80.20) | 0.62(0.32,1.11) | -54.62(-75.50,-8.34) | -2.83(-3.01,-2.64) |
| Kingdom of Denmark | 1.27(1.10,1.47) | 0.07(0.06,0.08) | 0.33(0.28,0.40) | 0.02(0.02,0.02) | -72.49(-78.26,-65.68) | -4.36(-4.65,-4.07) |
| Kingdom of Eswatini | 1.27(0.75,2.27) | 0.42(0.25,0.75) | 1.61(0.87,2.60) | 0.32(0.17,0.51) | -25.39(-69.61,38.10) | -0.74(-1.32,-0.16) |
| Kingdom of Lesotho | 2.09(1.09,5.85) | 0.39(0.20,1.08) | 3.34(1.93,5.15) | 0.40(0.23,0.62) | 3.50(-69.50,113.84) | 0.86(0.39,1.34) |
| Kingdom of Morocco | 25.43(13.43,44.01) | 0.24(0.13,0.42) | 13.47(7.19,24.85) | 0.09(0.05,0.17) | -62.50(-76.93,-30.50) | -3.27(-3.57,-2.97) |
| Kingdom of Norway | 0.51(0.48,0.54) | 0.03(0.03,0.03) | 0.19(0.17,0.20) | 0.01(0.01,0.01) | -66.73(-69.53,-62.79) | -3.51(-3.79,-3.24) |
| Kingdom of Saudi Arabia | 5.80(3.01,11.42) | 0.09(0.05,0.17) | 6.95(4.07,12.11) | 0.04(0.02,0.07) | -57.03(-82.01,2.20) | -2.32(-2.54,-2.09) |
| Kingdom of Spain | 6.77(6.04,7.62) | 0.05(0.04,0.05) | 1.47(1.26,1.71) | 0.01(0.01,0.01) | -74.00(-78.78,-68.96) | -4.64(-4.93,-4.34) |
| Kingdom of Sweden | 0.67(0.59,0.76) | 0.02(0.02,0.03) | 0.19(0.16,0.23) | 0.01(0.01,0.01) | -73.82(-78.65,-68.20) | -3.30(-3.67,-2.92) |
| Kingdom of Thailand | 57.70(35.44,95.60) | 0.22(0.14,0.37) | 22.77(12.54,36.08) | 0.11(0.06,0.17) | -51.73(-73.65,-18.64) | -3.35(-3.84,-2.86) |
| Kingdom of the Netherlands | 2.43(2.13,2.77) | 0.04(0.04,0.05) | 0.63(0.54,0.73) | 0.01(0.01,0.01) | -70.27(-75.98,-64.08) | -4.15(-4.39,-3.91) |
| Kingdom of Tonga | 0.07(0.04,0.14) | 0.18(0.11,0.38) | 0.06(0.04,0.10) | 0.16(0.09,0.25) | -10.98(-58.59,66.38) | -0.07(-0.31,0.17) |
| Kyrgyz Republic | 7.13(5.94,8.46) | 0.40(0.33,0.47) | 2.33(1.87,2.86) | 0.09(0.07,0.11) | -78.31(-83.69,-71.68) | -5.87(-6.72,-5.02) |
| Lao People's Democratic Republic | 11.11(5.02,17.84) | 0.72(0.32,1.15) | 9.73(5.17,18.14) | 0.30(0.16,0.57) | -57.82(-76.65,-19.87) | -2.96(-3.09,-2.83) |
| Lebanese Republic | 0.69(0.28,1.10) | 0.06(0.02,0.10) | 0.44(0.32,0.59) | 0.02(0.01,0.03) | -67.82(-81.65,-4.17) | -3.58(-3.80,-3.36) |
| Malaysia | 11.60(7.96,18.91) | 0.16(0.11,0.25) | 10.69(6.68,18.13) | 0.08(0.05,0.13) | -50.79(-66.38,-27.81) | -3.05(-3.39,-2.71) |
| Mongolia | 4.12(2.30,7.09) | 0.47(0.26,0.80) | 2.59(1.81,3.76) | 0.21(0.14,0.30) | -55.95(-76.55,-17.06) | -3.62(-3.95,-3.28) |
| Montenegro | 0.06(0.04,0.09) | 0.02(0.02,0.04) | 0.02(0.01,0.04) | 0.01(0.01,0.02) | -53.96(-74.63,-12.12) | -2.75(-3.19,-2.31) |
| New Zealand | 0.41(0.37,0.45) | 0.03(0.03,0.03) | 0.29(0.25,0.33) | 0.02(0.01,0.02) | -45.62(-53.88,-36.23) | -1.88(-2.25,-1.51) |
| North Macedonia | 0.26(0.19,0.37) | 0.03(0.02,0.05) | 0.08(0.05,0.12) | 0.01(0.01,0.02) | -69.07(-82.91,-49.58) | -3.92(-4.09,-3.75) |
| Northern Mariana Islands | 0.01(0.01,0.02) | 0.06(0.03,0.09) | 0.01(0.00,0.01) | 0.04(0.02,0.06) | -21.30(-54.26,61.26) | -0.61(-1.28,0.07) |
| Palestine | 1.25(0.53,3.39) | 0.16(0.07,0.44) | 0.90(0.44,2.26) | 0.04(0.02,0.10) | -74.63(-86.31,-51.81) | -4.46(-4.71,-4.22) |
| People's Democratic Republic of Algeria | 18.25(11.18,28.14) | 0.18(0.11,0.28) | 10.72(6.49,18.69) | 0.06(0.04,0.11) | -65.13(-79.50,-32.59) | -3.56(-3.69,-3.44) |
| People's Republic of Bangladesh | 645.12(387.20,1065.57) | 1.53(0.92,2.52) | 335.41(195.51,623.12) | 0.49(0.28,0.91) | -68.11(-81.44,-32.22) | -3.62(-3.78,-3.45) |
| People's Republic of China | 633.79(428.30,770.20) | 0.12(0.08,0.14) | 87.36(65.91,118.19) | 0.02(0.01,0.03) | -83.63(-88.95,-72.93) | -6.45(-6.80,-6.09) |
| Plurinational State of Bolivia | 45.20(21.29,73.06) | 1.83(0.86,2.96) | 27.83(17.09,45.38) | 0.57(0.35,0.92) | -69.11(-82.59,-38.57) | -4.06(-4.31,-3.82) |
| Portuguese Republic | 2.01(1.79,2.28) | 0.05(0.05,0.06) | 0.34(0.29,0.41) | 0.01(0.01,0.01) | -78.11(-82.03,-73.39) | -5.07(-5.25,-4.89) |
| Principality of Andorra | 0.01(0.00,0.01) | 0.02(0.01,0.03) | 0.00(0.00,0.00) | 0.01(0.01,0.01) | -54.04(-74.16,-8.34) | -2.26(-2.41,-2.12) |
| Principality of Monaco | 0.00(0.00,0.00) | 0.01(0.01,0.02) | 0.00(0.00,0.00) | 0.01(0.01,0.01) | -34.87(-60.83,10.85) | -1.63(-1.84,-1.41) |
| Puerto Rico | 2.15(1.91,2.40) | 0.15(0.14,0.17) | 0.60(0.48,0.73) | 0.06(0.05,0.07) | -61.60(-69.94,-51.16) | -2.58(-3.14,-2.02) |
| Republic of Albania | 0.83(0.58,1.17) | 0.06(0.04,0.08) | 0.12(0.08,0.20) | 0.01(0.01,0.02) | -77.59(-87.08,-59.37) | -4.62(-4.89,-4.36) |
| Republic of Angola | 11.59(4.00,21.48) | 0.30(0.10,0.55) | 25.57(12.42,43.75) | 0.21(0.10,0.36) | -29.02(-60.35,40.60) | -0.84(-1.04,-0.63) |
| Republic of Armenia | 3.85(3.18,4.57) | 0.27(0.22,0.32) | 0.50(0.40,0.63) | 0.05(0.04,0.06) | -82.66(-87.19,-76.52) | -6.50(-7.54,-5.46) |
| Republic of Austria | 1.20(1.07,1.35) | 0.04(0.04,0.05) | 0.30(0.26,0.35) | 0.01(0.01,0.01) | -73.09(-77.66,-67.87) | -4.12(-4.32,-3.93) |
| Republic of Azerbaijan | 11.09(8.22,13.95) | 0.35(0.26,0.44) | 2.63(1.62,4.86) | 0.06(0.04,0.11) | -82.22(-89.02,-68.16) | -6.46(-7.23,-5.70) |
| Republic of Belarus | 3.95(3.10,5.07) | 0.10(0.08,0.13) | 1.04(0.75,1.39) | 0.04(0.03,0.05) | -64.71(-76.81,-47.52) | -3.37(-3.56,-3.18) |
| Republic of Benin | 6.02(3.07,13.87) | 0.35(0.18,0.81) | 8.35(4.97,15.18) | 0.16(0.09,0.29) | -54.93(-74.14,-17.68) | -2.99(-3.26,-2.72) |
| Republic of Botswana | 2.54(1.31,5.75) | 0.49(0.26,1.12) | 1.77(0.96,3.55) | 0.17(0.09,0.33) | -66.28(-80.35,-41.53) | -3.59(-3.84,-3.34) |
| Republic of Bulgaria | 3.71(3.20,4.30) | 0.12(0.11,0.14) | 0.70(0.54,0.92) | 0.04(0.03,0.05) | -70.57(-78.76,-59.00) | -3.53(-3.92,-3.14) |
| Republic of Burundi | 6.45(2.24,15.72) | 0.31(0.11,0.76) | 12.09(4.88,31.85) | 0.23(0.09,0.60) | -26.30(-56.50,41.92) | -1.36(-1.52,-1.20) |
| Republic of Cabo Verde | 0.08(0.05,0.14) | 0.06(0.04,0.10) | 0.06(0.04,0.10) | 0.03(0.02,0.04) | -58.26(-75.43,-20.39) | -3.09(-3.37,-2.82) |
| Republic of Cameroon | 10.58(5.83,17.04) | 0.28(0.15,0.45) | 17.16(8.62,28.02) | 0.13(0.07,0.22) | -52.12(-73.66,-18.17) | -2.80(-3.06,-2.53) |
| Republic of Chad | 6.62(3.33,21.06) | 0.32(0.16,1.00) | 13.30(8.01,23.98) | 0.21(0.13,0.38) | -33.00(-64.15,33.23) | -1.60(-1.88,-1.32) |
| Republic of Chile | 13.16(11.68,14.76) | 0.23(0.20,0.26) | 7.03(6.11,8.11) | 0.10(0.09,0.11) | -56.78(-64.18,-47.81) | -2.56(-2.96,-2.16) |
| Republic of Colombia | 52.69(48.55,57.31) | 0.37(0.35,0.41) | 49.06(40.82,59.03) | 0.24(0.20,0.29) | -34.87(-47.43,-20.31) | -0.84(-1.20,-0.49) |
| Republic of Costa Rica | 2.40(2.08,2.75) | 0.19(0.16,0.21) | 3.93(3.31,4.66) | 0.21(0.17,0.24) | 10.48(-10.95,34.46) | 0.24(-0.23,0.71) |
| Republic of Croatia | 1.45(1.21,1.72) | 0.08(0.07,0.09) | 0.17(0.13,0.21) | 0.01(0.01,0.02) | -83.23(-87.30,-78.71) | -5.36(-5.68,-5.04) |
| Republic of Cuba | 20.42(18.38,22.66) | 0.42(0.38,0.46) | 8.65(7.26,10.38) | 0.24(0.20,0.29) | -42.37(-52.83,-28.66) | -1.20(-1.91,-0.48) |
| Republic of Cyprus | 0.07(0.04,0.10) | 0.02(0.01,0.03) | 0.03(0.02,0.05) | 0.01(0.00,0.01) | -70.93(-83.81,-44.35) | -4.70(-5.14,-4.25) |
| Republic of Côte d'Ivoire | 13.91(6.88,25.80) | 0.29(0.15,0.55) | 16.66(10.01,24.60) | 0.15(0.09,0.22) | -49.48(-71.26,-4.36) | -2.42(-2.70,-2.15) |
| Republic of Djibouti | 0.38(0.18,1.32) | 0.22(0.10,0.76) | 0.94(0.49,2.06) | 0.17(0.09,0.38) | -20.16(-55.35,43.52) | -0.82(-1.20,-0.43) |
| Republic of Ecuador | 72.44(64.89,80.64) | 1.76(1.57,1.95) | 24.40(18.75,31.42) | 0.33(0.26,0.43) | -80.98(-85.76,-75.47) | -4.69(-5.15,-4.24) |
| Republic of El Salvador | 15.91(12.79,19.85) | 0.76(0.61,0.95) | 9.11(6.29,12.32) | 0.35(0.24,0.48) | -53.83(-69.29,-33.52) | -2.02(-2.45,-1.59) |
| Republic of Equatorial Guinea | 0.70(0.30,1.28) | 0.46(0.20,0.85) | 0.93(0.46,1.58) | 0.13(0.07,0.23) | -71.03(-87.55,-20.61) | -4.60(-4.93,-4.27) |
| Republic of Estonia | 0.77(0.61,0.94) | 0.13(0.11,0.16) | 0.07(0.05,0.09) | 0.02(0.01,0.02) | -86.91(-90.36,-81.88) | -6.46(-6.92,-6.00) |
| Republic of Fiji | 0.86(0.61,1.23) | 0.27(0.19,0.38) | 0.73(0.47,1.07) | 0.21(0.13,0.30) | -23.10(-55.19,35.93) | -0.14(-0.60,0.32) |
| Republic of Finland | 0.94(0.82,1.09) | 0.05(0.04,0.06) | 0.26(0.22,0.31) | 0.02(0.01,0.02) | -69.44(-75.03,-62.08) | -3.32(-3.52,-3.11) |
| Republic of Ghana | 25.21(16.80,34.74) | 0.44(0.29,0.61) | 39.25(24.83,67.53) | 0.27(0.17,0.47) | -37.51(-63.69,14.69) | -1.39(-1.56,-1.22) |
| Republic of Guatemala | 56.91(50.93,62.61) | 1.93(1.72,2.12) | 53.96(44.76,64.92) | 0.79(0.66,0.95) | -58.85(-67.48,-48.73) | -2.72(-3.23,-2.20) |
| Republic of Guinea | 6.03(3.00,14.29) | 0.29(0.15,0.70) | 8.87(4.98,13.69) | 0.17(0.10,0.26) | -41.54(-71.22,2.87) | -2.07(-2.28,-1.85) |
| Republic of Guinea-Bissau | 2.31(1.03,3.59) | 0.62(0.28,0.97) | 2.56(1.41,3.94) | 0.30(0.17,0.47) | -51.25(-72.13,-10.85) | -2.50(-2.62,-2.37) |
| Republic of Guyana | 3.36(2.74,4.01) | 0.99(0.80,1.18) | 2.17(1.61,2.87) | 0.70(0.52,0.92) | -29.16(-48.70,-1.40) | 0.11(-0.30,0.51) |
| Republic of Haiti | 24.42(9.81,43.68) | 1.00(0.40,1.79) | 34.48(16.08,57.18) | 0.63(0.29,1.04) | -37.39(-60.86,-0.23) | -1.13(-1.38,-0.88) |
| Republic of Honduras | 20.68(12.33,31.25) | 1.20(0.71,1.81) | 22.69(11.55,37.45) | 0.52(0.26,0.85) | -56.91(-77.58,-26.87) | -2.97(-3.13,-2.81) |
| Republic of Iceland | 0.03(0.02,0.03) | 0.02(0.02,0.03) | 0.01(0.01,0.01) | 0.01(0.01,0.01) | -63.27(-69.89,-55.24) | -2.74(-3.01,-2.47) |
| Republic of India | 6407.10(4862.17,8134.21) | 1.88(1.43,2.39) | 2829.71(2028.73,3794.08) | 0.46(0.33,0.62) | -75.29(-84.53,-63.79) | -4.88(-5.33,-4.42) |
| Republic of Indonesia | 502.51(302.35,751.82) | 0.64(0.39,0.96) | 306.73(192.84,429.86) | 0.27(0.17,0.38) | -58.17(-71.18,-38.63) | -2.78(-2.91,-2.65) |
| Republic of Iraq | 2.69(1.50,4.12) | 0.04(0.02,0.06) | 2.27(1.54,3.51) | 0.01(0.01,0.02) | -65.13(-80.12,-22.59) | -3.44(-3.85,-3.03) |
| Republic of Italy | 5.35(5.12,5.60) | 0.03(0.02,0.03) | 1.17(1.08,1.30) | 0.01(0.01,0.01) | -70.55(-73.10,-66.80) | -3.84(-4.12,-3.57) |
| Republic of Kazakhstan | 18.74(15.50,22.80) | 0.28(0.23,0.34) | 5.72(4.45,8.06) | 0.08(0.06,0.12) | -70.27(-79.42,-55.40) | -5.32(-5.94,-4.70) |
| Republic of Kenya | 15.82(8.91,55.74) | 0.18(0.10,0.64) | 35.84(20.63,86.81) | 0.17(0.10,0.40) | -8.35(-40.46,37.34) | 0.22(-0.09,0.54) |
| Republic of Kiribati | 0.12(0.04,0.21) | 0.41(0.13,0.68) | 0.15(0.07,0.26) | 0.31(0.13,0.53) | -23.50(-58.94,34.48) | -1.08(-1.21,-0.96) |
| Republic of Korea | 33.94(21.45,42.74) | 0.16(0.10,0.20) | 3.38(2.47,4.78) | 0.02(0.02,0.03) | -86.91(-91.27,-77.22) | -5.91(-6.70,-5.10) |
| Republic of Latvia | 1.52(1.26,1.83) | 0.16(0.13,0.19) | 0.16(0.12,0.21) | 0.03(0.02,0.04) | -81.31(-86.64,-74.03) | -5.61(-6.13,-5.09) |
| Republic of Liberia | 2.63(1.30,4.89) | 0.28(0.14,0.53) | 3.19(1.88,6.00) | 0.14(0.08,0.27) | -50.05(-72.55,0.56) | -2.50(-2.81,-2.20) |
| Republic of Lithuania | 2.23(1.79,2.78) | 0.16(0.13,0.20) | 0.25(0.19,0.33) | 0.03(0.02,0.04) | -80.44(-86.08,-72.06) | -4.74(-5.20,-4.27) |
| Republic of Madagascar | 13.34(6.29,30.11) | 0.29(0.14,0.66) | 31.52(15.94,75.98) | 0.27(0.14,0.65) | -8.61(-46.05,55.53) | -0.23(-0.35,-0.11) |
| Republic of Malawi | 11.51(5.47,30.49) | 0.31(0.15,0.82) | 19.91(11.13,40.05) | 0.24(0.14,0.49) | -20.98(-51.34,31.37) | -0.70(-0.95,-0.46) |
| Republic of Maldives | 0.06(0.03,0.10) | 0.08(0.03,0.13) | 0.04(0.03,0.07) | 0.02(0.01,0.03) | -78.35(-87.95,-35.13) | -5.00(-5.26,-4.74) |
| Republic of Mali | 14.79(7.83,33.95) | 0.50(0.26,1.14) | 21.53(12.82,40.48) | 0.24(0.14,0.45) | -51.20(-70.41,-16.62) | -2.46(-2.64,-2.28) |
| Republic of Malta | 0.03(0.03,0.04) | 0.02(0.02,0.03) | 0.01(0.01,0.01) | 0.01(0.01,0.01) | -64.69(-72.40,-56.70) | -2.58(-2.80,-2.37) |
| Republic of Mauritius | 0.40(0.35,0.47) | 0.08(0.07,0.09) | 0.18(0.15,0.21) | 0.04(0.03,0.05) | -51.07(-61.65,-39.37) | -3.09(-3.51,-2.67) |
| Republic of Moldova | 3.04(2.53,3.64) | 0.17(0.15,0.21) | 0.43(0.31,0.58) | 0.03(0.03,0.05) | -80.22(-86.11,-72.43) | -5.18(-5.88,-4.48) |
| Republic of Mozambique | 14.21(6.34,45.46) | 0.30(0.13,0.96) | 46.12(22.70,90.79) | 0.38(0.19,0.76) | 28.07(-28.35,136.11) | 1.63(1.34,1.91) |
| Republic of Namibia | 2.43(1.28,5.76) | 0.43(0.23,1.03) | 2.19(1.11,3.96) | 0.21(0.11,0.38) | -51.64(-73.90,-8.43) | -2.65(-2.98,-2.31) |
| Republic of Nauru | 0.01(0.00,0.02) | 0.36(0.10,0.62) | 0.01(0.00,0.02) | 0.25(0.07,0.40) | -32.02(-61.57,13.11) | -1.25(-1.73,-0.77) |
| Republic of Nicaragua | 5.94(4.54,7.49) | 0.40(0.31,0.51) | 6.50(4.32,8.78) | 0.23(0.15,0.31) | -43.25(-62.94,-19.62) | -1.40(-1.68,-1.13) |
| Republic of Niue | 0.00(0.00,0.00) | 0.20(0.11,0.33) | 0.00(0.00,0.00) | 0.12(0.08,0.18) | -37.85(-66.53,25.25) | -2.04(-2.20,-1.87) |
| Republic of Palau | 0.01(0.01,0.02) | 0.20(0.07,0.35) | 0.01(0.00,0.02) | 0.18(0.07,0.31) | -8.52(-54.40,99.34) | -0.25(-0.31,-0.18) |
| Republic of Panama | 2.85(2.47,3.25) | 0.28(0.24,0.32) | 3.49(2.78,4.34) | 0.21(0.17,0.26) | -24.99(-41.97,-3.33) | -0.18(-0.59,0.23) |
| Republic of Paraguay | 6.34(4.71,8.24) | 0.40(0.30,0.53) | 9.42(6.44,12.94) | 0.31(0.21,0.42) | -23.86(-50.08,15.51) | -0.25(-0.50,0.01) |
| Republic of Peru | 184.45(131.75,231.52) | 2.08(1.49,2.61) | 48.68(33.95,69.67) | 0.33(0.23,0.47) | -84.25(-89.70,-72.54) | -6.12(-6.84,-5.40) |
| Republic of Poland | 14.30(13.40,15.30) | 0.10(0.09,0.11) | 2.31(2.09,2.54) | 0.02(0.02,0.02) | -80.74(-82.88,-78.11) | -5.16(-5.84,-4.47) |
| Republic of Rwanda | 9.30(3.01,15.51) | 0.34(0.11,0.57) | 10.91(4.98,35.36) | 0.19(0.09,0.62) | -43.30(-73.45,68.44) | -2.49(-2.79,-2.18) |
| Republic of San Marino | 0.00(0.00,0.00) | 0.02(0.01,0.02) | 0.00(0.00,0.00) | 0.01(0.00,0.01) | -50.87(-71.34,-11.56) | -1.56(-1.79,-1.33) |
| Republic of Senegal | 12.41(5.92,34.40) | 0.45(0.21,1.25) | 10.87(7.00,18.18) | 0.17(0.11,0.28) | -62.51(-79.44,-10.80) | -3.19(-3.39,-2.99) |
| Republic of Serbia | 2.72(1.84,3.71) | 0.08(0.05,0.10) | 0.59(0.41,0.81) | 0.02(0.01,0.03) | -73.51(-82.92,-53.90) | -4.29(-4.44,-4.15) |
| Republic of Seychelles | 0.17(0.13,0.21) | 0.54(0.43,0.67) | 0.08(0.06,0.12) | 0.22(0.16,0.30) | -59.62(-70.57,-42.82) | -2.60(-2.94,-2.26) |
| Republic of Sierra Leone | 4.33(1.81,10.14) | 0.27(0.11,0.63) | 6.29(4.04,10.91) | 0.17(0.11,0.29) | -37.68(-66.39,40.63) | -1.56(-1.68,-1.44) |
| Republic of Singapore | 0.74(0.64,0.84) | 0.05(0.04,0.06) | 0.21(0.18,0.25) | 0.01(0.01,0.01) | -77.53(-81.69,-71.75) | -4.50(-4.94,-4.04) |
| Republic of Slovenia | 0.68(0.57,0.81) | 0.09(0.07,0.11) | 0.06(0.05,0.08) | 0.01(0.01,0.01) | -87.87(-91.27,-83.68) | -6.39(-6.63,-6.14) |
| Republic of South Africa | 51.88(41.21,73.31) | 0.33(0.26,0.47) | 55.18(43.55,68.38) | 0.23(0.18,0.28) | -30.98(-50.83,-9.14) | -0.94(-1.92,0.05) |
| Republic of South Sudan | 5.27(2.30,18.68) | 0.23(0.10,0.81) | 9.15(4.28,21.78) | 0.25(0.12,0.61) | 11.32(-39.66,109.74) | 0.21(-0.06,0.48) |
| Republic of Sudan | 24.06(8.47,52.97) | 0.32(0.11,0.70) | 28.20(10.19,63.05) | 0.15(0.06,0.34) | -51.70(-74.22,-4.46) | -2.27(-2.46,-2.09) |
| Republic of Suriname | 0.84(0.52,1.05) | 0.51(0.32,0.65) | 0.57(0.41,0.77) | 0.27(0.19,0.36) | -48.32(-65.33,-13.29) | -1.83(-2.31,-1.36) |
| Republic of Tajikistan | 8.53(5.39,11.91) | 0.40(0.25,0.56) | 10.02(5.20,23.97) | 0.24(0.12,0.57) | -40.42(-71.29,34.13) | -2.43(-2.93,-1.93) |
| Republic of the Congo | 2.57(1.04,4.15) | 0.27(0.11,0.44) | 4.70(2.83,7.21) | 0.21(0.13,0.33) | -21.84(-54.59,78.86) | -0.72(-0.96,-0.49) |
| Republic of the Gambia | 1.54(0.71,4.25) | 0.41(0.19,1.13) | 2.35(1.32,3.67) | 0.23(0.13,0.37) | -42.60(-74.05,26.83) | -2.35(-2.71,-1.99) |
| Republic of the Marshall Islands | 0.07(0.03,0.11) | 0.43(0.20,0.65) | 0.06(0.03,0.10) | 0.27(0.12,0.43) | -35.91(-66.05,11.77) | -1.19(-1.30,-1.09) |
| Republic of the Niger | 11.26(5.07,40.32) | 0.40(0.18,1.45) | 18.33(8.91,51.48) | 0.21(0.10,0.58) | -49.20(-69.66,-9.31) | -2.68(-3.01,-2.35) |
| Republic of the Philippines | 107.45(69.58,141.26) | 0.41(0.27,0.54) | 119.80(90.03,146.98) | 0.25(0.19,0.31) | -38.84(-54.45,-15.64) | -1.42(-1.54,-1.30) |
| Republic of the Union of Myanmar | 225.42(110.78,380.21) | 1.31(0.65,2.21) | 108.45(61.79,190.08) | 0.48(0.27,0.85) | -63.26(-77.53,-32.85) | -3.63(-3.85,-3.41) |
| Republic of Trinidad and Tobago | 1.97(1.73,2.21) | 0.39(0.34,0.44) | 1.13(0.85,1.50) | 0.23(0.17,0.30) | -42.16(-57.17,-21.20) | -1.20(-1.58,-0.81) |
| Republic of Tunisia | 4.27(2.24,7.78) | 0.12(0.06,0.23) | 2.22(1.08,4.38) | 0.05(0.02,0.10) | -58.83(-75.79,-30.96) | -3.00(-3.09,-2.91) |
| Republic of Turkey | 27.33(13.26,47.42) | 0.11(0.06,0.20) | 7.35(5.18,10.30) | 0.02(0.02,0.03) | -79.80(-89.73,-45.76) | -5.50(-5.74,-5.25) |
| Republic of Uganda | 7.47(2.95,17.94) | 0.12(0.05,0.28) | 21.28(11.45,36.90) | 0.12(0.07,0.21) | 6.21(-43.75,125.19) | -0.46(-0.81,-0.10) |
| Republic of Uzbekistan | 24.50(20.33,29.72) | 0.29(0.24,0.35) | 9.49(7.08,12.37) | 0.07(0.05,0.09) | -75.80(-82.87,-66.86) | -5.38(-6.14,-4.61) |
| Republic of Vanuatu | 0.23(0.08,0.44) | 0.39(0.14,0.76) | 0.41(0.20,0.71) | 0.33(0.16,0.57) | -16.02(-59.24,74.07) | -0.77(-0.93,-0.62) |
| Republic of Yemen | 8.54(2.28,20.18) | 0.19(0.05,0.44) | 14.46(5.03,26.42) | 0.11(0.04,0.19) | -43.42(-73.75,16.02) | -2.13(-2.49,-1.76) |
| Republic of Zambia | 8.36(5.06,13.73) | 0.28(0.17,0.45) | 17.76(10.74,27.40) | 0.22(0.13,0.34) | -20.31(-57.62,37.47) | -0.85(-1.22,-0.47) |
| Republic of Zimbabwe | 9.10(5.13,21.33) | 0.23(0.13,0.54) | 21.69(11.89,37.97) | 0.34(0.19,0.60) | 49.06(-49.52,197.53) | 2.07(1.39,2.76) |
| Romania | 9.76(8.01,11.78) | 0.11(0.09,0.14) | 1.22(0.92,1.63) | 0.02(0.02,0.03) | -79.80(-85.94,-71.74) | -4.93(-5.34,-4.51) |
| Russian Federation | 101.57(94.95,108.68) | 0.17(0.16,0.19) | 22.82(21.19,24.48) | 0.05(0.05,0.05) | -71.88(-74.95,-69.03) | -4.33(-4.52,-4.13) |
| Saint Kitts and Nevis | 0.09(0.07,0.10) | 0.50(0.41,0.60) | 0.04(0.03,0.06) | 0.18(0.13,0.25) | -64.74(-75.14,-47.78) | -3.28(-4.39,-2.16) |
| Saint Lucia | 0.22(0.20,0.25) | 0.39(0.35,0.44) | 0.18(0.14,0.22) | 0.27(0.22,0.33) | -31.89(-46.16,-14.22) | -0.49(-1.15,0.17) |
| Saint Vincent and the Grenadines | 0.11(0.10,0.13) | 0.25(0.21,0.29) | 0.09(0.07,0.11) | 0.22(0.18,0.27) | -10.99(-30.41,12.61) | 0.27(-0.28,0.83) |
| Slovak Republic | 1.25(0.89,1.66) | 0.06(0.04,0.08) | 0.34(0.23,0.50) | 0.02(0.01,0.03) | -67.30(-78.62,-46.63) | -3.25(-3.50,-3.01) |
| Socialist Republic of Viet Nam | 74.59(41.94,129.43) | 0.26(0.15,0.45) | 37.16(20.63,61.70) | 0.10(0.05,0.16) | -62.99(-79.45,-31.08) | -3.35(-3.50,-3.20) |
| Solomon Islands | 0.29(0.10,0.56) | 0.22(0.08,0.44) | 0.50(0.25,0.81) | 0.18(0.09,0.30) | -18.69(-55.39,64.62) | -0.54(-0.68,-0.40) |
| State of Eritrea | 4.96(2.07,10.42) | 0.38(0.16,0.80) | 8.74(4.61,13.78) | 0.31(0.16,0.49) | -18.48(-52.23,44.19) | -0.66(-0.77,-0.55) |
| State of Israel | 0.42(0.38,0.48) | 0.02(0.02,0.03) | 0.23(0.20,0.27) | 0.01(0.01,0.01) | -68.92(-74.20,-62.47) | -3.53(-3.78,-3.28) |
| State of Kuwait | 0.28(0.22,0.35) | 0.03(0.03,0.04) | 0.24(0.19,0.30) | 0.01(0.01,0.01) | -65.02(-73.64,-52.72) | -2.80(-3.97,-1.62) |
| State of Libya | 3.18(1.53,6.42) | 0.19(0.09,0.38) | 2.72(1.35,4.28) | 0.09(0.05,0.14) | -52.02(-79.13,-7.06) | -2.20(-2.63,-1.78) |
| State of Qatar | 0.22(0.13,0.34) | 0.09(0.06,0.15) | 0.33(0.21,0.56) | 0.02(0.01,0.03) | -78.42(-88.29,-56.64) | -5.17(-5.57,-4.76) |
| Sultanate of Oman | 0.63(0.36,1.13) | 0.08(0.04,0.14) | 0.40(0.24,0.77) | 0.02(0.01,0.03) | -76.97(-86.92,-48.50) | -3.94(-4.18,-3.71) |
| Swiss Confederation | 1.18(1.05,1.33) | 0.04(0.04,0.05) | 0.27(0.23,0.32) | 0.01(0.01,0.01) | -78.28(-81.83,-73.36) | -5.13(-5.31,-4.96) |
| Syrian Arab Republic | 9.77(6.14,13.84) | 0.20(0.13,0.29) | 3.99(2.44,6.07) | 0.08(0.05,0.12) | -61.42(-78.32,-23.44) | -3.03(-3.30,-2.77) |
| Taiwan (Province of China) | 4.58(4.08,5.17) | 0.05(0.04,0.06) | 0.94(0.78,1.12) | 0.01(0.01,0.01) | -74.83(-80.12,-69.28) | -5.01(-5.45,-4.58) |
| Togolese Republic | 5.64(3.29,11.87) | 0.41(0.24,0.87) | 6.56(4.10,9.88) | 0.20(0.12,0.29) | -52.56(-77.01,-16.30) | -2.66(-2.89,-2.44) |
| Tokelau | 0.00(0.00,0.00) | 0.24(0.13,0.43) | 0.00(0.00,0.00) | 0.15(0.10,0.21) | -39.68(-66.90,18.82) | -2.32(-2.58,-2.06) |
| Turkmenistan | 5.37(4.50,6.29) | 0.35(0.29,0.41) | 2.17(1.68,2.85) | 0.10(0.08,0.14) | -70.23(-77.33,-59.36) | -4.91(-5.78,-4.04) |
| Tuvalu | 0.01(0.01,0.02) | 0.41(0.20,0.63) | 0.01(0.01,0.02) | 0.21(0.12,0.33) | -48.43(-71.11,-5.90) | -2.14(-2.19,-2.09) |
| Ukraine | 30.19(25.98,34.84) | 0.16(0.14,0.18) | 5.78(4.13,7.90) | 0.04(0.03,0.06) | -73.64(-81.68,-63.04) | -4.54(-5.23,-3.86) |
| Union of the Comoros | 0.45(0.18,1.20) | 0.26(0.11,0.70) | 0.63(0.35,1.16) | 0.20(0.11,0.38) | -20.74(-58.57,74.77) | -1.50(-2.25,-0.74) |
| United Arab Emirates | 0.49(0.23,0.79) | 0.05(0.02,0.08) | 0.81(0.49,1.31) | 0.02(0.01,0.03) | -60.66(-79.78,5.79) | -2.89(-3.16,-2.63) |
| United Kingdom of Great Britain and Northern Ireland | 7.14(7.00,7.31) | 0.03(0.03,0.03) | 4.73(4.40,5.02) | 0.02(0.02,0.02) | -36.40(-41.09,-31.74) | -1.44(-2.05,-0.83) |
| United Mexican States | 125.95(120.23,133.27) | 0.35(0.34,0.37) | 196.94(177.39,217.94) | 0.38(0.34,0.42) | 8.25(-4.13,21.39) | 0.80(0.41,1.19) |
| United Republic of Tanzania | 19.52(10.32,44.72) | 0.20(0.11,0.46) | 43.09(23.68,103.24) | 0.18(0.10,0.44) | -8.32(-45.82,68.29) | 0.01(-0.27,0.29) |
| United States of America | 40.70(39.25,42.30) | 0.04(0.04,0.04) | 34.51(32.53,36.40) | 0.03(0.03,0.03) | -22.17(-26.30,-17.82) | -0.84(-1.05,-0.63) |
| United States Virgin Islands | 0.17(0.12,0.23) | 0.42(0.31,0.57) | 0.08(0.05,0.13) | 0.32(0.21,0.55) | -22.72(-55.91,34.78) | -0.13(-0.42,0.17) |

AYAs = adolescents and young adults, PC = percentage change, EAPC = estimated annual percentage changes.
