# Supplementary material for: Global burden and trends of appendicitis among adolescents and young adults: A systematic analysis for the Global Burden of Disease study 2021 and predictions to 2040
Source: Medicine (Baltimore). 2026 Jul 3;105(27):e49625. doi: 10.1097/MD.0000000000049625 (PMC13336947; doi:10.1097/MD.0000000000049625)
Supplement: Supplementary file 9 [file medi-105-e49625-s009.docx]

S5 Table. DALY for appendicitis among AYAs by 204 countries or territories in 1990 and 2021, and PC from 1990 to 2021.

| Location | 1990 | | 2021 | |  |  |
| --- | --- | --- | --- | --- | --- | --- |
|  | DALY cases | DALY rate | DALY cases | DALY rate | Percentage change(95%UI) | EAPC |
| American Samoa | 3.46(2.49,4.78) | 17.08(12.30,23.63) | 2.29(1.63,3.17) | 13.13(9.31,18.15) | -23.13(-49.81,20.49) | -0.79(-1.08,-0.50) |
| Antigua and Barbuda | 5.50(4.68,6.37) | 21.31(18.13,24.70) | 5.07(3.99,6.61) | 14.74(11.60,19.20) | -30.84(-45.58,-10.62) | -0.13(-0.62,0.35) |
| Arab Republic of Egypt | 2130.38(1361.09,2750.26) | 9.72(6.21,12.55) | 2277.88(1416.62,3595.26) | 5.40(3.36,8.52) | -44.47(-64.88,-12.47) | -2.54(-2.96,-2.13) |
| Argentine Republic | 1696.06(1403.12,2069.11) | 13.88(11.49,16.94) | 1748.63(1343.10,2302.49) | 9.98(7.66,13.14) | -28.13(-44.85,-8.17) | -0.62(-0.94,-0.29) |
| Australia | 501.79(312.88,769.41) | 7.41(4.62,11.36) | 555.71(318.54,883.15) | 6.41(3.67,10.19) | -13.49(-38.91,22.93) | -0.47(-0.51,-0.44) |
| Barbados | 21.67(18.56,25.08) | 19.86(17.01,22.98) | 11.37(8.58,15.29) | 11.51(8.68,15.48) | -42.05(-56.93,-24.99) | -1.68(-1.95,-1.40) |
| Belize | 13.70(11.76,15.85) | 18.73(16.08,21.66) | 35.16(29.26,41.83) | 18.63(15.50,22.16) | -0.53(-21.69,20.43) | 0.71(0.43,0.98) |
| Bermuda | 4.14(3.45,4.94) | 16.10(13.40,19.21) | 1.81(1.35,2.50) | 10.37(7.72,14.28) | -35.60(-50.97,-13.69) | -0.84(-1.44,-0.23) |
| Bolivarian Republic of Venezuela | 3106.74(2776.66,3469.08) | 38.79(34.67,43.31) | 2633.75(1883.68,3499.74) | 28.13(20.12,37.38) | -27.48(-48.49,-3.72) | -0.41(-0.81,-0.01) |
| Bosnia and Herzegovina | 199.89(148.16,264.03) | 10.53(7.80,13.90) | 49.68(30.65,72.84) | 4.94(3.05,7.24) | -53.09(-71.31,-32.08) | -2.97(-3.30,-2.65) |
| Brunei Darussalam | 14.84(10.52,20.39) | 12.04(8.53,16.54) | 18.14(11.43,27.26) | 8.89(5.60,13.36) | -26.15(-48.18,8.16) | -0.89(-1.00,-0.78) |
| Burkina Faso | 643.32(337.10,1633.10) | 20.18(10.57,51.23) | 1056.15(696.41,1885.64) | 12.20(8.05,21.79) | -39.53(-63.61,-1.92) | -1.85(-2.21,-1.48) |
| Canada | 674.82(420.97,1025.24) | 6.07(3.79,9.22) | 658.42(392.78,1007.01) | 5.55(3.31,8.49) | -8.56(-37.04,31.31) | -0.28(-0.34,-0.21) |
| Central African Republic | 299.68(121.34,520.42) | 28.78(11.65,49.98) | 649.32(277.17,1140.62) | 29.74(12.70,52.24) | 3.35(-33.76,71.36) | 0.18(0.07,0.29) |
| Commonwealth of Dominica | 5.93(4.71,7.27) | 20.29(16.12,24.90) | 4.23(3.04,5.58) | 16.32(11.72,21.54) | -19.56(-44.88,13.68) | -0.13(-0.58,0.32) |
| Commonwealth of the Bahamas | 28.19(23.87,32.93) | 23.88(20.23,27.90) | 31.43(24.49,39.94) | 20.32(15.83,25.82) | -14.92(-38.73,10.13) | 0.20(-0.15,0.55) |
| Cook Islands | 0.48(0.33,0.65) | 6.18(4.31,8.39) | 0.25(0.17,0.37) | 4.31(2.86,6.34) | -30.27(-54.56,1.74) | -0.98(-1.23,-0.72) |
| Czech Republic | 360.69(283.97,464.88) | 9.72(7.65,12.53) | 185.81(115.95,269.70) | 6.29(3.93,9.13) | -35.27(-55.53,-10.69) | -1.15(-1.31,-0.98) |
| Democratic People's Republic of Korea | 745.70(460.38,1067.02) | 8.94(5.52,12.79) | 673.33(406.55,1011.45) | 6.69(4.04,10.06) | -25.12(-54.46,23.12) | -1.05(-1.09,-1.00) |
| Democratic Republic of Sao Tome and Principe | 5.74(2.37,13.83) | 13.36(5.52,32.21) | 6.04(3.65,9.34) | 6.65(4.01,10.28) | -50.25(-80.50,48.19) | -2.74(-3.11,-2.36) |
| Democratic Republic of the Congo | 3005.76(1434.26,5458.45) | 20.98(10.01,38.10) | 6776.23(3708.82,12832.60) | 18.78(10.28,35.56) | -10.51(-45.49,55.28) | -0.19(-0.29,-0.09) |
| Democratic Republic of Timor-Leste | 120.04(59.52,299.50) | 37.71(18.70,94.09) | 134.58(80.91,271.46) | 23.55(14.16,47.51) | -37.54(-61.80,8.50) | -1.69(-2.33,-1.04) |
| Democratic Socialist Republic of Sri Lanka | 1018.13(747.43,1318.16) | 13.77(10.11,17.83) | 393.79(265.88,549.40) | 4.88(3.30,6.81) | -64.54(-77.13,-45.80) | -3.87(-4.17,-3.57) |
| Dominican Republic | 1338.67(936.96,1682.10) | 43.58(30.50,54.76) | 1127.49(605.22,1617.36) | 24.79(13.31,35.57) | -43.10(-64.71,-19.09) | -1.07(-1.55,-0.58) |
| Eastern Republic of Uruguay | 167.75(140.86,196.43) | 14.77(12.40,17.29) | 137.26(107.07,176.08) | 11.47(8.95,14.72) | -22.30(-36.82,-4.63) | -0.61(-0.87,-0.35) |
| Federal Democratic Republic of Ethiopia | 3704.34(1551.74,7872.51) | 20.28(8.49,43.09) | 5370.83(3057.80,15726.95) | 11.59(6.60,33.93) | -42.86(-66.47,34.86) | -2.18(-2.34,-2.03) |
| Federal Democratic Republic of Nepal | 10625.78(5624.44,24299.71) | 145.46(76.99,332.64) | 5842.37(3711.82,9753.06) | 43.57(27.68,72.74) | -70.04(-83.00,-43.63) | -4.14(-4.29,-3.99) |
| Federal Republic of Germany | 2135.74(1484.58,3058.87) | 7.19(5.00,10.30) | 1349.85(760.85,2266.80) | 5.34(3.01,8.96) | -25.78(-53.84,8.81) | -0.89(-0.98,-0.79) |
| Federal Republic of Nigeria | 5112.72(2703.26,9540.94) | 14.97(7.92,27.94) | 6490.71(3923.47,10213.03) | 7.22(4.36,11.36) | -51.80(-70.10,-29.46) | -2.79(-3.03,-2.54) |
| Federal Republic of Somalia | 652.02(268.30,1620.41) | 22.49(9.25,55.89) | 1753.21(692.24,4328.23) | 21.15(8.35,52.21) | -5.97(-42.97,51.65) | -0.24(-0.32,-0.15) |
| Federated States of Micronesia | 10.89(5.48,16.36) | 27.17(13.68,40.82) | 7.42(3.99,10.86) | 17.47(9.41,25.58) | -35.69(-60.91,11.34) | -1.39(-1.49,-1.28) |
| Federative Republic of Brazil | 14070.68(12879.48,15596.95) | 22.42(20.53,24.86) | 17634.31(16071.07,19397.49) | 20.69(18.85,22.75) | -7.76(-14.34,-0.36) | -0.02(-0.14,0.11) |
| French Republic | 1509.13(1030.05,2142.88) | 6.86(4.68,9.74) | 1079.63(582.95,1755.05) | 5.43(2.93,8.83) | -20.78(-49.01,10.18) | -0.69(-0.78,-0.60) |
| Gabonese Republic | 46.92(24.57,72.88) | 12.20(6.39,18.95) | 80.31(46.23,126.36) | 10.71(6.17,16.86) | -12.16(-47.08,61.37) | -0.40(-0.56,-0.24) |
| Georgia | 272.91(231.47,323.86) | 12.82(10.87,15.21) | 69.78(47.65,100.34) | 6.15(4.20,8.84) | -52.03(-66.02,-35.08) | -2.98(-3.65,-2.29) |
| Grand Duchy of Luxembourg | 11.13(7.72,15.78) | 7.54(5.23,10.69) | 13.51(8.21,19.06) | 6.13(3.72,8.64) | -18.76(-46.07,19.27) | -0.65(-0.72,-0.58) |
| Greenland | 1.66(1.01,2.55) | 6.26(3.81,9.62) | 1.40(0.81,2.12) | 6.86(3.98,10.39) | 9.63(-43.18,76.50) | 0.51(0.36,0.66) |
| Grenada | 7.15(6.11,8.35) | 21.45(18.32,25.04) | 5.36(4.14,6.70) | 13.26(10.24,16.58) | -38.20(-52.44,-22.41) | -0.82(-1.39,-0.24) |
| Guam | 3.55(2.64,4.70) | 5.59(4.16,7.41) | 3.10(2.31,4.08) | 5.60(4.17,7.35) | 0.19(-27.00,31.66) | 0.32(0.14,0.50) |
| Hashemite Kingdom of Jordan | 135.52(90.61,192.59) | 8.82(5.90,12.53) | 333.71(197.37,528.99) | 6.22(3.68,9.85) | -29.50(-54.15,5.59) | -1.36(-1.49,-1.23) |
| Hellenic Republic | 154.88(92.79,255.99) | 4.12(2.47,6.81) | 104.76(55.59,178.27) | 3.76(2.00,6.40) | -8.66(-40.83,38.54) | -0.26(-0.32,-0.19) |
| Hungary | 491.27(400.40,600.00) | 13.29(10.83,16.23) | 146.08(88.48,214.17) | 5.31(3.22,7.78) | -60.06(-73.10,-45.39) | -2.77(-3.14,-2.40) |
| Independent State of Papua New Guinea | 187.58(78.19,504.80) | 11.33(4.72,30.50) | 354.87(216.43,700.83) | 8.29(5.06,16.38) | -26.83(-55.63,32.78) | -1.28(-1.47,-1.10) |
| Independent State of Samoa | 8.58(5.05,12.38) | 12.81(7.55,18.49) | 8.20(5.08,12.30) | 10.21(6.32,15.31) | -20.32(-50.22,22.58) | -0.73(-0.84,-0.62) |
| Ireland | 78.71(49.21,122.35) | 5.74(3.59,8.92) | 77.04(40.28,124.62) | 4.92(2.57,7.96) | -14.19(-45.82,27.22) | -0.60(-0.67,-0.53) |
| Islamic Republic of Afghanistan | 1334.01(510.68,2402.08) | 42.36(16.21,76.27) | 2992.76(1522.19,4796.20) | 24.49(12.46,39.25) | -42.18(-67.06,1.95) | -1.96(-2.19,-1.74) |
| Islamic Republic of Iran | 3981.22(3059.99,5626.48) | 18.33(14.09,25.91) | 2739.65(2056.22,3724.64) | 7.89(5.93,10.73) | -56.93(-70.91,-40.76) | -2.72(-2.83,-2.62) |
| Islamic Republic of Mauritania | 153.93(82.31,240.28) | 20.02(10.71,31.25) | 142.70(94.82,241.19) | 8.36(5.55,14.13) | -58.25(-74.51,-23.91) | -3.11(-3.24,-2.97) |
| Islamic Republic of Pakistan | 33804.66(16961.23,78173.22) | 82.84(41.57,191.58) | 52667.10(34788.56,74242.27) | 53.25(35.17,75.06) | -35.72(-70.98,18.95) | -2.08(-2.35,-1.81) |
| Jamaica | 146.71(124.58,175.60) | 14.93(12.68,17.87) | 146.62(106.94,197.06) | 12.29(8.96,16.51) | -17.69(-38.57,8.42) | -0.92(-1.24,-0.60) |
| Japan | 4322.00(2701.20,6553.40) | 9.64(6.03,14.62) | 2609.70(1637.19,3820.93) | 8.05(5.05,11.79) | -16.51(-25.75,-5.11) | -0.62(-0.75,-0.49) |
| Kingdom of Bahrain | 23.07(16.54,31.54) | 9.00(6.45,12.30) | 49.85(31.89,74.38) | 7.08(4.53,10.57) | -21.28(-49.89,15.27) | -0.77(-0.98,-0.55) |
| Kingdom of Belgium | 262.28(165.50,398.02) | 7.05(4.45,10.70) | 241.38(145.60,363.17) | 6.89(4.16,10.37) | -2.24(-34.20,59.82) | -0.25(-0.36,-0.14) |
| Kingdom of Bhutan | 296.84(132.91,651.25) | 110.16(49.32,241.69) | 127.69(69.68,285.98) | 36.84(20.11,82.51) | -66.56(-80.45,-31.08) | -3.88(-4.00,-3.75) |
| Kingdom of Cambodia | 3548.23(1764.46,5721.11) | 92.15(45.83,148.59) | 3113.61(1734.59,5365.57) | 42.99(23.95,74.08) | -53.35(-73.29,-9.55) | -2.73(-2.90,-2.55) |
| Kingdom of Denmark | 167.41(123.23,238.44) | 8.77(6.46,12.50) | 112.33(67.44,182.37) | 6.16(3.70,10.00) | -29.80(-53.90,-1.67) | -1.24(-1.38,-1.10) |
| Kingdom of Eswatini | 89.07(55.62,150.42) | 29.55(18.46,49.91) | 114.43(68.89,176.80) | 22.48(13.53,34.73) | -23.95(-65.97,32.36) | -0.71(-1.25,-0.18) |
| Kingdom of Lesotho | 145.28(82.11,376.47) | 26.92(15.22,69.77) | 230.17(139.24,347.23) | 27.67(16.74,41.74) | 2.77(-66.90,96.23) | 0.76(0.32,1.20) |
| Kingdom of Morocco | 1960.96(1156.37,3242.96) | 18.86(11.12,31.19) | 1399.14(914.60,2186.27) | 9.53(6.23,14.89) | -49.47(-67.89,-18.62) | -2.35(-2.54,-2.16) |
| Kingdom of Norway | 134.63(91.26,194.31) | 8.42(5.70,12.15) | 113.71(71.35,164.09) | 6.41(4.02,9.25) | -23.86(-35.88,-9.81) | -0.86(-0.94,-0.79) |
| Kingdom of Saudi Arabia | 607.37(370.96,994.97) | 9.14(5.58,14.97) | 1192.76(742.03,1885.15) | 6.44(4.01,10.18) | -29.54(-59.71,19.17) | -0.93(-1.06,-0.79) |
| Kingdom of Spain | 1071.56(746.35,1549.17) | 7.23(5.03,10.45) | 641.51(365.63,1107.95) | 5.17(2.94,8.92) | -28.49(-54.90,4.86) | -1.14(-1.28,-1.00) |
| Kingdom of Sweden | 220.12(135.46,345.81) | 7.50(4.61,11.78) | 210.17(112.33,337.21) | 6.48(3.47,10.40) | -13.54(-39.31,21.46) | -0.31(-0.40,-0.23) |
| Kingdom of Thailand | 4402.12(2899.94,6731.07) | 16.98(11.18,25.96) | 2016.58(1271.67,2869.50) | 9.51(6.00,13.54) | -43.96(-66.17,-18.24) | -2.73(-3.11,-2.35) |
| Kingdom of the Netherlands | 421.32(281.42,594.88) | 6.99(4.67,9.87) | 284.73(157.35,462.09) | 5.39(2.98,8.75) | -22.83(-48.98,8.93) | -0.79(-0.89,-0.69) |
| Kingdom of Tonga | 4.81(3.22,9.22) | 13.03(8.72,24.99) | 4.69(3.00,6.80) | 12.05(7.71,17.49) | -7.55(-50.49,52.56) | -0.02(-0.21,0.17) |
| Kyrgyz Republic | 502.37(423.20,594.63) | 27.85(23.46,32.97) | 234.79(177.77,318.23) | 8.63(6.53,11.69) | -69.03(-77.97,-58.42) | -4.45(-5.17,-3.73) |
| Lao People's Democratic Republic | 737.42(354.36,1144.20) | 47.73(22.94,74.06) | 692.58(401.83,1213.10) | 21.59(12.53,37.82) | -54.77(-73.63,-16.56) | -2.73(-2.85,-2.61) |
| Lebanese Republic | 84.30(50.69,122.80) | 7.31(4.40,10.65) | 127.91(75.15,209.11) | 5.51(3.24,9.01) | -24.61(-55.35,28.15) | -0.83(-0.98,-0.68) |
| Malaysia | 901.39(660.02,1313.14) | 12.14(8.89,17.69) | 1037.14(699.59,1550.96) | 7.46(5.03,11.16) | -38.57(-55.07,-18.44) | -2.13(-2.38,-1.88) |
| Mongolia | 313.59(190.05,499.57) | 35.49(21.51,56.53) | 213.27(157.37,283.49) | 16.90(12.47,22.46) | -52.38(-71.58,-18.98) | -3.23(-3.50,-2.96) |
| Montenegro | 13.35(7.86,20.45) | 5.32(3.13,8.15) | 9.54(5.13,15.94) | 4.64(2.49,7.75) | -12.81(-42.54,33.57) | -0.54(-0.67,-0.41) |
| New Zealand | 111.22(73.51,160.14) | 8.05(5.32,11.59) | 117.72(74.76,171.20) | 6.53(4.15,9.50) | -18.83(-40.25,13.00) | -0.50(-0.62,-0.38) |
| North Macedonia | 45.10(29.16,65.73) | 5.68(3.67,8.28) | 32.90(18.78,54.56) | 4.30(2.46,7.13) | -24.26(-50.35,11.48) | -0.91(-0.96,-0.86) |
| Northern Mariana Islands | 1.32(0.85,1.88) | 5.62(3.62,8.03) | 0.85(0.55,1.21) | 5.16(3.33,7.32) | -8.11(-37.22,36.99) | -0.28(-0.64,0.08) |
| Palestine | 110.36(55.98,250.54) | 14.38(7.29,32.64) | 153.03(87.46,262.73) | 7.01(4.01,12.03) | -51.26(-73.56,-9.17) | -2.37(-2.59,-2.16) |
| People's Democratic Republic of Algeria | 1548.32(1080.78,2188.09) | 15.32(10.70,21.65) | 1365.43(923.41,2068.47) | 8.02(5.42,12.15) | -47.67(-67.13,-17.64) | -2.27(-2.38,-2.16) |
| People's Republic of Bangladesh | 44520.70(27069.55,72182.84) | 105.47(64.13,171.01) | 26279.96(16604.43,45408.84) | 38.19(24.13,65.99) | -63.79(-77.93,-28.75) | -3.26(-3.44,-3.09) |
| People's Republic of China | 68705.54(51432.93,87392.00) | 12.53(9.38,15.94) | 33249.13(20978.27,49467.33) | 7.21(4.55,10.72) | -42.51(-58.25,-24.69) | -1.78(-1.90,-1.65) |
| Plurinational State of Bolivia | 2995.23(1484.37,4722.69) | 121.46(60.19,191.51) | 2009.46(1332.43,3095.15) | 40.89(27.12,62.99) | -66.33(-80.36,-35.86) | -3.79(-4.01,-3.56) |
| Portuguese Republic | 237.64(173.88,324.48) | 6.28(4.59,8.57) | 133.08(84.53,205.15) | 4.51(2.86,6.95) | -28.17(-49.21,3.07) | -1.01(-1.11,-0.91) |
| Principality of Andorra | 1.37(0.80,2.07) | 5.49(3.20,8.27) | 1.29(0.69,2.10) | 5.07(2.72,8.23) | -7.69(-38.21,42.32) | -0.31(-0.37,-0.25) |
| Principality of Monaco | 0.45(0.25,0.75) | 4.93(2.72,8.17) | 0.46(0.26,0.74) | 4.89(2.83,7.99) | -0.84(-38.01,46.43) | 0.05(0.02,0.08) |
| Puerto Rico | 177.02(149.50,216.85) | 12.51(10.57,15.33) | 81.08(57.35,112.67) | 7.84(5.54,10.89) | -37.35(-53.61,-18.59) | -1.21(-1.58,-0.84) |
| Republic of Albania | 105.22(71.55,147.88) | 7.41(5.04,10.41) | 44.14(23.89,71.44) | 4.66(2.52,7.54) | -37.15(-63.11,-2.81) | -1.42(-1.50,-1.34) |
| Republic of Angola | 805.02(312.85,1441.60) | 20.57(8.00,36.84) | 1899.62(1065.50,3085.49) | 15.61(8.76,25.36) | -24.11(-54.78,40.68) | -0.67(-0.86,-0.48) |
| Republic of Armenia | 285.21(233.21,339.50) | 19.84(16.23,23.62) | 68.38(46.99,99.33) | 6.36(4.37,9.24) | -67.95(-78.29,-55.44) | -4.29(-5.09,-3.49) |
| Republic of Austria | 214.12(143.37,316.54) | 7.13(4.78,10.54) | 164.00(96.78,250.36) | 5.81(3.43,8.87) | -18.54(-45.65,18.45) | -0.45(-0.59,-0.32) |
| Republic of Azerbaijan | 803.09(616.11,1004.67) | 25.27(19.39,31.61) | 301.48(200.28,460.53) | 7.12(4.73,10.87) | -71.83(-81.18,-56.72) | -4.86(-5.58,-4.14) |
| Republic of Belarus | 365.72(283.98,471.46) | 9.27(7.20,11.95) | 164.28(108.54,242.05) | 5.60(3.70,8.25) | -39.60(-58.75,-15.16) | -1.53(-1.67,-1.38) |
| Republic of Benin | 408.14(221.96,903.04) | 23.97(13.03,53.03) | 634.87(406.12,1072.06) | 12.11(7.75,20.45) | -49.48(-70.30,-11.49) | -2.61(-2.86,-2.36) |
| Republic of Botswana | 174.60(96.53,370.34) | 33.92(18.75,71.94) | 137.31(81.75,243.10) | 12.89(7.67,22.82) | -62.00(-78.12,-38.88) | -3.23(-3.45,-3.02) |
| Republic of Bulgaria | 340.78(276.68,423.83) | 11.45(9.29,14.24) | 113.75(76.94,166.03) | 5.99(4.05,8.74) | -47.69(-62.98,-27.78) | -1.90(-2.14,-1.65) |
| Republic of Burundi | 448.49(177.35,1023.48) | 21.63(8.56,49.37) | 878.00(415.92,2126.11) | 16.65(7.89,40.33) | -23.03(-52.16,39.36) | -1.19(-1.33,-1.06) |
| Republic of Cabo Verde | 7.27(5.22,10.86) | 5.56(3.99,8.31) | 9.23(6.53,13.27) | 3.68(2.61,5.30) | -33.75(-55.12,-5.66) | -1.58(-1.70,-1.46) |
| Republic of Cameroon | 736.37(429.37,1153.67) | 19.36(11.29,30.33) | 1349.37(781.83,2046.80) | 10.47(6.06,15.88) | -45.94(-67.35,-12.95) | -2.42(-2.66,-2.18) |
| Republic of Chad | 452.79(243.13,1358.60) | 21.57(11.58,64.72) | 969.68(620.89,1647.27) | 15.41(9.87,26.18) | -28.57(-60.72,32.16) | -1.38(-1.63,-1.13) |
| Republic of Chile | 1028.29(880.20,1213.10) | 17.95(15.37,21.18) | 754.46(572.35,990.62) | 10.66(8.09,14.00) | -40.61(-53.66,-24.88) | -1.54(-1.83,-1.25) |
| Republic of Colombia | 3755.02(3384.12,4165.11) | 26.72(24.08,29.64) | 3879.12(3176.30,4600.15) | 19.30(15.81,22.89) | -27.75(-40.56,-11.63) | -0.58(-0.90,-0.27) |
| Republic of Costa Rica | 193.22(163.88,231.19) | 15.04(12.76,18.00) | 319.35(258.04,398.57) | 16.78(13.56,20.94) | 11.55(-9.85,38.83) | 0.31(-0.01,0.64) |
| Republic of Croatia | 171.84(130.68,221.87) | 9.47(7.20,12.23) | 61.85(40.82,86.96) | 4.96(3.27,6.97) | -47.67(-64.34,-26.82) | -1.88(-2.07,-1.70) |
| Republic of Cuba | 1471.87(1309.81,1654.69) | 30.16(26.84,33.91) | 690.65(559.01,837.97) | 19.26(15.59,23.37) | -36.14(-47.59,-22.50) | -0.94(-1.56,-0.31) |
| Republic of Cyprus | 12.71(7.66,19.10) | 4.13(2.49,6.21) | 15.37(9.11,24.03) | 3.06(1.82,4.79) | -25.90(-50.29,7.32) | -1.30(-1.44,-1.15) |
| Republic of Côte d'Ivoire | 949.64(497.43,1670.37) | 20.08(10.52,35.31) | 1266.32(826.22,1791.91) | 11.29(7.37,15.98) | -43.74(-65.98,-1.00) | -2.10(-2.34,-1.86) |
| Republic of Djibouti | 27.31(14.94,85.03) | 15.58(8.52,48.50) | 69.58(40.58,134.33) | 12.87(7.50,24.84) | -17.41(-51.74,36.98) | -0.74(-1.07,-0.41) |
| Republic of Ecuador | 4873.18(4391.50,5434.54) | 118.10(106.43,131.70) | 2051.95(1620.68,2545.62) | 28.09(22.19,34.85) | -76.21(-81.54,-69.67) | -4.13(-4.57,-3.70) |
| Republic of El Salvador | 1108.53(885.07,1355.96) | 53.06(42.36,64.90) | 662.50(482.93,870.50) | 25.56(18.63,33.58) | -51.83(-66.86,-33.77) | -2.00(-2.39,-1.61) |
| Republic of Equatorial Guinea | 47.27(20.93,86.02) | 31.30(13.86,56.97) | 74.71(44.37,120.62) | 10.74(6.38,17.34) | -65.69(-83.12,-13.81) | -4.02(-4.34,-3.71) |
| Republic of Estonia | 65.32(50.77,80.76) | 11.50(8.94,14.22) | 18.50(11.24,28.96) | 4.68(2.84,7.32) | -59.31(-75.10,-40.79) | -2.73(-3.14,-2.31) |
| Republic of Fiji | 58.88(43.14,80.63) | 18.26(13.38,25.00) | 52.69(36.62,72.90) | 14.77(10.26,20.44) | -19.11(-48.09,31.97) | -0.08(-0.46,0.30) |
| Republic of Finland | 159.45(109.44,224.61) | 8.78(6.03,12.37) | 106.82(66.87,164.16) | 6.41(4.01,9.85) | -27.00(-49.67,5.39) | -0.85(-0.93,-0.78) |
| Republic of Ghana | 1719.62(1182.89,2312.74) | 29.96(20.61,40.29) | 2802.45(1872.71,4625.32) | 19.60(13.09,32.34) | -34.59(-59.39,14.15) | -1.29(-1.44,-1.13) |
| Republic of Guatemala | 3846.14(3475.72,4217.68) | 130.20(117.66,142.78) | 3690.64(3098.51,4422.88) | 54.22(45.52,64.98) | -58.36(-66.45,-48.35) | -2.76(-3.20,-2.31) |
| Republic of Guinea | 415.91(227.93,911.21) | 20.24(11.09,44.35) | 667.06(414.88,990.99) | 12.91(8.03,19.18) | -36.23(-67.80,7.32) | -1.77(-1.97,-1.58) |
| Republic of Guinea-Bissau | 152.59(69.17,230.24) | 41.15(18.65,62.09) | 177.32(103.94,267.44) | 21.02(12.32,31.70) | -48.93(-69.72,-9.85) | -2.36(-2.49,-2.24) |
| Republic of Guyana | 220.32(179.19,260.36) | 64.73(52.65,76.50) | 145.98(111.15,188.76) | 46.98(35.77,60.75) | -27.42(-45.81,-2.92) | 0.10(-0.29,0.49) |
| Republic of Haiti | 1580.70(684.85,2773.61) | 64.93(28.13,113.94) | 2269.32(1151.32,3700.83) | 41.34(20.97,67.42) | -36.34(-58.42,-1.77) | -1.09(-1.33,-0.86) |
| Republic of Honduras | 1400.68(855.77,2067.47) | 81.13(49.57,119.75) | 1599.05(898.76,2547.59) | 36.38(20.45,57.96) | -55.16(-74.28,-25.82) | -2.84(-2.99,-2.69) |
| Republic of Iceland | 5.93(3.71,8.90) | 5.71(3.57,8.57) | 7.15(4.07,11.68) | 5.97(3.40,9.76) | 4.71(-30.99,55.85) | 0.35(0.18,0.52) |
| Republic of India | 435456.67(335857.25,548460.40) | 127.69(98.48,160.83) | 214229.46(161176.81,279971.18) | 35.15(26.45,45.94) | -72.47(-81.39,-60.66) | -4.59(-5.00,-4.17) |
| Republic of Indonesia | 34783.87(22172.44,50070.66) | 44.57(28.41,64.16) | 23086.45(15799.26,30861.05) | 20.27(13.87,27.10) | -54.51(-67.70,-36.12) | -2.53(-2.64,-2.42) |
| Republic of Iraq | 426.50(281.30,614.78) | 5.94(3.91,8.56) | 897.62(506.25,1450.54) | 5.15(2.90,8.32) | -13.27(-45.42,31.06) | -0.60(-0.69,-0.52) |
| Republic of Italy | 1351.59(911.58,1973.83) | 6.33(4.27,9.25) | 740.62(496.82,1041.56) | 4.69(3.15,6.59) | -25.94(-37.99,-11.57) | -1.28(-1.45,-1.10) |
| Republic of Kazakhstan | 1390.42(1151.15,1688.25) | 20.48(16.96,24.87) | 581.42(414.43,819.31) | 8.34(5.95,11.76) | -59.27(-71.45,-44.10) | -4.04(-4.55,-3.52) |
| Republic of Kenya | 1157.04(703.67,3657.64) | 13.21(8.03,41.77) | 2658.22(1659.36,5861.55) | 12.28(7.66,27.07) | -7.07(-38.35,30.62) | 0.17(-0.09,0.43) |
| Republic of Kiribati | 8.20(3.22,13.10) | 26.86(10.56,42.91) | 10.47(5.06,17.05) | 21.05(10.19,34.28) | -21.62(-54.23,30.97) | -0.98(-1.08,-0.87) |
| Republic of Korea | 3688.42(2629.90,4769.88) | 17.52(12.49,22.66) | 1333.40(775.26,2029.44) | 8.33(4.84,12.68) | -52.45(-70.78,-31.41) | -1.95(-2.35,-1.54) |
| Republic of Latvia | 124.97(98.17,154.50) | 13.10(10.29,16.20) | 28.89(18.30,44.50) | 5.36(3.40,8.26) | -59.06(-72.85,-39.47) | -2.90(-3.33,-2.46) |
| Republic of Liberia | 179.66(94.51,319.57) | 19.47(10.24,34.64) | 248.54(159.67,424.51) | 11.07(7.11,18.91) | -43.16(-66.36,5.90) | -2.10(-2.38,-1.82) |
| Republic of Lithuania | 182.71(149.19,231.71) | 13.11(10.71,16.63) | 44.16(28.58,66.19) | 5.49(3.55,8.22) | -58.17(-71.51,-40.97) | -2.49(-2.87,-2.10) |
| Republic of Madagascar | 933.64(486.20,2009.51) | 20.61(10.73,44.36) | 2243.84(1246.16,5091.16) | 19.16(10.64,43.47) | -7.04(-41.99,52.85) | -0.20(-0.30,-0.10) |
| Republic of Malawi | 804.05(408.58,2012.66) | 21.52(10.94,53.87) | 1445.86(871.03,2764.43) | 17.67(10.64,33.78) | -17.89(-49.03,30.56) | -0.62(-0.83,-0.41) |
| Republic of Maldives | 5.99(3.45,8.63) | 7.38(4.24,10.62) | 9.17(6.15,13.74) | 3.53(2.36,5.28) | -52.21(-70.98,-16.46) | -2.34(-2.50,-2.18) |
| Republic of Mali | 986.49(535.01,2191.51) | 33.04(17.92,73.40) | 1564.27(989.99,2787.82) | 17.56(11.11,31.30) | -46.85(-66.96,-11.61) | -2.20(-2.37,-2.02) |
| Republic of Malta | 6.89(4.31,10.60) | 4.99(3.12,7.68) | 6.45(4.06,9.60) | 4.82(3.04,7.17) | -3.50(-35.93,52.69) | 0.08(-0.10,0.27) |
| Republic of Mauritius | 35.96(29.44,44.33) | 7.23(5.92,8.92) | 22.58(16.81,30.06) | 4.96(3.69,6.60) | -31.43(-44.43,-17.54) | -1.75(-2.05,-1.46) |
| Republic of Moldova | 244.05(200.37,295.81) | 14.00(11.50,16.97) | 67.88(45.04,98.67) | 5.47(3.63,7.96) | -60.91(-73.13,-46.68) | -2.87(-3.39,-2.36) |
| Republic of Mozambique | 984.71(474.89,2962.62) | 20.76(10.01,62.46) | 3199.98(1685.69,6031.29) | 26.61(14.02,50.16) | 28.19(-24.99,129.07) | 1.54(1.29,1.79) |
| Republic of Namibia | 168.50(94.39,372.28) | 30.13(16.88,66.57) | 164.72(96.34,279.86) | 15.76(9.22,26.78) | -47.69(-70.00,-9.03) | -2.38(-2.67,-2.09) |
| Republic of Nauru | 0.97(0.35,1.56) | 24.03(8.61,38.63) | 0.81(0.32,1.26) | 17.46(6.95,26.99) | -27.35(-54.57,15.20) | -1.07(-1.50,-0.65) |
| Republic of Nicaragua | 426.90(330.16,529.61) | 28.92(22.37,35.88) | 507.03(352.37,659.57) | 17.81(12.38,23.17) | -38.40(-56.43,-14.82) | -1.26(-1.49,-1.03) |
| Republic of Niue | 0.11(0.07,0.17) | 14.07(8.88,21.67) | 0.06(0.04,0.08) | 9.95(6.90,13.88) | -29.29(-57.30,22.49) | -1.56(-1.69,-1.42) |
| Republic of Palau | 1.00(0.47,1.57) | 14.32(6.80,22.55) | 0.79(0.41,1.21) | 13.48(7.02,20.59) | -5.92(-45.82,77.17) | -0.20(-0.25,-0.15) |
| Republic of Panama | 217.14(184.67,252.99) | 21.47(18.26,25.01) | 286.93(232.29,357.99) | 17.38(14.07,21.69) | -19.01(-35.89,2.31) | -0.14(-0.45,0.18) |
| Republic of Paraguay | 466.58(358.31,596.12) | 29.77(22.86,38.04) | 698.78(513.44,935.05) | 22.84(16.78,30.56) | -23.28(-46.11,10.13) | -0.35(-0.57,-0.13) |
| Republic of Peru | 12668.36(9251.70,15852.20) | 142.80(104.29,178.69) | 4194.67(3123.99,5701.00) | 28.23(21.02,38.36) | -80.23(-86.62,-67.84) | -5.51(-6.17,-4.83) |
| Republic of Poland | 1567.16(1263.85,1996.37) | 10.85(8.75,13.82) | 669.48(475.43,887.69) | 5.53(3.93,7.34) | -49.00(-57.54,-39.23) | -2.22(-2.41,-2.03) |
| Republic of Rwanda | 655.21(249.46,1054.01) | 23.91(9.10,38.46) | 816.07(441.89,2316.41) | 14.39(7.79,40.84) | -39.81(-69.73,56.91) | -2.27(-2.54,-2.00) |
| Republic of San Marino | 0.51(0.30,0.81) | 5.39(3.20,8.63) | 0.46(0.24,0.75) | 5.10(2.63,8.34) | -5.39(-41.71,49.62) | -0.10(-0.19,0.00) |
| Republic of Senegal | 837.46(424.53,2215.66) | 30.35(15.38,80.29) | 818.75(554.44,1242.64) | 12.70(8.60,19.27) | -58.16(-76.58,-7.28) | -2.88(-3.08,-2.68) |
| Republic of Serbia | 272.32(192.42,365.09) | 7.58(5.36,10.17) | 141.45(93.35,201.37) | 4.77(3.15,6.79) | -37.07(-57.99,-8.04) | -1.53(-1.62,-1.44) |
| Republic of Seychelles | 11.23(9.08,13.85) | 35.96(29.08,44.35) | 6.09(4.71,8.19) | 15.87(12.28,21.35) | -55.88(-66.52,-40.33) | -2.28(-2.59,-1.98) |
| Republic of Sierra Leone | 296.47(137.43,648.22) | 18.56(8.60,40.58) | 475.28(315.71,752.87) | 12.74(8.46,20.19) | -31.34(-62.72,37.78) | -1.24(-1.36,-1.12) |
| Republic of Singapore | 154.13(99.01,225.29) | 10.21(6.56,14.93) | 140.15(78.43,230.89) | 7.29(4.08,12.00) | -28.66(-53.90,4.61) | -0.91(-1.03,-0.79) |
| Republic of Slovenia | 70.95(54.46,96.26) | 9.26(7.11,12.56) | 30.53(20.14,44.15) | 5.36(3.54,7.75) | -42.08(-61.08,-15.41) | -1.82(-1.93,-1.70) |
| Republic of South Africa | 3604.57(2933.18,5014.82) | 22.91(18.64,31.87) | 3973.25(3182.61,4841.84) | 16.39(13.13,19.97) | -28.46(-46.60,-8.97) | -0.86(-1.71,0.00) |
| Republic of South Sudan | 374.46(184.38,1205.50) | 16.22(7.99,52.22) | 658.25(347.93,1447.03) | 18.29(9.67,40.21) | 12.75(-35.06,97.67) | 0.22(-0.03,0.48) |
| Republic of Sudan | 1774.86(780.15,3628.05) | 23.29(10.24,47.61) | 2513.90(1283.84,4747.48) | 13.60(6.95,25.68) | -41.61(-65.05,2.14) | -1.75(-1.87,-1.63) |
| Republic of Suriname | 57.37(37.14,70.56) | 35.23(22.81,43.33) | 42.54(31.16,56.81) | 19.82(14.52,26.46) | -43.74(-60.39,-10.93) | -1.59(-2.03,-1.14) |
| Republic of Tajikistan | 612.78(401.60,841.34) | 28.98(18.99,39.79) | 760.34(433.38,1638.08) | 18.23(10.39,39.27) | -37.10(-65.47,26.43) | -2.16(-2.60,-1.72) |
| Republic of the Congo | 179.40(81.91,283.75) | 18.94(8.65,29.95) | 342.76(224.02,506.36) | 15.47(10.11,22.85) | -18.31(-50.19,69.90) | -0.62(-0.83,-0.41) |
| Republic of the Gambia | 103.80(51.42,271.02) | 27.53(13.64,71.88) | 170.17(104.42,259.83) | 17.01(10.44,25.98) | -38.20(-70.91,26.19) | -2.08(-2.41,-1.74) |
| Republic of the Marshall Islands | 4.82(2.53,7.23) | 28.10(14.76,42.13) | 4.42(2.31,6.60) | 18.62(9.74,27.81) | -33.72(-61.27,7.83) | -1.12(-1.22,-1.02) |
| Republic of the Niger | 760.26(369.91,2587.08) | 27.31(13.29,92.95) | 1354.30(745.29,3373.57) | 15.19(8.36,37.83) | -44.40(-65.66,-4.44) | -2.35(-2.66,-2.05) |
| Republic of the Philippines | 7365.48(4874.39,9506.93) | 28.42(18.81,36.68) | 8613.91(6712.40,10313.60) | 18.23(14.21,21.83) | -35.85(-51.57,-13.59) | -1.27(-1.39,-1.16) |
| Republic of the Union of Myanmar | 14637.89(7362.85,24377.34) | 85.27(42.89,142.00) | 7434.27(4391.08,12387.21) | 33.07(19.53,55.10) | -61.22(-75.89,-30.62) | -3.47(-3.67,-3.26) |
| Republic of Trinidad and Tobago | 136.25(120.35,154.22) | 27.18(24.00,30.76) | 86.32(66.95,112.21) | 17.34(13.45,22.54) | -36.19(-50.83,-16.36) | -0.91(-1.25,-0.58) |
| Republic of Tunisia | 397.92(253.08,631.93) | 11.57(7.36,18.37) | 320.46(188.18,514.83) | 7.36(4.32,11.83) | -36.34(-57.39,-3.43) | -1.63(-1.69,-1.57) |
| Republic of Turkey | 2586.03(1537.65,3963.28) | 10.81(6.43,16.57) | 1791.22(1092.37,2755.05) | 5.62(3.43,8.65) | -48.00(-71.00,-9.98) | -2.31(-2.50,-2.12) |
| Republic of Uganda | 593.35(293.68,1250.43) | 9.25(4.58,19.49) | 1736.88(1045.89,2720.23) | 10.10(6.08,15.82) | 9.17(-33.46,88.64) | -0.28(-0.57,0.01) |
| Republic of Uzbekistan | 1834.33(1528.67,2215.51) | 21.37(17.81,25.81) | 1022.98(750.95,1361.20) | 7.45(5.47,9.91) | -65.15(-75.54,-52.27) | -3.92(-4.49,-3.35) |
| Republic of Vanuatu | 15.03(6.60,27.89) | 25.66(11.27,47.62) | 27.49(15.11,45.93) | 22.05(12.12,36.85) | -14.05(-55.48,57.40) | -0.70(-0.84,-0.56) |
| Republic of Yemen | 669.96(277.95,1418.32) | 14.57(6.05,30.85) | 1398.57(778.58,2218.21) | 10.16(5.66,16.12) | -30.26(-62.24,19.34) | -1.42(-1.72,-1.13) |
| Republic of Zambia | 590.87(366.44,956.57) | 19.47(12.08,31.53) | 1292.57(844.41,1898.26) | 15.97(10.43,23.46) | -17.98(-53.30,34.58) | -0.78(-1.12,-0.45) |
| Republic of Zimbabwe | 673.37(402.83,1467.51) | 16.98(10.16,37.01) | 1531.41(898.85,2535.71) | 24.16(14.18,40.01) | 42.27(-45.53,167.57) | 1.79(1.19,2.39) |
| Romania | 926.70(731.48,1192.97) | 10.67(8.42,13.73) | 277.13(164.44,426.89) | 5.14(3.05,7.92) | -51.80(-68.32,-31.78) | -2.25(-2.53,-1.97) |
| Russian Federation | 8235.99(7253.80,9622.66) | 14.16(12.47,16.54) | 3059.55(2329.30,4034.19) | 6.58(5.01,8.68) | -53.50(-61.15,-45.54) | -2.68(-2.86,-2.50) |
| Saint Kitts and Nevis | 5.74(4.83,6.85) | 33.20(27.94,39.62) | 3.38(2.49,4.66) | 14.85(10.97,20.50) | -55.26(-66.95,-38.84) | -2.49(-3.36,-1.62) |
| Saint Lucia | 15.56(13.75,17.59) | 27.66(24.44,31.26) | 13.39(10.76,16.42) | 20.25(16.27,24.84) | -26.78(-41.34,-9.70) | -0.37(-0.90,0.17) |
| Saint Vincent and the Grenadines | 8.56(7.20,9.98) | 18.63(15.67,21.72) | 7.15(5.74,8.75) | 17.31(13.89,21.18) | -7.07(-26.02,16.57) | 0.30(-0.15,0.75) |
| Slovak Republic | 155.48(110.03,216.82) | 7.60(5.37,10.59) | 102.56(61.66,153.51) | 5.99(3.60,8.97) | -21.10(-46.99,20.43) | -0.62(-0.74,-0.49) |
| Socialist Republic of Viet Nam | 5461.08(3316.12,8683.97) | 19.15(11.63,30.45) | 3374.59(2114.41,5005.46) | 8.79(5.51,13.04) | -54.10(-72.56,-22.21) | -2.64(-2.78,-2.50) |
| Solomon Islands | 19.92(9.26,36.44) | 15.53(7.22,28.41) | 36.15(21.15,55.02) | 13.22(7.73,20.11) | -14.90(-49.80,47.58) | -0.45(-0.55,-0.34) |
| State of Eritrea | 336.09(155.41,673.95) | 25.93(11.99,52.01) | 602.21(344.96,920.39) | 21.51(12.32,32.87) | -17.07(-49.58,38.93) | -0.63(-0.73,-0.53) |
| State of Israel | 108.03(64.07,165.00) | 5.65(3.35,8.63) | 165.29(89.07,281.70) | 4.97(2.68,8.48) | -12.02(-44.12,29.76) | -0.43(-0.49,-0.37) |
| State of Kuwait | 49.99(31.43,74.74) | 5.91(3.72,8.84) | 105.56(61.10,168.16) | 4.97(2.88,7.92) | -15.93(-44.91,21.07) | -0.39(-0.71,-0.08) |
| State of Libya | 267.52(150.55,488.05) | 15.92(8.96,29.05) | 293.62(182.97,421.07) | 9.79(6.10,14.03) | -38.54(-68.75,0.30) | -1.47(-1.75,-1.18) |
| State of Qatar | 22.28(14.69,31.43) | 9.42(6.21,13.29) | 92.14(47.79,154.04) | 5.58(2.89,9.32) | -40.83(-66.50,-6.56) | -1.83(-1.95,-1.72) |
| Sultanate of Oman | 70.74(46.52,108.55) | 8.53(5.61,13.08) | 124.91(70.67,209.93) | 5.40(3.05,9.07) | -36.70(-62.54,4.45) | -1.20(-1.29,-1.12) |
| Swiss Confederation | 227.20(161.53,310.54) | 8.62(6.13,11.79) | 188.19(117.26,266.25) | 6.78(4.22,9.59) | -21.39(-45.46,9.42) | -0.69(-0.76,-0.63) |
| Syrian Arab Republic | 820.85(571.78,1096.43) | 17.11(11.92,22.85) | 483.95(319.11,691.28) | 9.51(6.27,13.59) | -44.39(-65.94,-5.65) | -1.97(-2.15,-1.79) |
| Taiwan (Province of China) | 435.11(359.65,548.96) | 4.72(3.90,5.95) | 236.27(162.07,325.07) | 3.13(2.15,4.31) | -33.60(-47.29,-20.30) | -1.60(-1.76,-1.44) |
| Togolese Republic | 381.24(228.09,797.80) | 27.81(16.64,58.20) | 478.29(319.59,690.57) | 14.22(9.50,20.52) | -48.88(-74.70,-13.63) | -2.44(-2.64,-2.24) |
| Tokelau | 0.10(0.06,0.16) | 16.84(9.91,27.38) | 0.06(0.04,0.08) | 11.47(8.39,15.80) | -31.86(-59.54,16.81) | -1.84(-2.07,-1.61) |
| Turkmenistan | 389.21(323.87,457.84) | 25.35(21.10,29.83) | 203.96(156.37,273.35) | 9.81(7.52,13.14) | -61.32(-70.98,-46.95) | -3.88(-4.60,-3.16) |
| Tuvalu | 0.97(0.50,1.45) | 26.88(13.89,40.27) | 0.76(0.47,1.09) | 15.26(9.55,21.98) | -43.23(-65.50,-2.67) | -1.84(-1.88,-1.79) |
| Ukraine | 2585.19(2133.53,3106.77) | 13.61(11.23,16.36) | 852.24(554.73,1239.88) | 6.18(4.02,8.99) | -54.58(-68.85,-39.03) | -2.68(-3.19,-2.17) |
| Union of the Comoros | 31.68(14.65,80.78) | 18.34(8.48,46.76) | 46.32(28.25,78.70) | 14.95(9.12,25.40) | -18.47(-55.12,62.70) | -1.36(-2.04,-0.68) |
| United Arab Emirates | 65.27(40.11,97.78) | 6.83(4.20,10.23) | 203.33(108.29,337.02) | 5.06(2.70,8.39) | -25.87(-59.41,33.38) | -0.90(-1.01,-0.80) |
| United Kingdom of Great Britain and Northern Ireland | 1218.76(898.66,1629.60) | 5.83(4.30,7.80) | 1107.09(786.54,1502.38) | 5.09(3.62,6.91) | -12.72(-19.99,-5.35) | -0.38(-0.59,-0.17) |
| United Mexican States | 9144.41(8504.88,9949.63) | 25.64(23.85,27.90) | 14045.94(12590.83,15836.73) | 27.27(24.44,30.74) | 6.34(-4.07,17.49) | 0.64(0.31,0.98) |
| United Republic of Tanzania | 1405.04(823.43,2969.68) | 14.50(8.50,30.65) | 3230.05(2007.16,6998.38) | 13.84(8.60,29.99) | -4.54(-40.55,62.42) | 0.07(-0.16,0.31) |
| United States of America | 6878.36(5108.89,9090.25) | 6.73(5.00,8.90) | 6304.32(4881.60,7870.60) | 5.66(4.39,7.07) | -15.86(-28.18,-2.58) | -0.58(-0.65,-0.51) |
| United States Virgin Islands | 11.86(8.99,15.52) | 29.95(22.70,39.18) | 5.67(3.88,8.97) | 24.49(16.79,38.79) | -18.21(-47.22,33.95) | -0.07(-0.30,0.17) |

AYAs = adolescents and young adults, PC = percentage change, EAPC = estimated annual percentage changes.
